# Sulfinamide Crossover Reaction

**DOI:** 10.1021/acs.joc.4c00572

**Published:** 2024-05-24

**Authors:** Vladimír Nosek, Jiří Míšek

**Affiliations:** Department of Organic Chemistry, Faculty of Science, Charles University in Prague, Hlavova 2030/8, 12843 Prague 2, Czech Republic

## Abstract

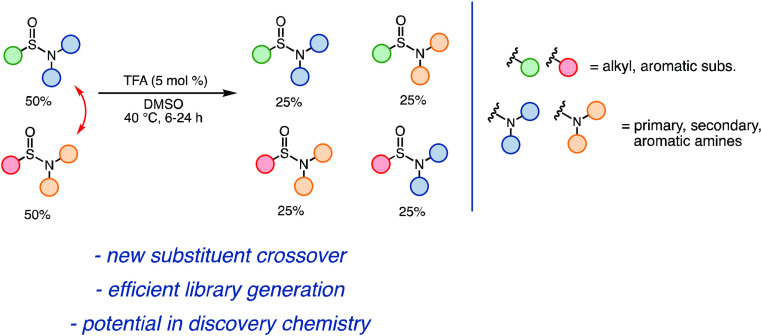

This study unveils a new catalytic crossover reaction of sulfinamides. Leveraging mild acid catalysis, the reaction demonstrates a high tolerance to structural variations, yielding equimolar products across diverse sulfinamide substrates. Notably, small sulfinamide libraries can be selectively oxidized to sulfonamides, providing a new platform for ligand optimization and discovery in medicinal chemistry. This crossover chemotype provides a new tool for high-throughput experimentation in discovery chemistry.

A crossover reaction is typically associated with biological systems in which two DNA molecules can swap pieces of their sequences (Figure 1A). This enzyme-catalyzed biological crossover is an essential part of sexual reproduction and one of the major drivers of the evolution of sexual organisms.^1−5^ In chemistry, certain functional group reactions follow the same path of crossing over their substituents (Figure 1B).

**Figure 1:**
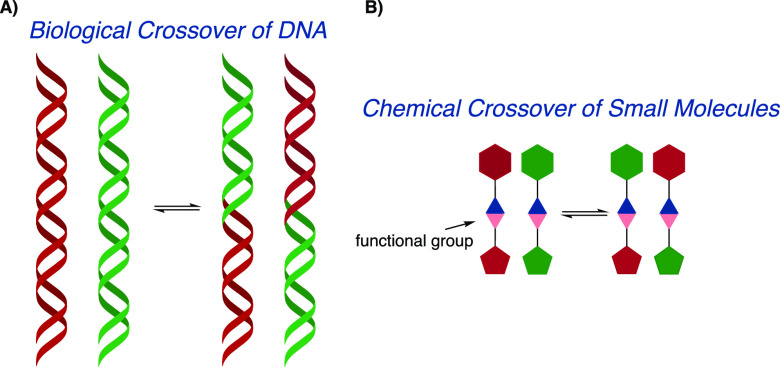
Schematic depiction of a crossover reaction in biology (A) and chemistry (B).

For instance, alkene cross-metathesis and disulfide exchange are examples of such reactions that enable swapping substituents under metal and thiol catalysis, respectively.^6−15^ Several such reactions in chemistry have been heavily utilized in the field of dynamic combinatorial libraries (Figure 2).^16−18^ Various synthetic receptors for metals and small molecules have been developed. Also, polymer chemistry and metal catalyst development have benefited from these approaches.^19−31^ Dynamic combinatorial approaches also showed some promising results in the field of ligand-drug discovery for medicinal purposes. However, the dynamic nature of the functional groups (disulfides, acetals, imines, etc.) or the chemical nature of the functional groups as such (alkenes, esters, etc.) is often not compatible with the required drug-like characteristics. Therefore, these functional groups serve as crossover points that need to be decorated with substructures with drug-like characteristics.^32−34^ The hits from the screenings have to be converted to more drug-like molecules that can represent a formidable challenge. Also, modifications of the functional groups to stop the crossover (e.g., reduction of imines to amines) belong to strategies to tackle these issues. Furthermore, functional group tolerance, limited complexity, and differences in the thermodynamic stability of products under equilibrating conditions can further complicate the efficient search for ligands for biomolecules. Therefore, improvements in the chemistry of crossover reactions are of high importance in the field of discovery chemistry. Here, we describe a new catalytic crossover reaction of sulfinamides that tolerates a diverse set of structural features. Furthermore, the sulfinamide libraries can be easily oxidized to the corresponding sulfonamides, which are medicinally relevant building blocks.

**Figure 2:**
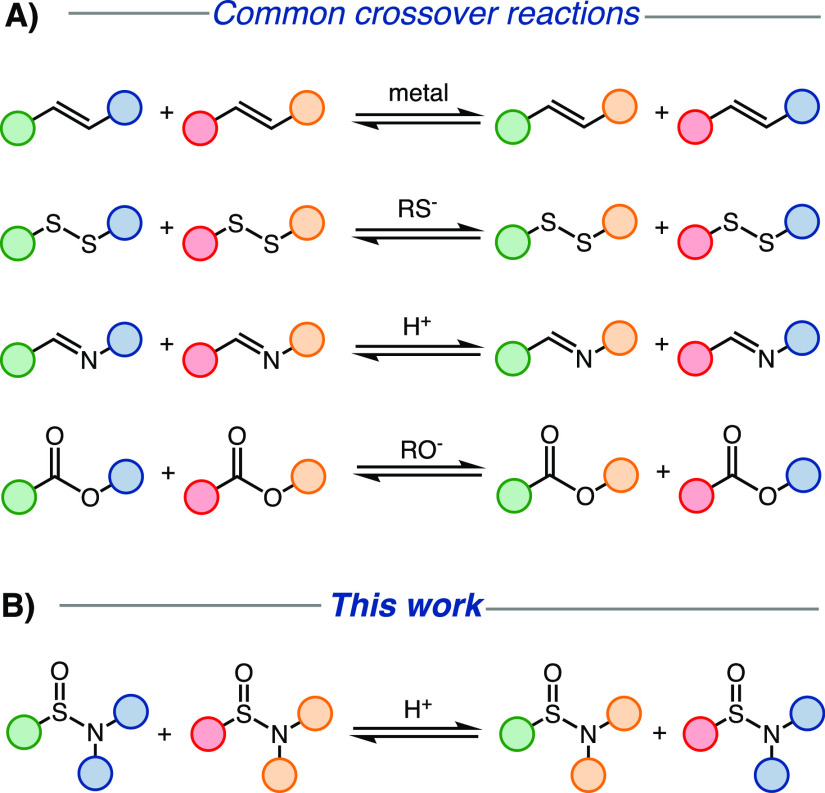
(A) Selected examples of crossover (exchange) reactions utilized in dynamic combinatorial chemistry. (B) Crossover of sulfinamides presented in this article.

Recently, we have developed a straightforward and general one-pot protocol for the synthesis of sulfinamides from sulfonyl chlorides and thiols.^35^ Unlike sulfonamides, sulfinamides contain a nitrogen atom that is relatively nucleophilic, and thus, sulfinamides can form stable imine-like adducts with carbonyl compounds. This feature is cleverly utilized in Ellman’s sulfinamide for the asymmetric synthesis of amines.^36,37^ We hypothesized that the nucleophilic nature of the nitrogen, along with the electrophilic character of the S=O bond, could lead to the crossover reaction of two different sulfinamides under specific conditions. We tested various conditions, and delightfully, mild acid catalysis provided the crossover products almost quantitatively. Model tertiary sulfinamides **1a** and **2b** were treated with 5 mol % of TFA in DMSO at 40 °C. After 6 h, the crossover reaction provided four sulfinamides **1a**, **2b**, **1b**, and **2a** in almost equimolar amounts (Figure 3).

**Figure 3:**
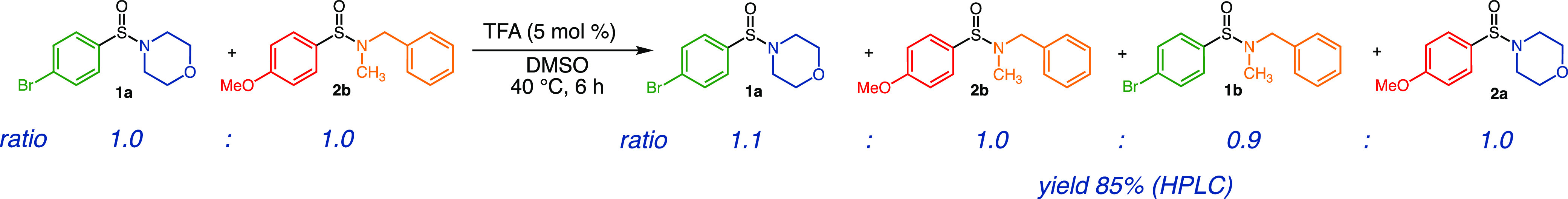
Development of the first acid-catalyzed sulfinamide crossover reaction.

No significant amount of side products was observed by HPLC analysis. To the best of our knowledge, this kind of reaction has never been described before and represents a new type of a crossover system. The literature describes acid-catalyzed hydrolysis/alcoholysis of sulfinamides, and recently, three groups have reported transulfinamidation of primary sulfinamides with an excess of (hydroxyl)amines under thermal conditions or metal catalysis.^38−43^ These are the closest examples of similar sulfinamide reactivity. Encouraged by the initial results, we set out to determine the scope of this reaction. First, the N-substituents were varied. Various tertiary sulfinamides with cyclic and acyclic aliphatic N-substituents underwent the crossover reaction with high yields, resulting in an almost equimolar mixture of products as determined by HPLC (Figure 4). Sulfinamides **1f** and **2f** bearing a basic tertiary amino group can also serve as substrates for the crossover when 0.4 equiv of the TFA catalyst is utilized. Tertiary and secondary sulfinamides also undergo crossover, as exemplified by Figure 4 entry 6. Interestingly, secondary sulfinamides **1h** and **2h** with an electron-poor aromatic Nsubstituent also provided the crossover products. Next, the combination of substrates **1a** and **3b** with aromatic and aliphatic S substituents was tested. Despite the slower reaction rate (24 h reaction), a significant amount of the crossover products was also observed (entry 8). All crossover reactions of two substrates were performed in both directions, starting with a different set of substrates. A comparison of these experiments indicates that regardless of the starting substrates, the reactions lead to an equilibrium of almost equimolar amounts of products. This indicates that the thermodynamic stability of the given sulfinamides is similar, and the steric/electronic effects do not play a major role, at least for the substrates tested. It should be noted that the reaction also proceeds in other solvents than DMSO, such as toluene or acetonitrile. However, DMSO turned out to be the most general solvent in terms of the solubility of both substrates and products. Oftentimes, substrates were well soluble in each solvent, however, the resulting mixture of products after the crossover led to the crystallization of products that complicated further analysis. In terms of limitations, primary sulfinamides undergo the crossover reaction, however, with lower yields (see the Supporting Information). Also, sulfinamides with electron-rich aromatic N substituents led to unwanted side products of the same mass, as indicated by HPLC-MS analysis (see the Supporting Information). These observations are consistent with the reports on acid-catalyzed rearrangement of N-aromatic sulfinamides to sulfoxides.^44^ Also, we tested the crossover reaction conditions between sulfinamide and sulfonamide. As expected, no significant reaction was observed after a prolonged reaction time. This experiment indicated that the crossover reaction is, indeed, specific to sulfinamides. In the next experiment, we tested the crossover reaction of three different sulfinamides, leading to nine possible products. All nine products were synthesized, and the crossover reaction was performed with six sets of three different sulfinamides. All six reactions led to the formation of nine expected products with a very similar ratio of products and close to equimolar representation (Figure 5). HPLC traces of the crossover reactions indicated that regardless of the initial combination of substrates, a good yield of all combinatorial products with only a minimal amount of side products is achieved. These experiments corroborated that the sulfinamide crossover is suitable for generation of a new type of dynamic combinatorial libraries. It should be pointed out that sulfinamides are generally stable compounds in neutral and basic aqueous environment. Therefore, the resulting libraries are compatible with biochemical binding assays. The limited stability in acidic aqueous buffers and possible biological oxidation can be limiting factors in more complex biological experiments. We hypothesized that freezing the libraries by oxidation to sulfonamides would provide stable compounds that are relevant in medicinal chemistry. Therefore, we subjected the mini-library of nine sulfinamides to mild oxidation with mCPBA.^41^ Delightfully, the oxidation proceeded smoothly, providing an expected set of nine sulfonamides with minimum side products (see the Supporting Information). Also, we confirmed the expected boost in stability of sulfonamides compared with sulfinamides under acidic aqueous conditions (see the Supporting Information).

**Figure 4:**
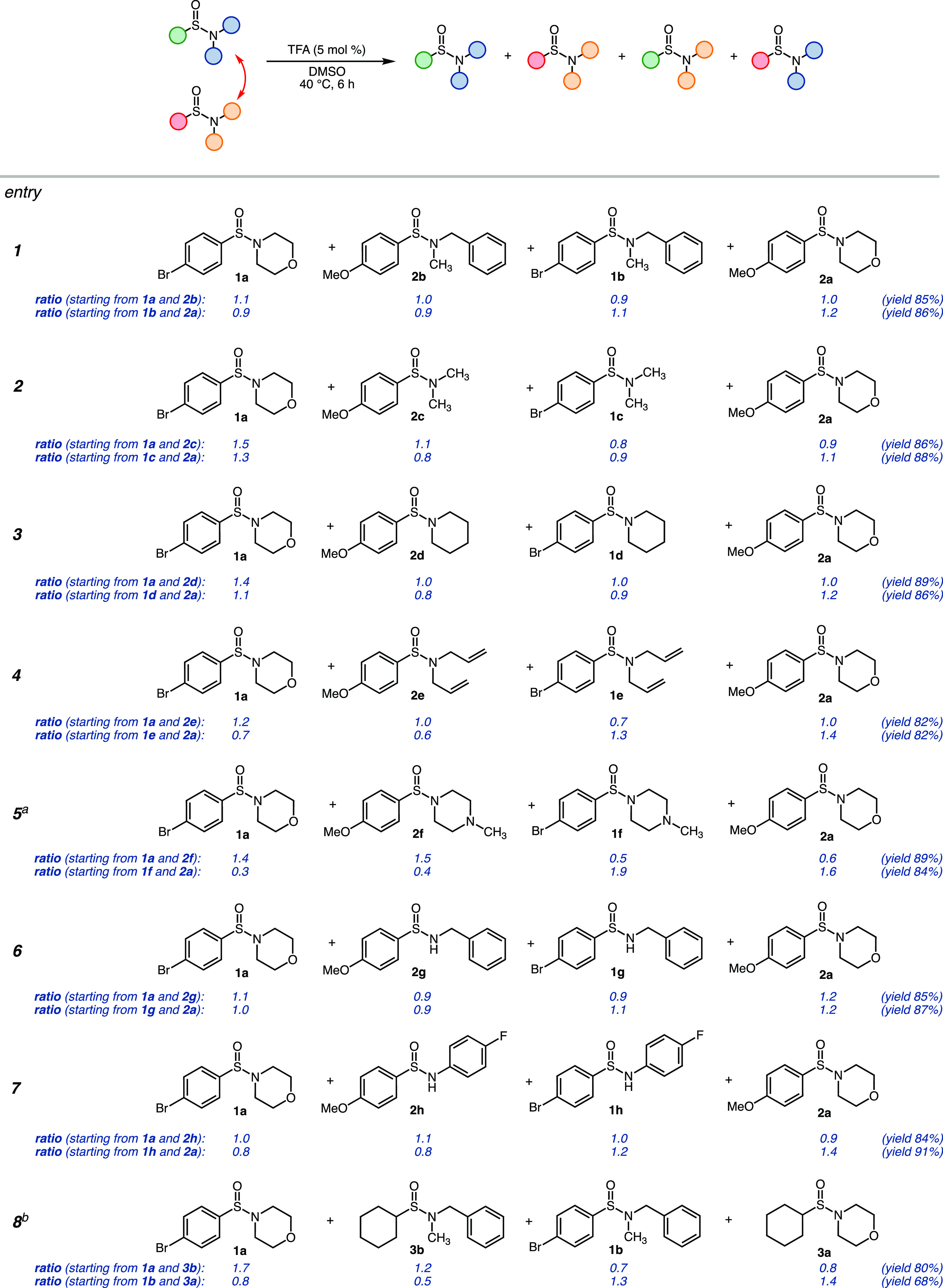
Substrate scope of the crossover reaction. Molar ratios of products and their yields were determined by HPLC. ^a^ 40 mol % TFA was used in the reaction. ^b^ The reaction time was 24 h.

**Figure 5:**
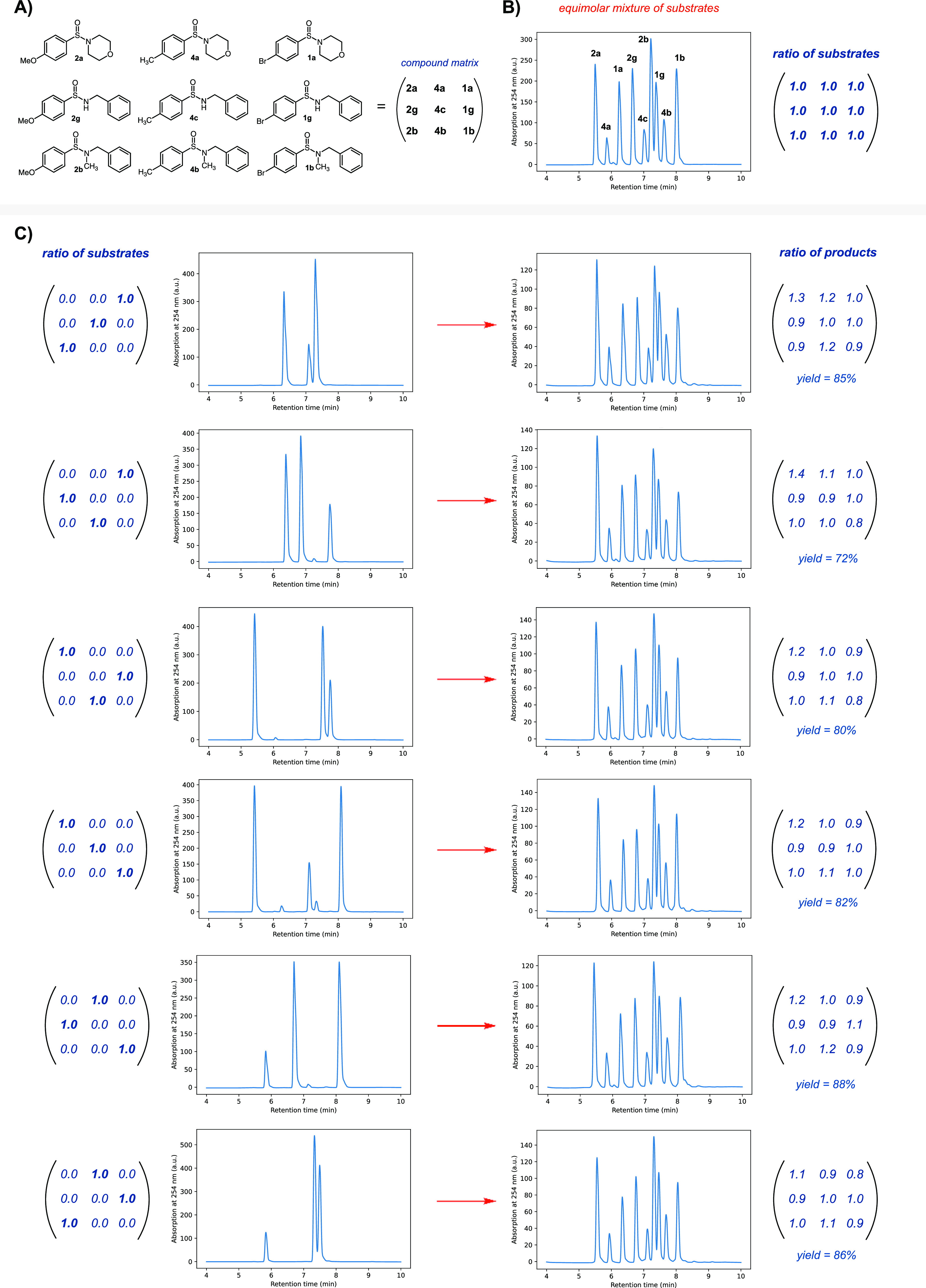
Summary of the crossover of three different sulfinamides. (A) Structures of all sulfinamides utilized in the experiments. (B) HPLC trace of an equimolar amount of sulfinamide substrates/products. (C) HPLC traces of six different crossover reactions leading to the mixture of the same products. Conditions of the reaction: 200 mM sulfinamides and 6.6 mM TFA in DMSO, 40 °C, 24 h.

The crossover reaction described offers a new principle that has potential in the field of discovery chemistry. The simplicity and generality of the new crossover reaction make it suitable for applications spanning fields from supramolecular to medicinal chemistry. Given the broad structural diversity of commercially available feedstocks for the synthesis of sulfinamides (amines, sulfonyl chlorides, and thiols), a straightforward generation of sulfinamide libraries for high-throughput discovery of new functions is anticipated. Sulfinamides are compatible with both aqueous and nonaqueous environments and represent 3D surrogates of amides. Moreover, their facile oxidation to sulfonamides adds to the potential of ligand optimization and discovery. Further investigations into the mechanism of the reaction using enantiomerically pure sulfinamides as stereochemical probes are underway.

## Data Availability

The data underlying this study are available in the published article and its Supporting Information.

## Supporting Information Available

Detailed experimental conditions and analytical data including HPLC traces and reprints of NMR spectra (PDF)
