# Supplementary material for: Sulfinamide Crossover Reaction
Source: J Org Chem. 2024 May 24;89(11):7927–32. doi: 10.1021/acs.joc.4c00572 (PMC11165587; doi:10.1021/acs.joc.4c00572)
Supplement: Supplementary file 1 — jo4c00572_si_001.pdf [file jo4c00572_si_001.pdf]

Supporting information

A Sulfinamide Crossover Reaction

Vladimír Nosek and Jiří Míšek*

Department of Organic Chemistry, Faculty of Science, Charles University in Prague, Hlavova 2030/8, 12843 Prague 2 (Czech Republic), E-mail: misek@natur.cuni.cz

Table of Contents:

1. General 2

2. General Procedures for the Preparation of Sulfinamides 2

3. General Procedure for the Preparation of Sulfonamides 8

4. General Procedure for the Crossover Reaction 10

5. Reaction of Sulfinamide **1a** with *N*-methyl-1-phenylmethanamine Under Crossover Condition 11

6. Oxidation of a Mini-Library of Sulfinamides to Sulfonamides 11

7. The Stability Test of Sulfinamide and Sulfonamide Libraries in Aqueous Solutions 12

8. Preliminary Solvent and Acid Screening 12

9. Stability Test of *N*-aromatic Sulfinamide **4i** Under Crossover Conditions 12

10. References 13

11. HPLC Chromatograms 14

12. Time course of the Reaction of **1a** and **2b** 34

13. Extracted HPLC-MS Chromatograms of Solvent and Acid Screens 37

14. Extracted HPLC-MS Chromatograms of Sulfinamide **4i** Under Acidic Conditions 39

15. ^{1}H and ^{13}C NMR Spectra 40

1. General

All starting reagents were commercially available and of analytical purity, which were used without further treatment unless otherwise stated. Sulfuryl chloride was freshly distilled at 68 °C - 69 °C under argon atmosphere. Thionyl chloride was freshly distilled at 76 °C - 78 °C under argon atmosphere. Zinc powder was activated using a standard method.[1] Solvents were dried according to standard methods. ^{1}H and ^{13}C{^{1}H} NMR spectra were recorded on Varian UNITY INOVA-300 or Bruker Avance-400 instruments. ^{1}H NMR spectra were recorded at 400 MHz or 300 MHz. ^{13}C{^{1}H} NMR spectra were recorded at 101 MHz and were ^{1}H decoupled. Chemical shifts (δ) are reported in ppm relative to solvent ($CDCl_3$: δC = 77.0 ppm, $(CD_3)_2SO$: δC = 39.5 ppm) or residual solvent peak ($CHCl_3$: δH = 7.26 ppm, $(CH_3)_2SO$: δH = 2.50 ppm). ^{19}F NMR spectra were obtained at 376 MHz. Chemical shifts for ^{19}F NMR are reported in terms of chemical shift in reference to an internal standard (fluorobenzene set to δ –112.96 ppm). Sulfinamide **4k** was obtained from a commercial source. Accurate mass measurements (HRMS) were obtained by ESI on Agilent 6530 Q-TOF MS spectrometer or Bruker Compact Q-TOF spectrometer. Analytical HPLC was performed under the following conditions: Agilent Eclipse plus C18 column (3.5 µL, 4.6×100 mm); UV/Vis detection at λ_{obs} = 254 nm or 210 nm; flow rate 0.4 mL/min; gradient elution method (0.1% aqueous formic acid – CH_3CN from 95:5 to 0:100 in 13 min). Analytical TLC was performed using a pre-coated silica gel 60 Å F_{254} plates (0.2 mm thickness) and visualized by irradiation with UV light at 254 nm and by dipping in a stain solution ($KMnO_4$ or AMC) followed by heating. Preparative column chromatography was carried out using silica gel 60 Å (particle size 0.063–0.200 mm). Purifications by HPLC were performed under the following conditions: Agilent ZORBAX SB-C18 column (21.2×150 mm); UV/Vis detection at λ_{obs} = 254 nm and 220 nm; flow rate 9 mL/min; a gradient elution method (H_2O – CH_3CN from 50:50 to 0:100 in 20 min). Infrared spectra were recorded on Nicolet Avatar 370 FTIR ATR (thin film). IR absorptions are given in wavenumbers as cm^{-1}. Crossover reactions were carried at 40 °C in MyTEMPMini incubator without mixing.

2. General Procedures for the Preparation of Sulfinamides

Sulfinamides were prepared according to known protocols published previously.[2]

2.1. General procedure A (oxidative pathway)

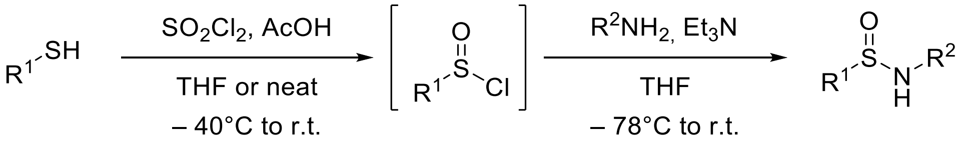

To an oven dried Schlenk flask, a thiol (1 mmol), AcOH (120 µL, 2.1 mmol, 2.1 eq.) and THF (1 mL) were added. SO_2Cl_2 (275 µL, 3.5 mmol, 3.5 eq.) was added dropwise within 10 min at –40 °C under argon atmosphere. The reaction mixture was allowed to warm to rt and stirred for additional 120 min. Volatiles were evaporated under reduced pressure at rt and the residue was dissolved in THF (5 mL). The resulting solution was added dropwise to the solution of an amine (1.5 mmol, 1.5 eq.) and Et_3N (280 µL, 2 mmol, 2.0 eq.) in THF (2.5 mL) at –78 °C and the reaction mixture was stirred at rt overnight. Then the reaction mixture was diluted with CH_2Cl_2 (20 mL) and washed with saturated aq. $NaHCO_3$ solution (10 mL). The aqueous phase was extracted with CH_2Cl_2 (2 × 20 mL). The combined organic phase was washed with brine (15 mL), dried over anhydrous $MgSO_4$ and

concentrated under reduced pressure. Purification with column chromatography on silica gel afforded the product.

2.2. General procedure B (reductive pathway)

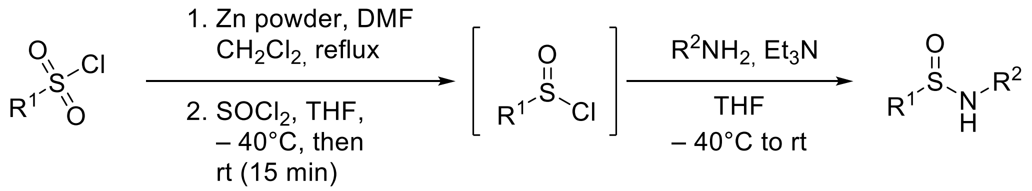

Sulfonyl chloride (0.81 mmol) was dissolved in CH_2Cl_2 (3 mL) in 25 mL round-bottom flask and DMF (94 µL, 1.22 mmol, 1.5 eq.) was added. The resulting mixture was stirred for 10 minutes. Powdered zinc (58.5 mg, 0.89 mmol, 1.1 eq.) was added (caution: vigorous initial reaction) and the mixture was refluxed until all the starting sulfonyl chloride was consumed (typically 1 h). Then the mixture was filtered, and residues washed with MeOH (10 mL). The filtrate was concentrated under reduced pressure, redissolved in THF (3 mL) and $SOCl_2$ (118 µL, 1.63 mmol, 2.0 eq.) was added dropwise at -40 °C. The resulting mixture was stirred for 45 min at -40 °C, then the cooling bath was removed, and the reaction mixture was stirred for additional 15 min at rt. Volatiles were evaporated under reduced pressure, the residue dissolved in THF (3 mL) and Et_3N (170 µL, 1.22 mmol, 1.5 eq.) and benzylamine (133 µL, 1.22 mmol, 1.5 eq.) were added at -40 °C. The reaction mixture was stirred at rt overnight, then diluted with CH_2Cl_2 (10 mL) and washed with saturated aq. $NaHCO_3$ solution (20 mL). The combined organic phase was dried over anhydrous $MgSO_4$, concentrated under reduced pressure. Purification with column chromatography on silica gel afforded the product.

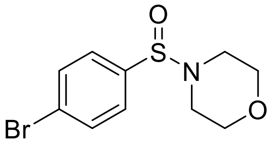

4-((4-bromophenyl)sulfinyl)morpholine (**1a**).[3] Prepared according to the general procedure A using 4-bromothiophenol (567 mg, 3 mmol). Purification by column chromatography (50% of EtOAc in cyclohexane) afforded the product as a white solid (736 mg, 85%).^{1}H NMR (400 MHz, $CDCl_3$) δ 7.70 – 7.62 (m, 2H), 7.58 – 7.51 (m, 2H), 3.79 – 3.65 (m, 4H), 3.17 (ddd, J = 12.1, 6.1, 3.4 Hz, 2H), 2.96 (ddd, J = 12.1, 6.1, 3.3 Hz, 2H). The spectra were in agreement with reported data.[3]

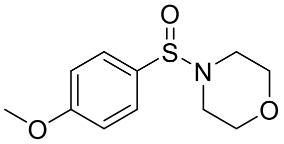

4-((4-methoxyphenyl)sulfinyl)morpholine (**2a**).[4] Prepared according to the general procedure A using 4-methoxythiophenol (140 mg, 1 mmol). Purification by column chromatography (50% of EtOAc in cyclohexane) afforded the product as a white solid (201mg, 83%).^{1}H NMR (400 MHz, $CDCl_3$) δ 7.63 – 7.53 (m, 2H), 7.06 – 6.97 (m, 2H), 3.86 (s, 3H), 3.68 – 3.64 (m, 4H), 3.14 (ddd, J = 12.1, 6.1, 3.4 Hz, 2H), 2.96 (ddd, J = 12.1, 6.1, 3.4 Hz, 2H). The spectra were in agreement with reported data.[4]

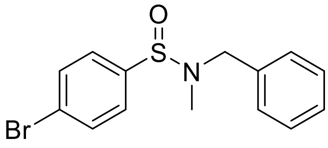

N-benzyl-4-bromo-N-methylbenzenesulfinamide (**1b**). Prepared according to the general procedure A using 4-bromothiophenol (284 mg, 1.5 mmol). Purification by column chromatography (33% of EtOAc in cyclohexane) afforded the product as white solid (366 mg, 75%) ^{1}H NMR (400 MHz, $CDCl_3$) δ 7.70 – 7.63 (m, 2H), 7.63 – 7.56 (m, 2H), 7.40 – 7.22 (m, 5H), 4.29 (d, *J* = 14.1 Hz, 1H), 4.13 (d, *J* = 14.1 Hz, 1H), 2.49 (s, 3H). ^{13}C{^{1}H} NMR (101 MHz, $CDCl_3$) δ 143.0, 136.5, 132.2, 128.7, 128.5, 127.9, 127.9, 125.7, 55.9, 32.9. HRMS (ESI): *m/z* $[M+H]^+$ calcd for $C_{14}H_{15}BrNOS^+$ 324.0052; found 324.0053; IR (ν_{max}/cm^{-1}) 3060, 1568, 1464, 1082, 1057.

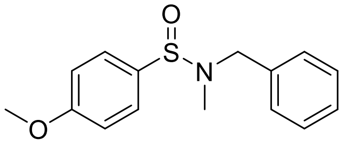

N-benzyl-4-methoxy-N-methylbenzenesulfinamide (**2b**). Prepared according to the general procedure A using 4-methoxythiophenol (210 mg, 1.5 mmol). Purification by column chromatography (50% of EtOAc in cyclohexane) afforded the product as a colorless oil (345mg, 84%). ^{1}H NMR (400 MHz, $CDCl_3$) δ 7.70 – 7.56 (m, 2H), 7.38 – 7.19 (m, 5H), 7.07 – 6.93 (m, 2H), 4.23 (d, *J* = 14.1 Hz, 1H), 4.10 (d, *J* = 14.1 Hz, 1H), 3.82 (s, 3H), 2.46 (s, 3H). ^{13}C{^{1}H} NMR (101 MHz, $CDCl_3$) δ 161.8, 136.9, 135.1, 128.6, 128.5, 127.8, 127.7, 114.3, 55.5, 55.4, 32.7. HRMS (ESI): *m/z* $[M+H]^+$ calcd for $C_{15}H_{18}NO_2S^+$ 276.1053; found 276.1042; IR (ν_{max}/cm^{-1}) 3465, 3087, 1591, 1493, 1442, 1250, 1086, 1065, 1026.

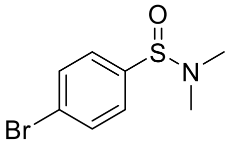

4-bromo-N,N-dimethylbenzenesulfinamide (**1c**). Prepared according to the general procedure A using 4-bromothiophenol (189 mg, 1 mmol). Purification by column chromatography (33% of EtOAc in cyclohexane) afforded the product as a yellowish solid (157 mg, 63%). ^{1}H NMR (400 MHz, $CDCl_3$) δ 7.68 – 7.61 (m, 2H), 7.55 – 7.47 (m, 2H), 2.67 (s, 6H). ^{13}C{^{1}H} NMR (101 MHz, $CDCl_3$) δ 142.8, 132.1, 127.8, 125.6, 37.3. HRMS (ESI): *m/z* $[M+H]^+$ calcd for $C_8H_{11}BrNOS^+$ 247.9739; found 247.9742; IR (ν_{max}/cm^{-1}) 3074, 1568, 1469, 1446, 1173; 1082; 1059, 1038.

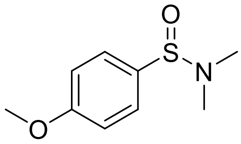

4-methoxy-N,N-dimethylbenzenesulfinamide (**2c**). Prepared according to the general procedure A using 4-methoxythiophenol (140 mg, 1 mmol). Purification by column chromatography (50% of EtOAc in cyclohexane) afforded the product as an oil (155 mg, 78%).^{1}H NMR (400 MHz, $CDCl_3$) δ 7.62 – 7.51 (m, 2H), 7.03 – 6.96 (m, 2H), 3.85 (s, 3H), 2.65 (s, 6H). ^{13}C{^{1}H} NMR (101 MHz, $CDCl_3$) δ 161.7, 134.9, 127.7, 114.2, 55.5, 36.9. HRMS (ESI): *m/z* $[M+H]^+$ calcd for $C_9H_{14}NO_2S^+$ 200.0740; found 200.0749; IR (ν_{max}/cm^{-1}) 3404, 2949, 1593, 1493, 1304, 1248, 1173, 1086, 1022.

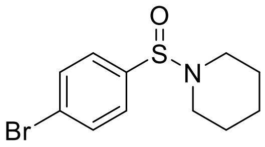

1-((4-bromophenyl)sulfinyl)piperidine (**1d**). Prepared according to the general procedure using A 4-bromothiophenol (95 mg, 0.5 mmol). Purification by column chromatography (33% of EtOAc in cyclohexane) afforded the product as a yellowish solid (82 mg, 57%). ^{1}H NMR (400 MHz, $CDCl_3$) δ 7.69 – 7.59 (m, 2H), 7.59 – 7.46 (m, 2H), 3.17 – 3.06 (m, 2H), 3.00 – 2.88 (m. 2H), 1.75 – 1.49

(m, 6H). $^{13}C\{^1H\}$ NMR (101 MHz, $CDCl_3$) δ 142.7, 132.0, 128.0, 125.4, 47.1, 26.2, 23.8. HRMS (ESI): *m/z* $[M+H]^+$ calcd for $C_{11}H_{15}BrNOS^+$ 288.0052; found 288.0038; IR (ν_{max}/cm^{-1}) 2945, 2839, 1570, 1466, 1099,1082, 1061, 1007.

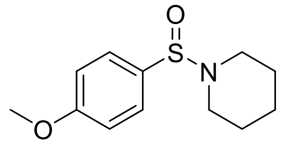

1-((4-methoxyphenyl)sulfinyl)piperidine (**2d**).[5] Prepared according to the general procedure A using 4-methoxythiophenol (70 mg, 0.5 mmol). Purification by column chromatography (from 50% to 100% of EtOAc in cyclohexane) afforded the product as a white solid (70 mg, 58%) ^1H NMR (400 MHz, $CDCl_3$) δ 7.62 – 7.52 (m, 2H), 7.06 – 6.96 (m, 2H), 3.86 (s, 3H), 3.16 – 3.03 (m, 2H), 3.03 – 2.88 (m, 2H), 1.70 – 1.46 (m, 6H). The spectra were in agreement with reported data.[5]

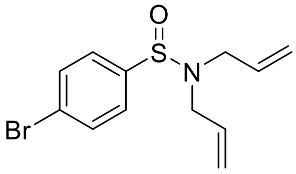

N,N-diallyl-4-bromobenzenesulfinamide (**1e**). Prepared according to the general procedure A using 4-bromothiophenol (94 mg, 0.5 mmol). Purification by column chromatography (25% of EtOAc in cyclohexane) afforded the product as an yellowish oil (106 mg, 65%). ^1H NMR (400 MHz, $CDCl_3$) δ 7.67 – 7.60 (m, 2H), 7.58 – 7.50 (m, 2H), 5.80 – 5.66 (m, 2H), 5.22 – 5.17 (m, 2H), 5.17 – 5.15 (m, 1H), 5.15 – 5.12 (m, 1H), 3.71 – 3.58 (m, 4H). $^{13}C\{^1H\}$ NMR (101 MHz, $CDCl_3$) δ 143.4, 133.8, 132.0, 128.0, 125.6, 119.0, 50.4. HRMS (ESI): *m/z* $[M+H]^+$ calcd for $C_{12}H_{15}BrNOS^+$ 300.0052; found 300.0047; IR (ν_{max}/cm^{-1}) 3078, 2914, 1738, 1641, 1572, 1468; 1383, 1086, 1063, 1007.

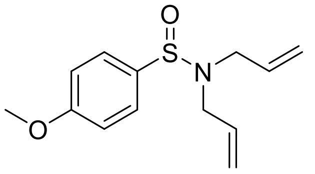

N,N-diallyl-4-methoxybenzenesulfinamide (**2e**). Prepared according to the general procedure A using 4-methoxythiophenol (70 mg, 0.5 mmol). Purification by column chromatography (from 33% to 50% of EtOAc in cyclohexane) afforded the product as an yellowish oil (106 mg, 84%). ^1H NMR (400 MHz, $CDCl_3$) δ 7.62 – 7.54 (m, 2H), 7.04 – 6.95 (m, 2H), 5.81 – 5.68 (m, 2H), 5.19 – 5.15 (m, 2H), 5.15 – 5.10 (m, 2H), 3.86 (s, 3H), 3.70 – 3.56 (m, 4H). $^{13}C\{^1H\}$ NMR (101 MHz, $CDCl_3$) δ 161.7, 135.4, 134.3 127.9, 118.6, 114.2, 55.5, 50.1. HRMS (ESI): *m/z* $[M+H]^+$ calcd for $C_{13}H_{18}NO_2S^+$ 252.1053; found 252.1040; IR (ν_{max}/cm^{-1}) 3460, 3078, 1593, 1493, 1304, 1248, 1086, 1065, 1026.

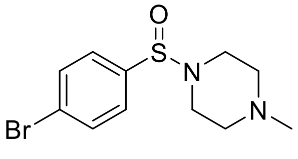

1-((4-bromophenyl)sulfinyl)-4-methylpiperazine (**1f**). Prepared according to the general procedure A using 4-bromothiophenol (94 mg, 0.5 mmol). Purification by column chromatography (10% of MeOH in EtOAc with 1% Et_3N) afforded the product as a white solid (108 mg, 71%). ^1H NMR (400 MHz, $CDCl_3$) δ 7.68 – 7.60 (m, 2H), 7.58 – 7.47 (m, 2H), 3.28 – 3.13 (m, 2H), 3.10 – 2.94 (m, 2H), 2.53 – 2.38 (m, 4H), 2.29 (s, 3H). $^{13}C\{^1H\}$ NMR (101 MHz, $CDCl_3$) δ 142.2, 132.1, 128.0, 125.7, 55.1, 46.0, 45.9. HRMS (ESI): *m/z* $[M+H]^+$ calcd for $C_{11}H_{16}BrN_2OS^+$ 303.0161; found 303.0151; IR (ν_{max}/cm^{-1}) 3390, 2937, 2864, 1574, 1450, 1313, 1417, 1082, 1061, 1005.

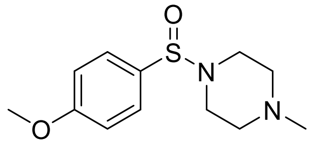

1-((4-methoxyphenyl)sulfinyl)-4-methylpiperazine (**2f**). Prepared according to the general procedure A using 4-methoxythiophenol (70 mg, 0.5 mmol). Purification by column chromatography (10% of MeOH in EtOAc with 1% Et_3N) afforded the product as a white solid (80 mg, 63%). ^{1}H NMR (400 MHz, $CDCl_3$) δ 7.60 – 7.53 (m, 2H), 7.04 – 6.97 (m, 2H), 3.86 (s, 3H), 3.24 – 3.13 (m, 2H), 3.08 – 2.99 (m, 2H), 2.54 – 2.40 (m, 4H), 2.29 (s, 3H). ^{13}C{^{1}H} NMR (101 MHz, $CDCl_3$) δ 161.8, 134.1, 127.8, 114.3, 55.5, 55.1, 45.9, 45.4. HRMS (ESI): *m/z* $[M+H]^+$ calcd for $C_{12}H_{19}N_2O_2S^+$ 255.1162; found 255.1152. IR (ν_{max}/cm^{-1}) 3020, 2956, 2790, 1563, 1495, 1450, 1250, 1088, 1065, 1022.

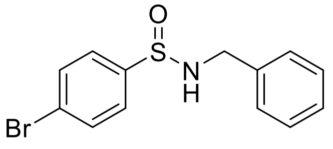

N-benzyl-4-bromobenzenesulfinamide (**1g**).[2] Prepared according to the general procedure A using 4-bromobenzenethiol (189 mg, 1 mmol). Purification by column chromatography (50 % of EtOAc in cyclohexane) afforded the product as a white solid (279 mg, 90 %). ^{1}H NMR (400 MHz, $CDCl_3$) δ 7.77 – 7.57 (m, 4H), 7.40 – 7.19 (m, 5H), 4.44 – 4.31 (m, 1H), 4.27 (dd, *J* = 13.4, 5.1 Hz, 1H), 3.90 (dd, *J* = 13.4, 7.1 Hz, 1H). The spectra were in agreement with reported data.[2]

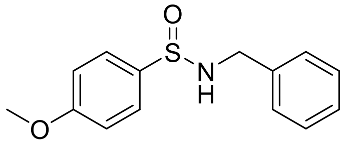

N-benzyl-4-methoxybenzenesulfinamide (**2g**).[2] Prepared according to the general procedure A using 4-methoxythiophenol (140 mg, 1 mmol). Purification by column chromatography (50 to 100 % of EtOAc in cyclohexane) afforded the product as a white solid (227 mg, 87 %). ^{1}H NMR (400 MHz, $CDCl_3$) δ 7.76 – 7.64 (m, 2H), 7.40 – 7.21 (m, 5H), 7.09 – 6.97 (m, 2H), 4.72 (dd, *J* = 7.2, 5.2 Hz, 1H), 4.23 (dd, *J* = 13.6, 5.2 Hz, 1H), 3.92 (dd, *J* = 13.6, 7.2 Hz, 1H), 3.87 (s, 3H). The spectra were in agreement with reported data.[2]

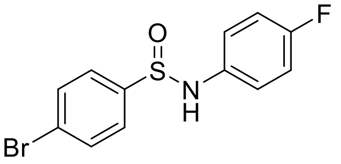

4-bromo-N-(4-fluorophenyl)benzenesulfinamide (**1h**). Prepared according to the general procedure A using 4-bromothiophenol (189 mg, 1 mmol). Purification by column chromatography (from 0.5% to 1% of MeOH in CH_2Cl_2) afforded the product as a white solid (121 mg, 39%).^{1}H NMR (400 MHz, $(CD_3)_2SO$) δ 9.34 (s, 1H), 7.82 – 7.75 (m, 2H), 7.68 – 7.61 (m, 2H), 7.14 – 7.01 (m, 4H). ^{19}F NMR (376 MHz, $(CD_3)_2SO$) δ -120.80 (tt, *J* = 8.3, 5.2 Hz). ^{13}C{^{1}H} NMR (101 MHz, $(CD_3)_2SO$) δ 158.5 (d, *J* = 238.6 Hz) 144.3, 138.0 (d, *J* = 2.5 Hz), 132.3, 128.3, 125.1, 120.9 (d, *J* = 8.0 Hz), 116.3 (d, *J* = 22.4 Hz). HRMS (ESI): *m/z* $[M+Na]^+$ calcd for $C_{12}H_9BrFNNaOS^+$ 335.9464; found 335.9463; IR (ν_{max}/cm^{-1}) 3080, 1504, 1466, 1051, 1005.

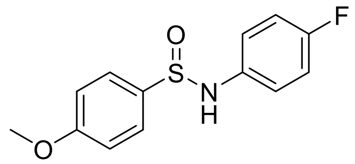

N-(4-fluorophenyl)-4-methoxybenzenesulfinamide (**2h**). Prepared according to the general procedure A using 4-methoxythiophenol

(140 mg, 1 mmol). Purification by column chromatography (25% of aceton in cyclohexane) and preparative HPLC afforded the product as a white solid (85mg, 32%). ^{1}H NMR (400 MHz, $CDCl_3$) δ 7.72 – 7.64 (m, 2H), 7.09 – 6.91 (m, 6H), 5.99 (s, 1H), 3.87 (s, 3H). ^{19}F NMR (376 MHz, $CDCl_3$) δ -118.89 (tt, J = 8.2, 4.7 Hz). ^{13}C{^{1}H} NMR (101 MHz, $CDCl_3$) δ 162.2, 159.62 (d, *J* = 242.9 Hz), 136.2 (d, *J* = 2.7 Hz), 135.4, 127.3, 122.0 (d, *J* = 8.1 Hz), 116.1 (d, *J* = 22.7 Hz), 114.5, 55.6. HRMS (ESI): *m/z* $[M+Na]^+$ calcd for $C_{13}H_{12}FNNaO_2S^+$ 288.0465; found 288.0466 IR (ν_{max}/cm^{-1}) 3178, 1589, 1248, 1090, 1051, 1026.

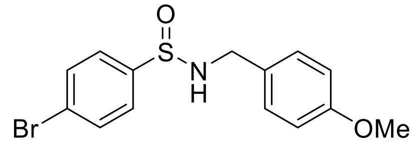

4-bromo-N-(4-methoxybenzyl)benzenesulfinamide (**1j**).[2] Prepared according to the general procedure A using 4-bromobenzenethiol (189 mg, 1 mmol). Purification by column chromatography (50 to 100 % of EtOAc in cyclohexane) afforded the product as a white solid (282 mg, 83%). ^{1}H NMR (400 MHz, $CDCl_3$) δ 7.68 – 7.59 (m, 4H), 7.20 – 7.14 (m, 2H), 6.88 – 6.81 (m, 2H), 4.55 – 4.40 (m, 1H), 4.17 (dd, *J* = 13.2, 4.8 Hz, 1H), 3.84 – 3.74 (m, 4H); The spectra were in agreement with reported data.[2]

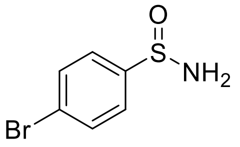

4-bromobenzenesulfinamide (**1k**).[6] Prepared according to the modified general procedure A using 4-bromothiophenol (189 mg, 1 mmol). Instead of an amine solution, a commercial 1M solution of LiHMDS was used (3 mL, 3 mmol) Purification by column chromatography (from 50% to 100% of EtOAc in cyclohexane) afforded the product as an off-white solid (100 mg, 46%).^{1}H NMR (400 MHz, $CDCl_3$) δ 7.70 – 7.57 (m, 4H), 4.34 (s, 2H). The spectra are in agreement with reported data.[6]

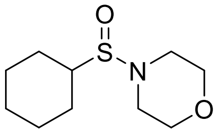

4-(cyclohexylsulfinyl)morpholine (**3a**).[7] Prepared according to the general procedure A using cyclohexanethiol (116 mg, 1 mmol). Purification by column chromatography (from 50% to 100% of EtOAc in cyclohexane) afforded the product as a white solid (145 mg, 67%). ^{1}H NMR (600 MHz, $CDCl_3$) δ 3.82 – 3.72 (m, 4H), 3.21 – 3.14 (m, 2H), 3.14 – 3.08 (m, 2H), 2.75 – 2.66 (m, 1H), 2.16 – 2.07 (m, 1H), 1.85 – 1.77 (m, 2H), 1.72 – 1.63 (m, 1H), 1.46 – 1.36 (m, 1H), 1.36 – 1.16 (m, 4H). The spectra were in agreement with reported data.[7]

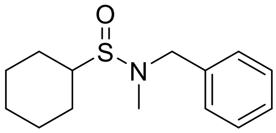

N-benzyl-N-methylcyclohexanesulfinamide (**3b**).[8] Prepared according to the general procedure A using cyclohexanethiol (116 mg, 1 mmol). Purification by column chromatography (50% of EtOAc in cyclohexane) afforded the product as an yellowish oil (213 mg, 85%).^{1}H NMR (400 MHz, $CDCl_3$) δ 7.39 – 7.27 (m, 5H), 4.26 (d, *J* = 14.3 Hz, 1H), 4.20 (d, *J* = 14.3 Hz, 1H), 2.75 – 2.65 (m, 1H), 2.60 (s, 3H), 2.20 – 2.10 (m, 1H), 1.95 – 1.78 (m, 3H), 1.72 – 1.65 (m, 1H), 1.52 – 1.39 (m, 1H), 1.39 – 1.18 (m, 4H). The spectra were in agreement with reported data [8]

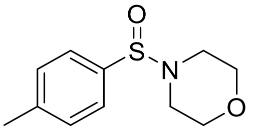

4-(p-tolylsulfinyl)morpholine (**4a**).[2] Prepared according to the general procedure B using TsCl (155 mg, 0.81 mmol). Purification by column chromatography (30 to 100 % of EtOAc in cyclohexane) afforded the product as a white solid (132 mg, 74 %). ^{1}H NMR (400 MHz, $CDCl_3$) δ 7.54 – 7.42 (m, 1H), 7.31 – 7.17 (m, 1H), 3.71 – 3.55 (m, 2H), 3.12 – 3.02 (m, 1H), 2.93 – 2.83 (m, 1H), 2.34 (s, 2H). The spectra were in agreement with reported data.[2]

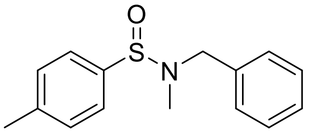

N-benzyl-N,4-dimethylbenzenesulfinamide (**4b**).[4] Prepared according to the general procedure A using 4-methylthiophenol (125 mg, 1 mmol). Purification by column chromatography (25% of EtOAc in cyclohexane) afforded the product as a yellowish oil (201 mg, 78%).^{1}H NMR (400 MHz, $CDCl_3$) δ 7.66 – 7.57 (m, 2H), 7.38 – 7.24 (m, 7H), 4.27 (d, *J* = 14.1 Hz, 1H), 4.13 (d, *J* = 14.1 Hz, 1H), 2.48 (s, 3H), 2.42 (s, 3H). The spectra were in agreement with reported data.[4]

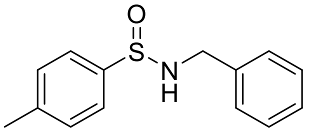

N-benzyl-4-methylbenzenesulfinamide (**4g**).[2] Prepared according to the general procedure B using TsCl (155 mg, 0.81 mmol). Purification by column chromatography (30 % of EtOAc in cyclohexane) afforded the product as a yellowish oil, which solidified on standing to a yellowish solid (160 mg, 80 %). ^{1}H NMR (400 MHz, $CDCl_3$) δ 7.69 – 7.59 (m, 2H), 7.36 – 7.21 (m, 7H), 4.46 – 4.31 (m, 1H), 4.23 (dd, *J* = 13.5, 5.2 Hz, 1H), 3.89 (dd, *J* = 13.5, 7.2 Hz, 1H), 2.41 (s, 3H). The spectra were in agreement with reported data.[2]

4-methyl-N-phenylbenzenesulfinamide (**4i**).[2] Prepared according to the general procedure B using TsCl (155 mg, 0.81 mmol, 1.0 eq). Purification by column chromatography (30 % of EtOAc in cyclohexane) afforded the product as a yellowish solid (117 mg, 62 %). ^{1}H NMR (400 MHz, $CDCl_3$) δ 7.71 – 7.61 (m, 2H), 7.37 – 7.21 (m, 4H), 7.14 – 6.99 (m, 3H), 6.23 (s, 1H), 2.43 (s, 3H); The spectra were in agreement with reported data.[2]

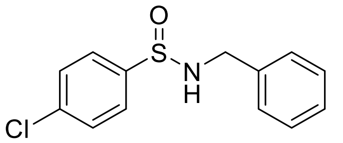

N-benzyl-4-chlorobenzenesulfinamide (**6g**).[2] Prepared according to the general procedure B using 4-chlorobenzenesulfonyl chloride (172 mg, 0.81 mmol). Purification by column chromatography (30 % of EtOAc in cyclohexane) afforded the product as a yellowish oil, which solidified on standing to a yellowish solid (143 mg, 66 %). ^{1}H NMR (400 MHz, $CDCl_3$) δ 7.52 – 7.41 (m, 2H), 7.31 – 7.21 (m, 2H), 7.15 – 6.97 (m, 5H), 4.58 (dd, *J* = 7.1, 5.0 Hz, 1H), 3.99 (dd, *J* = 13.6, 5.1 Hz, 1H), 3.64 (dd, *J* = 13.6, 7.1 Hz, 1H); The spectra were in agreement with reported data.[2]

3. General Procedure for the Preparation of Sulfonamides

To a solution of a sulfonyl chloride (0.5 mmol) in CH_2Cl_2 (5 mL), an amine (0.75 mmol, 1.5 eq.) was added in one portion at rt followed by a dropwise addition of Et_3N (210 µL, 1.5 mmol, 1.5 eq.).

The reaction mixture was stirred for 5-30 min at rt until all the starting material was consumed. The reaction mixture was diluted with CH_2Cl_2 (25 mL) and washed sequentially with saturated aq. NH_4Cl solution (10 mL), saturated aq. $NaHCO_3$ solution (10 mL), and brine (10 mL). The organic phase was dried over anhydrous $MgSO_4$ and concentrated under reduced pressure. The resulting sulfonamides were used without further purification unless otherwise stated.

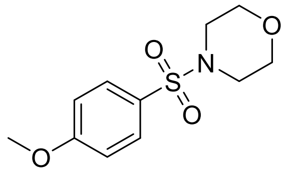

4-((4-methoxyphenyl)sulfonyl)morpholine (**5a**).[9] Prepared according to the general procedure for sulfonamides. The product was obtained as a white solid. (120 mg, 93%). ^{1}H NMR (300 MHz, $CDCl_3$) δ 7.75 – 7.65 (m, 2H), 7.06 – 6.96 (m, 2H), 3.89 (s, 3H), 3.79 – 3.69 (m, 4H), 3.03 – 2.93 (m, 4H). The spectra were in agreement with reported data.[9]

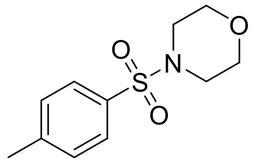

4-((4-methylphenyl)sulfonyl)morpholine (**5b**).[10] Prepared according to the general procedure for sulfonamides. The product was obtained as a white solid (93 mg, 77%). ^{1}H NMR (400 MHz, $CDCl_3$) δ 7.69 – 7.59 (m, 2H), 7.41 – 7.31 (m, 2H), 3.81 – 3.66 (m, 4H), 3.05 – 2.93 (m, 4H), 2.45 (s, 3H). The spectra were in agreement with reported data.[10]

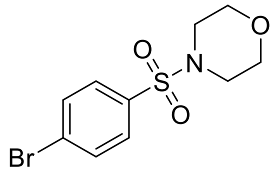

4-((4-bromophenyl)sulfonyl)morpholine (**5c**).[11] Prepared according to the general procedure for sulfonamides. The product was obtained as a white solid (144 mg, 94%). ^{1}H NMR (400 MHz, $CDCl_3$) δ 7.75 – 7.67 (m, 2H), 7.67 – 7.57 (m, 2H), 3.83 – 3.64 (m, 4H), 3.11 – 2.90 (m, 4H). The spectra were in agreement with reported data.[11]

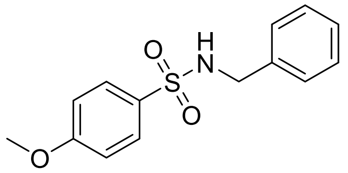

N-benzyl-4-methoxybenzenesulfonamide (**5d**).[12] Prepared according to the general procedure for sulfonamides. The crude product was purified by column chromatography (from 25% to 50% of EtOAc in cyclohexane) affording the product as a white solid (127 mg, 92%). ^{1}H NMR (400 MHz, $CDCl_3$) δ 7.86 – 7.77 (m, 2H), 7.33 – 7.24 (m, 3H), 7.23 – 7.17 (m, 2H), 7.02 – 6.94 (m, 2H), 4.58 (t, *J* = 6.1 Hz, 1H), 4.12 (d, *J* = 6.2 Hz, 2H), 3.88 (s, 3H). The spectra were in agreement with reported data.[12]

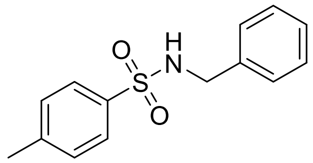

N-benzyl-4-methylbenzenesulfonamide (**5e**).[13] Prepared according to the general procedure for sulfonamides. The crude product was purified by column chromatography (from 25% to 50% of EtOAc in cyclohexane) affording the product as a white solid (110 mg, 84%). ^{1}H NMR (400 MHz, $CDCl_3$) δ 7.83 – 7.70 (m, 2H), 7.36 – 7.24 (m, 6H), 7.22 – 7.16 (m, 2H),

4.68 (t, *J* = 6.2 Hz, 1H), 4.12 (d, *J* = 6.2 Hz, 2H), 2.44 (s, 3H) The spectra were in agreement with reported data.[13]

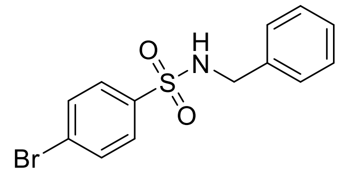

N-benzyl-4-bromobenzenesulfonamide (**5f**).[12] Prepared according to the general procedure for sulfonamides. The crude product was purified by column chromatography (from 25% to 50% of EtOAc in cyclohexane) affording the product as a white solid (119 mg, 73%). ^{1}H NMR (400 MHz, $CDCl_3$) δ 7.76 – 7.67 (m, 2H), 7.67 – 7.60 (m, 2H), 7.33 – 7.26 (m, 3H), 7.22 – 7.14 (m, 2H), 4.70 (t, *J* = 6.1 Hz, 1H), 4.16 (d, *J* = 6.1 Hz, 2H). The spectra were in agreement with reported data.[12]

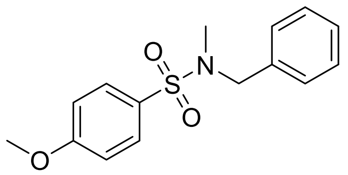

N-benzyl-4-methoxy-N-methylbenzenesulfonamide (**5g**).[14] Prepared according to the general procedure for sulfonamides. The crude product was purified by column chromatography (from 25% to 33% of EtOAc in cyclohexane) affording the product as a white solid (135 mg, 93%). ^{1}H NMR (400 MHz, $CDCl_3$) δ 7.82 – 7.74 (m, 2H), 7.37 – 7.27 (m, 5H), 7.07 – 6.99 (m, 2H), 4.12 (s, 2H), 3.89 (s, 3H), 2.58 (s, 3H). The spectra were in agreement with reported data.[14]

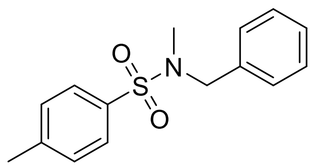

N-benzyl-N,4-dimethylbenzenesulfonamide (**5h**).[15] Prepared according to the general procedure for sulfonamides. The crude product was purified by column chromatography (from 25% to 33% of EtOAc in cyclohexane) affording the product as a white solid (129 mg, 94%). ^{1}H NMR (400 MHz, $CDCl_3$) δ 7.79 – 7.69 (m, 2H), 7.39 – 7.27 (m, 7H), 4.13 (s, 2H), 2.58 (s, 3H), 2.46 (s, 3H). The spectra were in agreement with reported data.[15]

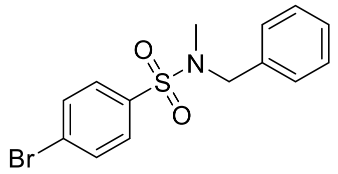

N-benzyl-4-bromo-N-methylbenzenesulfonamide (**5i**).[14] Prepared according to the general procedure for sulfonamides. The crude product was purified by column chromatography (from 25% to 33% of EtOAc in cyclohexane) affording the product as a white solid (123 mg, 73%). ^{1}H NMR (400 MHz, $CDCl_3$) δ 7.74 – 7.68 (m, 4H), 7.39 – 7.27 (m, 5H), 4.15 (s, 2H), 2.61 (s, 3H). The spectra were in agreement with reported data.[14]

4. General Procedure for the Crossover Reaction

250 mM stock solutions of individual sulfinamides were prepared in DMSO and dried with 4Å MS overnight. For the crossover reaction of two sulfinamides, 40 µL of the stock solution of each sulfinamide was placed into 1.5 mL glass vial with a screwing cap, followed by 19 µL of DMSO. The reaction was started by adding of 1 µL of freshly prepared 1M stock solution of trifluoroacetic acid (TFA) in DMSO (final concentration of sulfinamides 200 mM, final concentration of TFA 10 mM). The

reaction vial was filled with argon, parafilmed and incubated at 40 °C. For the crossover reaction of three sulfinamides, 40 µL of the stock solution of each sulfinamide was placed into 1.5 mL glass vial with a screwing cap, followed by 29 µL of DMSO. The reaction was started by adding of 1 µL of freshly prepared 1M stock solution of trifluoroacetic acid (TFA) in DMSO (final concentration of sulfinamides 200 mM, final concentration of TFA 6.6 mM). The progress of the reaction was monitored by analytical HPLC. Aliquots (10 µL) of the reactions for the HPLC analysis were diluted with 110 mM Et_3N in CH_3CN (190 µL) and analysed. Yields and molar ratio of sulfinamides were derived from calibration curves obtained by HPLC analysis of serial dilutions of individual sulfinamide stocks. HPLC data were integrated with MassHunter Workstation software and analysed in Excel. HPLC chromatograms were visualized using a custom Python script. Specific reaction times are mentioned in HPLC chromatogram data.

5. Reaction of Sulfinamide 1a with *N*-methyl-1-phenylmethanamine Under Crossover Condition

250 mM stock solutions of sulfinamide **1a** and *N*-methyl-1-phenylmethanamine were prepared in DMSO and dried with 4Å MS overnight. For the crossover reaction test, 40 µL of each of the stock solution sulfinamide and amine were placed into 1.5 mL glass vial with a screwing cap, followed by 19 µL or 12 µL of DMSO. The reaction was started by adding of 1 µL or 8 µL of freshly prepared 1M stock solution of TFA in DMSO (final concentration of sulfinamide 100 mM and amine 100 mM, final concentration of TFA 10 mM and 80 mM resp. The reaction vial was filled with argon, parafilmed and incubated at 40 °C. The progress of the reaction was monitored by analytical HPLC. Aliquots (10 µL) of the reactions for the HPLC analysis were diluted with 110 mM Et_3N in CH_3CN (190 µL) and analysed. HPLC analysis indicated no significant production of complementary sulfinamide **1b** after 6 h (trace amount was detected using MS).

6. Oxidation of a Mini-Library of Sulfinamides to Sulfonamides

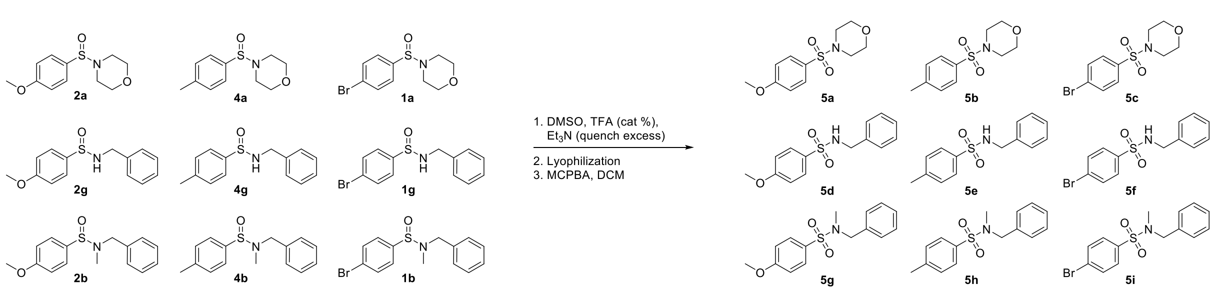

Microscale oxidation of a mini-library of sulfinamides to sulfonamides was performed under unoptimized conditions as follows. 50 µl of each sulfinamide **1a**, **1b**, **1g**, **2a**, **2b**, **2g**, **4a**, **4b**, **4g** (250 mM in DMSO) was added to 1.5 mL Eppendorf vial and further diluted with 175 µL of DMSO providing 180 mM solution of total sulfinamides (20 mM each). 100 µL aliquot (18 µmol of total sulfinamides) was transferred to new 1.5 mL eppendorf vial and 1 µL of trifluoroacetic acid (1 M in DMSO) was added to simulate the crossover conditions. Then, 5 µL (36 µmol) of Et_3N were added and the mixture was lyophilized. The obtained oily residue was dissolved in CH_2Cl_2 (500 µL), mCPBA

(<77%, 9 mg, 40 µmol) was added in one portion and the reaction mixture was agitated for 1 hour at rt. Then, 10 µL aliquot of the reaction mixture was diluted with Et_3N solution in CH_3CN (110 mM, 190 µL) and subjected to HPLC analysis. Total yield of sulfonamides was >70%. The yield was derived from calibration curves obtained by HPLC analysis of serial dilutions of an equimolar mixture of sulfonamide standards. Interference of mCPBA/mCBA in the chromatograms prevented more precise calculation of the yield.

7. The Stability Test of Sulfinamide and Sulfonamide Libraries in Aqueous Solutions

50 µl of each sulfinamide **1a**, **1b**, **1g**, **2a**, **2b**, **2g**, **4a**, **4b**, **4g** (250 mM in DMSO) or sulfonamide **5a-5i** (250 mM in DMSO) was added to 1.5 mL Eppendorf vial and further diluted with 175 µL of DMSO providing 180 mM solution of total sulfinamides or sulfonamides (20 mM each). 5 µl of such a mixture of sulfinamides or sulfonamide was added to 1.5 mL Eppendorf vial followed by a solution of water in CH_3CN (1:1; 44 µL). Then, 1 µL of TFA (1 M in DMSO) was added and the reaction mixture was incubated for 1 hour at rt. For neutral aqueous condition experiments, 1 µL of DMSO was used instead of TFA. After 1h, 10 µL aliquots of the reaction mixtures were diluted with Et_3N solution in CH_3CN (110 mM, 190 µL) and subjected to HPLC analysis.

8. Preliminary Solvent and Acid Screening

Equimolar amount of two sulfinamides **1j** (25 mg, 0.075 mmol) and **6g** (20 mg, 0.075 mmol) were dissolved in a mixture of CH_2Cl_2 (800 µL) and MeOH (80 µL). Then, aliquots of sulfinamide solutions (55 µL, 4.6 µmol of each sulfinamide) were placed into glass vials and were evaporated under reduced pressure. Different solvents (50 µL, 92mM solution, final concentration 88mM) were added to the obtained residues in vials and mixed to get a solution or a suspension. Reactions were initiated by the addition of glacial AcOH (2.5 µL, 5% vv, aprox. 10 eq.) to the reaction mixtures and stirred at rt for 1h. The progress of the reaction was monitored by analytical HPLC-MS after dillution with CH_3CN. Extracted mass (*m/z* $[M+H^+]$) chromatograms belonging to each sulfinamides (for the four products **1j**, **6g**, **1g**, **6j**) were integrated and analyzed.

Similarly, equimolar mixture of two sulfinamides **1j** (25 mg, 0.075 mmol) and **6g** (20 mg, 0.075 mmol were dissolved in CH_3CN (100 µL, 46 mM for each sulfinamide) and stock solutions of different acids in CH_3CN (1 µL, final concentration 4.6 mM) were added and the reaction mixtures were incubated at rt without mixing. The progress of the reaction was monitored by analytical HPLC-MS after dillution with CH_3CN. Extracted mass (*m/z* $[M+H^+]$) chromatograms belonging to each sulfinamides (for the four products **1j**, **6g**, **1g**, **6j**) were integrated and analyzed.

9. Stability Test of *N*-aromatic Sulfinamide 4i Under Crossover Conditions

A solution of TFA in CH_3CN (5 µL, 184 mM, final concentration 4.6 mM) was added to a solution of sulfinamide **4i** in CH_3CN (190 µL, 48 mM, final concentration 46 mM) followed by 5 µL of CH_3CN to the total volume of 200 µL. A reaction without an acid was carried out as a control. The reaction vial was incubated at 30 °C. The progress of the reaction was monitored by analytical HPLC-MS. Aliquots (10 µL) of the reactions were diluted with CH_3CN (990 µL) and subjected to HPLC-MS analysis. Extracted mass chromatograms of *m/z* $[M+H^+]$ = 232 were analyzed.

10. References

(1) Smith, C. R. Activated Zinc Dust. *Synlett* **2009**, *2009* (9), 1522–1523. https://doi.org/10.1055/s-0029-1217181.

(2) Jabczun, M.; Nosek, V.; Míšek, J. Complementary Strategies for Synthesis of Sulfinamides from Sulfur-Based Feedstock. *Org. Biomol. Chem.* **2023**, *21* (14), 2950–2954. https://doi.org/10.1039/D3OB00050H.

(3) Wang, Q.; Tang, X.-Y.; Shi, M. Metal-Free Cross-Coupling of Arylboronic Acids and Derivatives with DAST-Type Reagents for Direct Access to Diverse Aromatic Sulfinamides and Sulfonamides. *Angewandte Chemie International Edition* **2016**, *55* (36), 10811–10815. https://doi.org/10.1002/anie.201605066.

(4) Yu, H.; Li, Z.; Bolm, C. Copper-Catalyzed Transsulfinamidation of Sulfinamides as a Key Step in the Preparation of Sulfonamides and Sulfonimidamides. *Angewandte Chemie International Edition* **2018**, *57* (47), 15602–15605. https://doi.org/10.1002/anie.201810548.

(5) Izzo, F.; Schäfer, M.; Stockman, R.; Lücking, U. A New, Practical One-Pot Synthesis of Unprotected Sulfonimidamides by Transfer of Electrophilic NH to Sulfinamides. *Chemistry – A European Journal* **2017**, *23* (60), 15189–15193. https://doi.org/10.1002/chem.201703272.

(6) Chatterjee, S.; Makai, S.; Morandi, B. Hydroxylamine-Derived Reagent as a Dual Oxidant and Amino Group Donor for the Iron-Catalyzed Preparation of Unprotected Sulfinamides from Thiols. *Angewandte Chemie International Edition* **2021**, *60* (2), 758–765. https://doi.org/10.1002/anie.202011138.

(7) Lo, P. K. T.; Oliver, G. A.; Willis, M. C. Sulfinamide Synthesis Using Organometallic Reagents, DABSO, and Amines. *J. Org. Chem.* **2020**, *85* (9), 5753–5760. https://doi.org/10.1021/acs.joc.0c00334.

(8) Dai, Q.; Zhang, J. Direct Synthesis of Sulfinamides by the Copper-Catalyzed Electrophilic Amidation of Sulfenate Anions. *Advanced Synthesis & Catalysis* **2018**, *360* (6), 1123–1127. https://doi.org/10.1002/adsc.201701510.

(9) DeBergh, J. R.; Niljianskul, N.; Buchwald, S. L. Synthesis of Aryl Sulfonamides via Palladium-Catalyzed Chlorosulfonylation of Arylboronic Acids. *J. Am. Chem. Soc.* **2013**, *135* (29), 10638–10641. https://doi.org/10.1021/ja405949a.

(10) Jiang, Y.; Wang, Q.-Q.; Liang, S.; Hu, L.-M.; Little, R. D.; Zeng, C.-C. Electrochemical Oxidative Amination of Sodium Sulfinates: Synthesis of Sulfonamides Mediated by NH4I as a Redox Catalyst. *J. Org. Chem.* **2016**, *81* (11), 4713–4719. https://doi.org/10.1021/acs.joc.6b00615.

(11) Huang, H.-M.; Bellotti, P.; Erchinger, J. E.; Paulisch, T. O.; Glorius, F. Radical Carbonyl Umpolung Arylation via Dual Nickel Catalysis. *J. Am. Chem. Soc.* **2022**, *144* (4), 1899–1909. https://doi.org/10.1021/jacs.1c12199.

(12) Reed-Berendt, B. G.; Morrill, L. C. Manganese-Catalyzed N-Alkylation of Sulfonamides Using Alcohols. *J. Org. Chem.* **2019**, *84* (6), 3715–3724. https://doi.org/10.1021/acs.joc.9b00203.

(13) Xu, Q.; Li, Q.; Zhu, X.; Chen, J. Green and Scalable Aldehyde-Catalyzed Transition Metal-Free Dehydrative N-Alkylation of Amides and Amines with Alcohols. *Advanced Synthesis & Catalysis* **2013**, *355* (1), 73–80. https://doi.org/10.1002/adsc.201200881.

(14) Powell, D. A.; Fan, H. Copper-Catalyzed Amination of Primary Benzylic C−H Bonds with Primary and Secondary Sulfonamides. *J. Org. Chem.* **2010**, *75* (8), 2726–2729. https://doi.org/10.1021/jo100197r.

(15) Powell, D. A.; Pelletier, G. Copper Triflate/t-BuOOAc-Catalyzed Amidation of Allylic and Benzylic Acetates with Sulfonamides. *Tetrahedron Letters* **2008**, *49* (16), 2495–2498. https://doi.org/10.1016/j.tetlet.2008.02.135.

11. HPLC Chromatograms

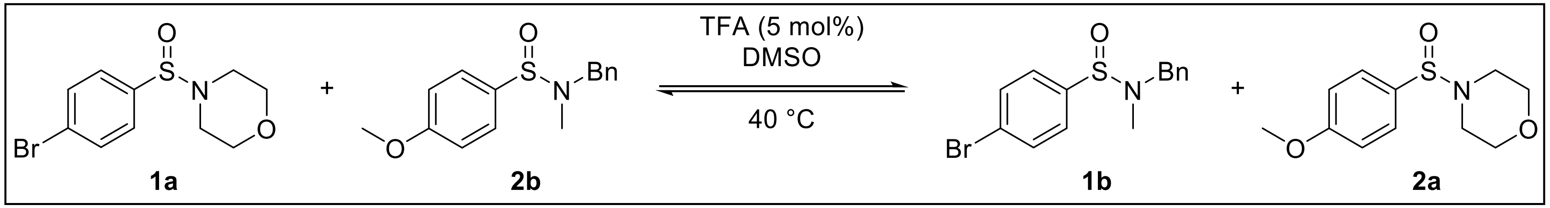

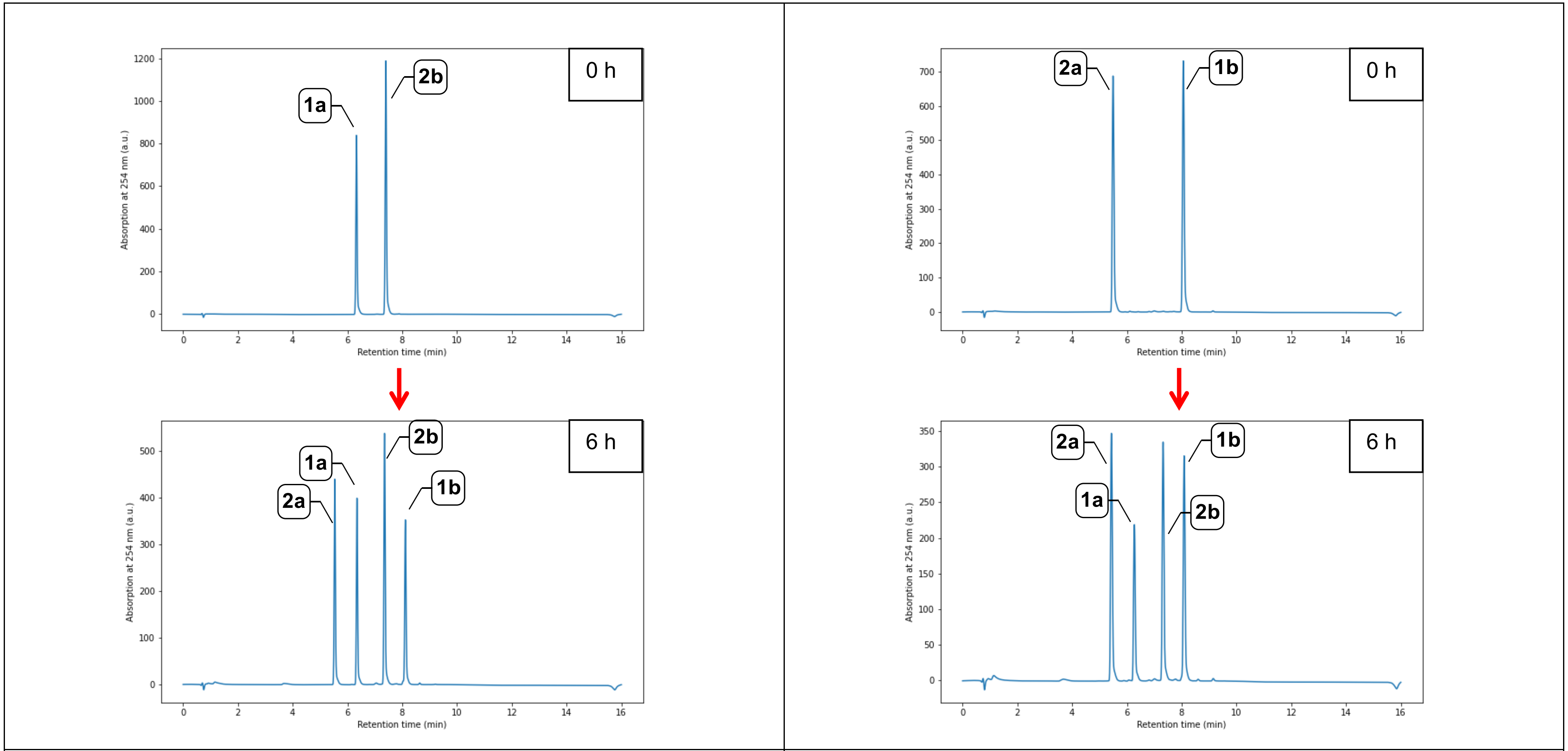

Total yield of sulfinamide products = **85%**<br>Molar ratio of products:<br>**1a** : **2b** : **1b** : **2a** = 1.1 : 1.0 : 0.9 : 1.0	Total yield of sulfinamide products = **86%**<br>Molar ratio of products:<br>**1a** : **2b** : **1b** : **2a** = 0.9 : 0.9 : 1.1 : 1.2

Figure S1. HPLC chromatograms of the crossover reaction of **1a**, **2b** and **2a**, **1b**.

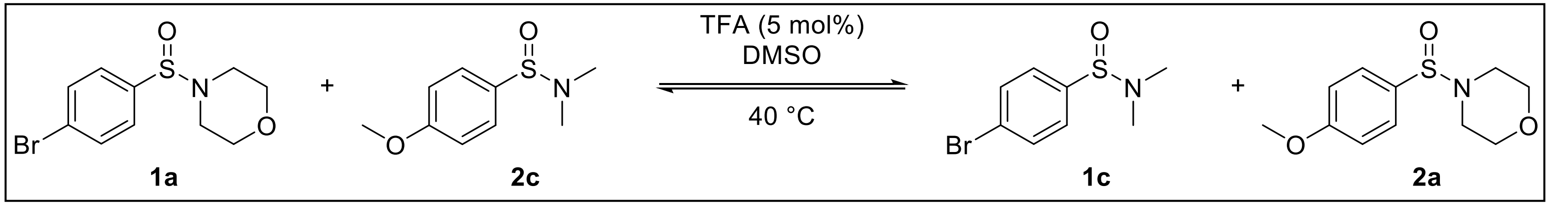

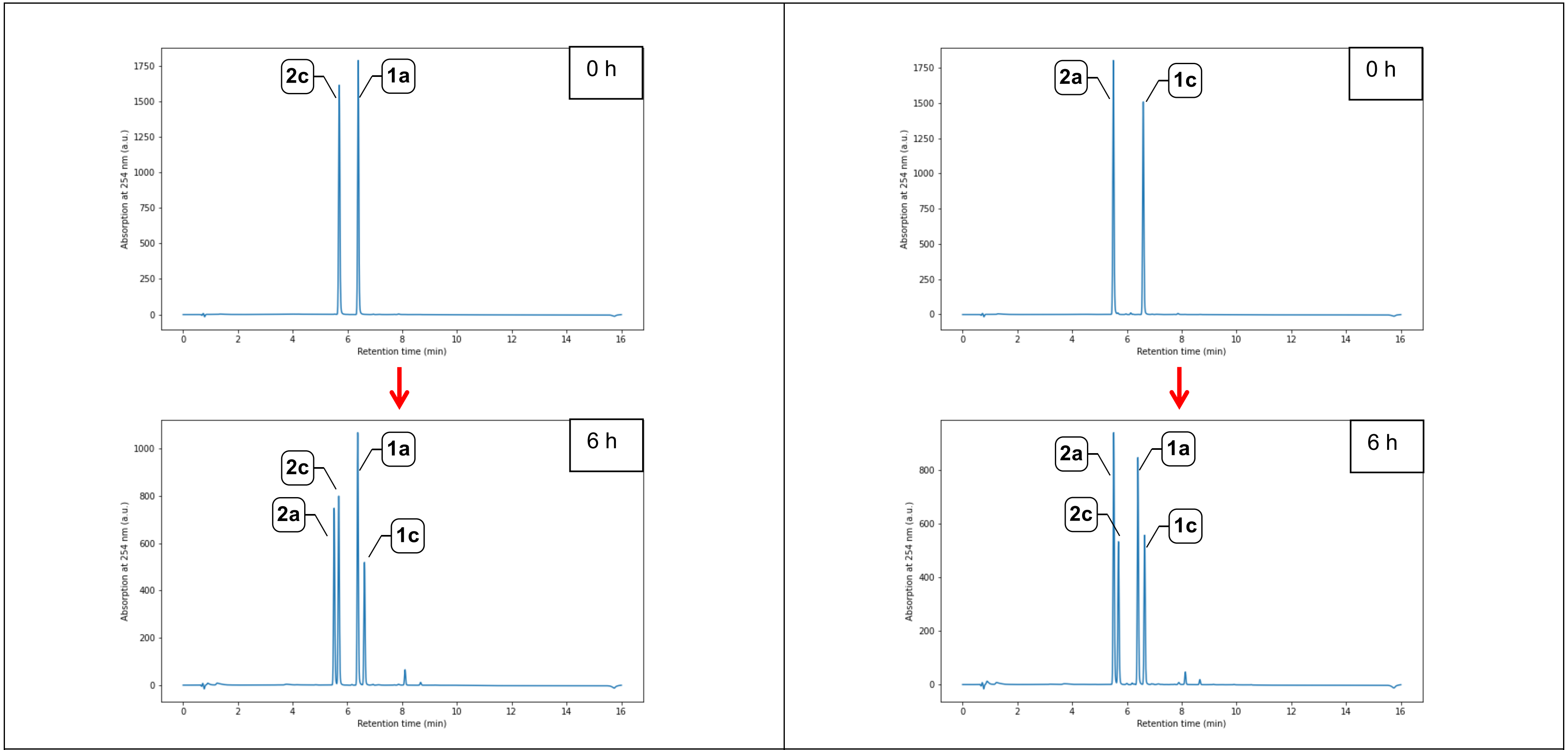

Total yield of sulfinamide products = **86%**<br>Molar ratio of products:<br>**1a** : **2c** : **1c** : **2a** = 1.5 : 1.1 : 0.8 : 0.9	Total yield of sulfinamide products = **88%**<br>Molar ratio of products:<br>**1a** : **2c** : **1c** : **2a** = 1.3 : 0.8 : 0.9 : 1.1

Figure S2. HPLC chromatograms of the crossover reaction of **1a**, **2c** and **2a**, **1c**.

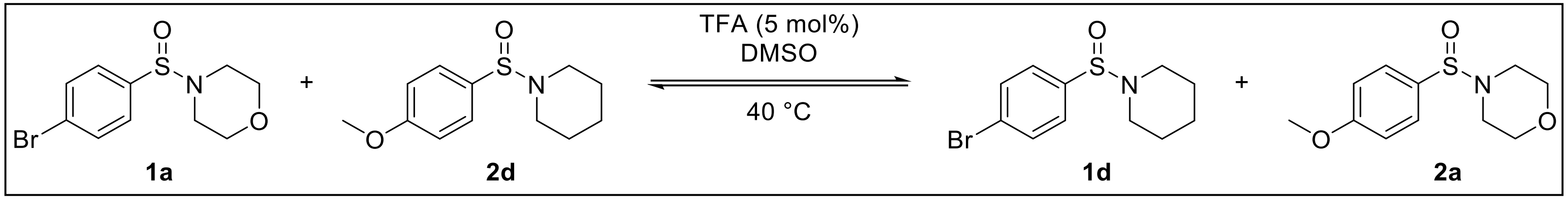

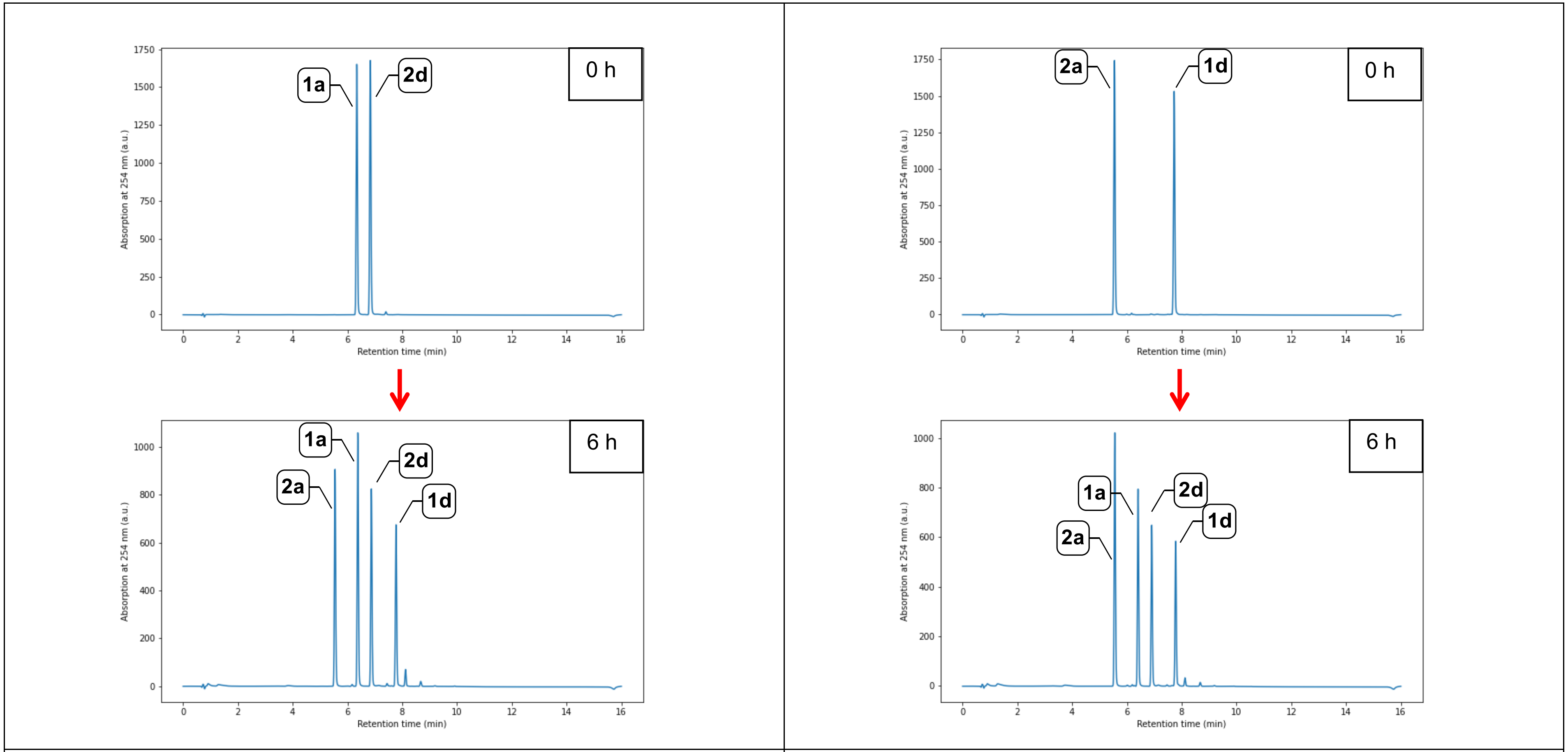

Total yield of sulfinamide products = **89%**<br>Molar ratio of products:<br>**1a** : **2d** : **1d** : **2a** = 1.4 : 1.0 : 1.0 : 1.0	Total yield of sulfinamide products = **86%**<br>Molar ratio of products:<br>**1a** : **2d** : **1d** : **2a** = 1.1 : 0.8 : 0.9 : 1.2

Figure S3. HPLC chromatograms of the crossover reaction of **1a**, **2d** and **2a**, **1d**.

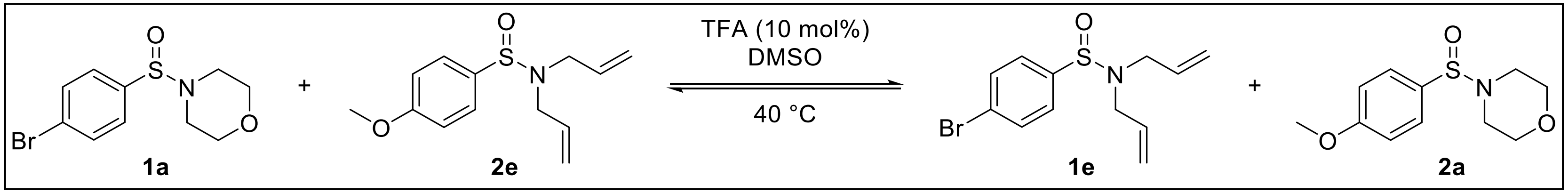

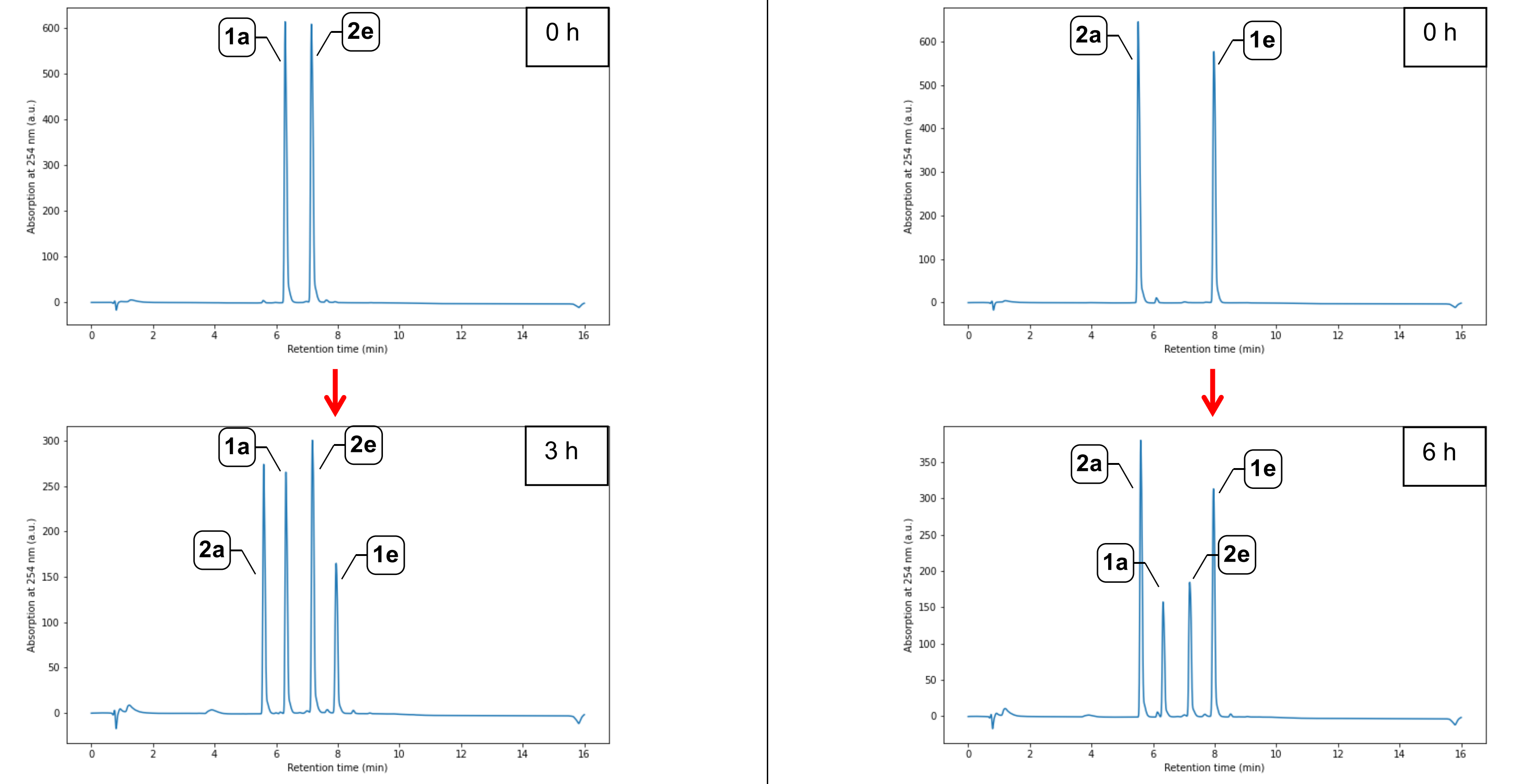

Total yield of sulfinamide products = **82%**
Molar ratio of products:
1a : **2e** : **1e** : **2a** = 1.2 : 1.0 : 0.7 : 1.0

Total yield of sulfinamide products = **82%**
Molar ratio of products:
1a : **2e** : **1e** : **2a** = 0.7 : 0.6 : 1.3 : 1.4

Figure S4. HPLC chromatograms of the crossover reaction of **1a**, **2e** and **2a**, **1e**.

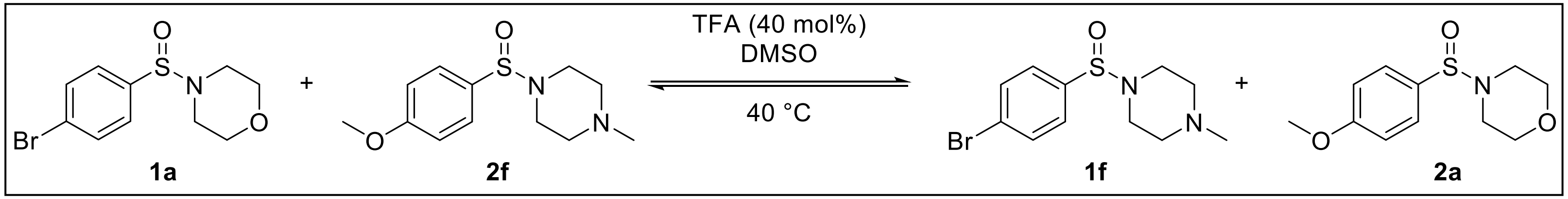

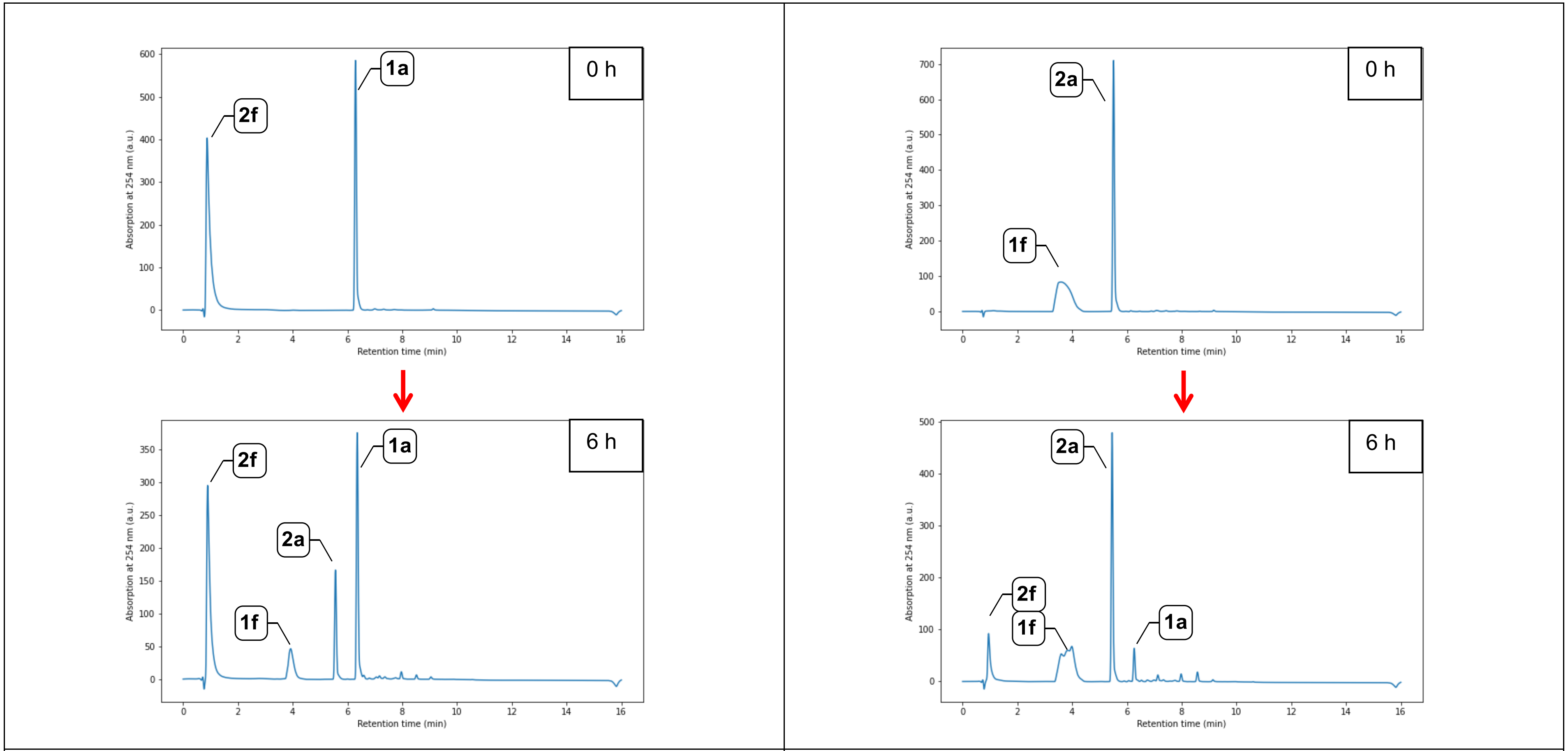

Total yield of sulfinamide products = **89**<br>Molar ratio of products:<br>**1a** : **2f** : **1f** : **2a** = 1.4 : 1.5 : 0.5 : 0.6	Total yield of sulfinamide products = **84%**<br>Molar ratio of products:<br>**1a** : **2f** : **1f** : **2a** = 0.3 : 0.4 : 1.9 : 1.6

Figure S5. HPLC chromatograms of the crossover reaction of **1a**, **2f** and **2a**, **1f**.

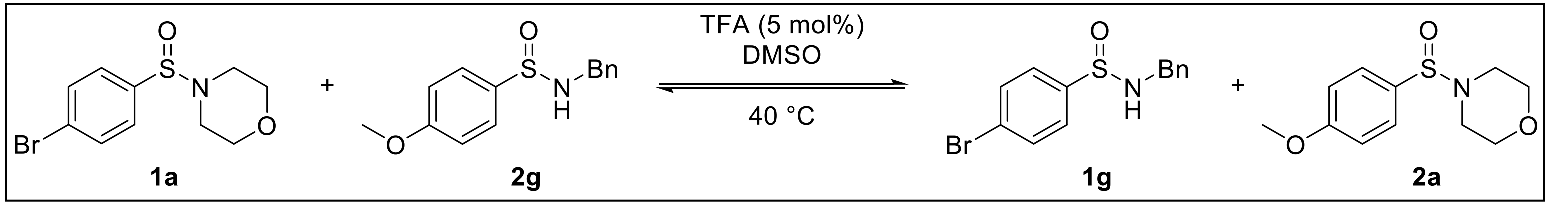

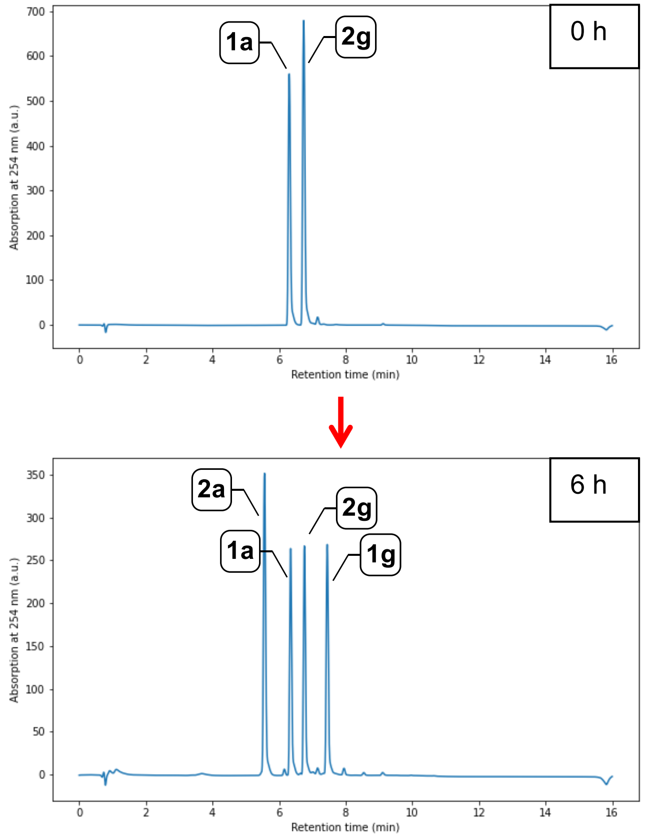

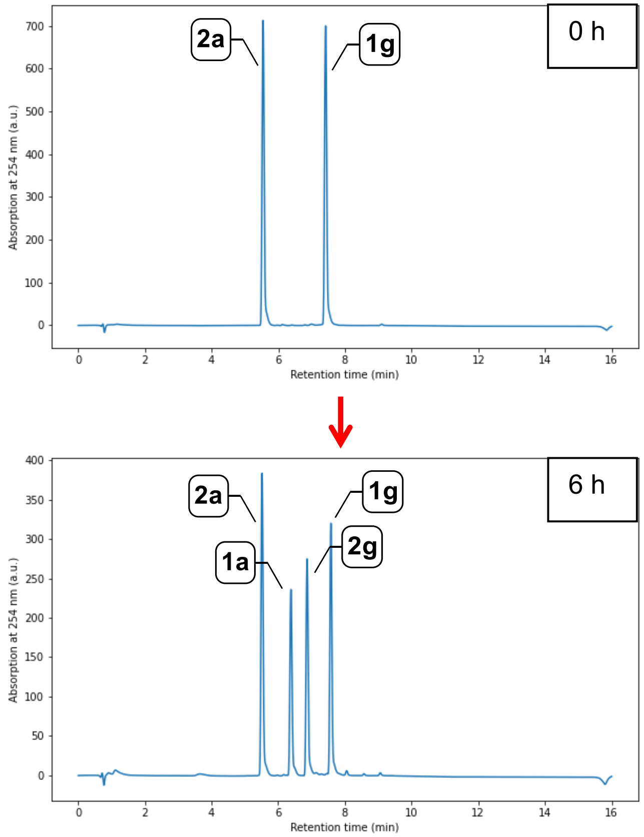

Total yield of sulfinamide products = **85%**
Molar ratio of products:
1a : **2g** : **1g** : **2a** = 1.1 : 0.9 : 0.9 : 1.2

Total yield of sulfinamide products = **87%**
Molar ratio of products:
1a : **2g** : **1g** : **2a** = 1.0 : 0.9 : 1.1 : 1.2

Figure S6. HPLC chromatograms of the crossover reaction of **1a**, **2g** and **2a**, **1g**.

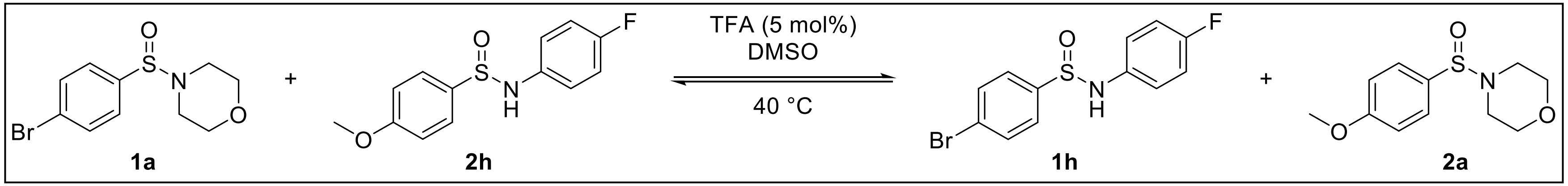

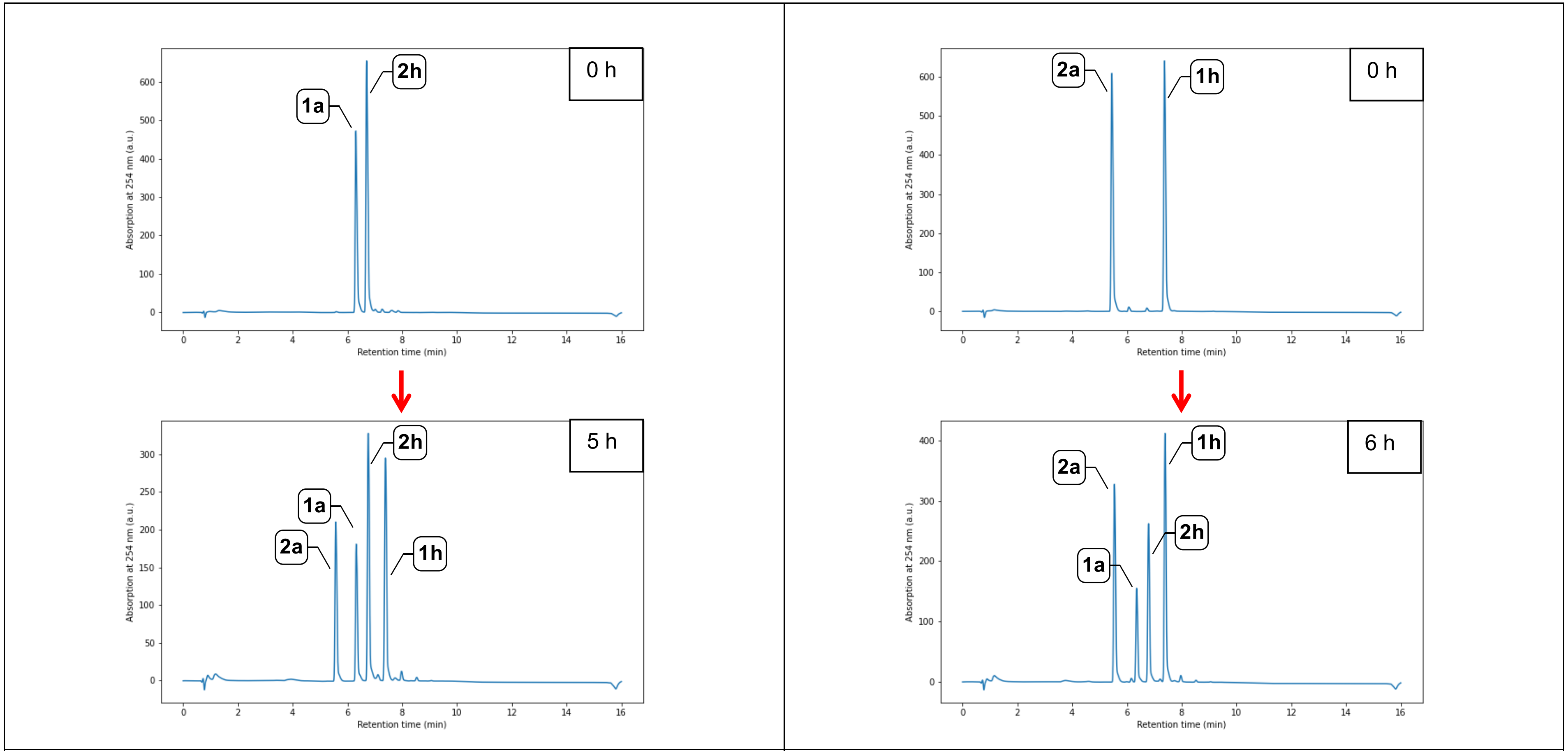

Total yield of sulfinamide products = **84%**<br>Molar ratio of products:<br>**1a** : **2h** : **1h** : **2a** = 1.0 : 1.1 : 1.0 : 0.9	Total yield of sulfinamide products = **91%**<br>Molar ratio of products:<br>**1a** : **2h** : **1h** : **2a** = 0.8 : 0.8 : 1.2 : 1.4

Figure S7. HPLC chromatograms of the crossover reaction of **1a**, **2h** and **2a**, **1h**.

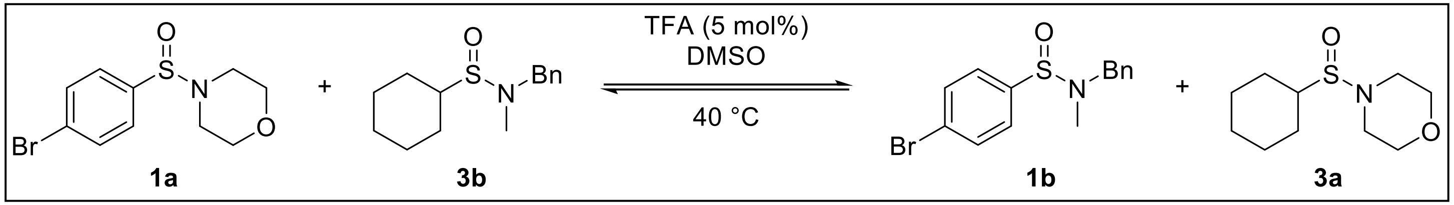

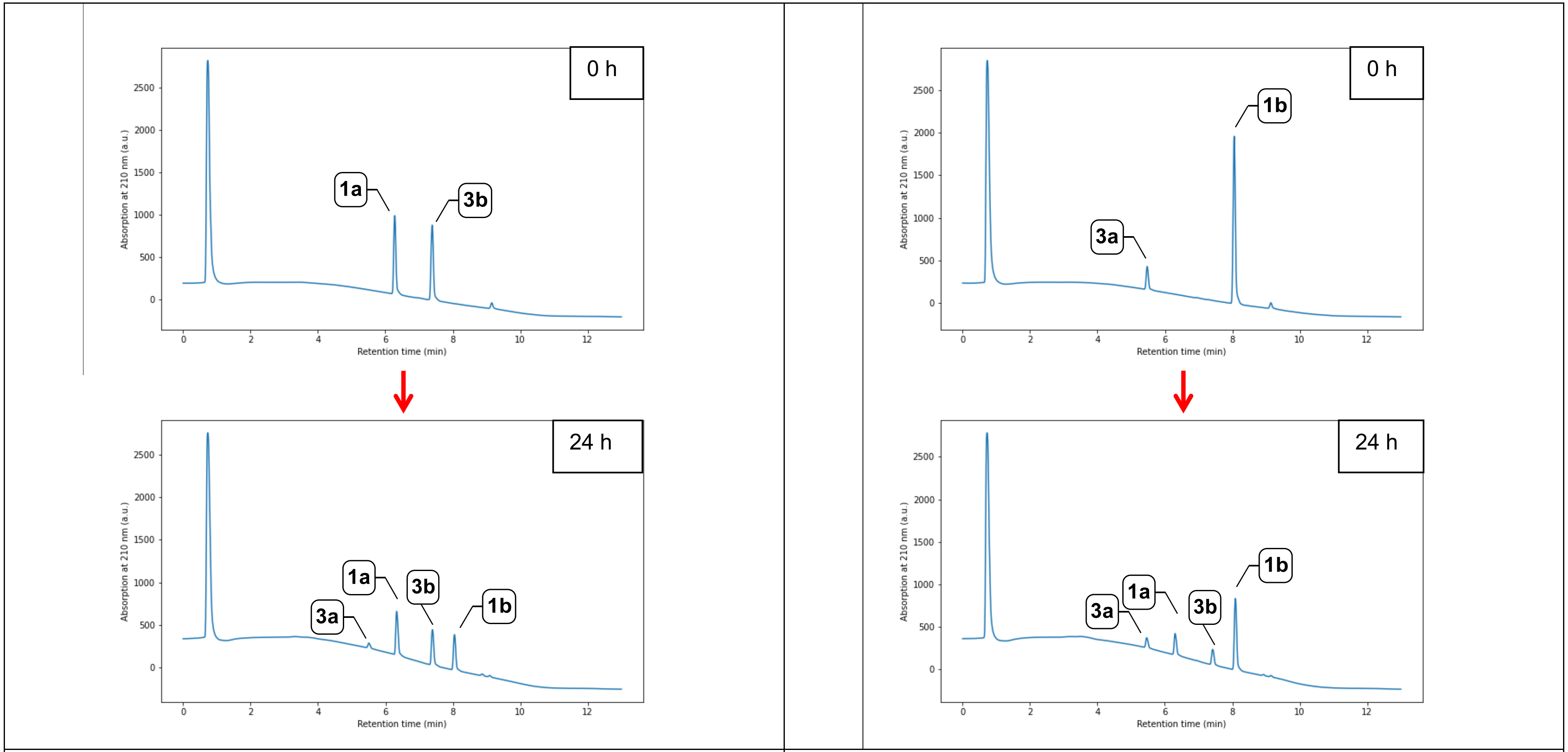

Total yield of sulfinamide products = **80%**<br>Molar ratio of products:<br>**1a** : **3b** : **1b** : **3a** = 1.7 : 1.2 : 0.7 : 0.8	Total yield of sulfinamide products = **68%**<br>Molar ratio of products:<br>**1a** : **3b** : **1b** : **3a** = 0.8 : 0.5 : 1.3 : 1.4

Figure S8. HPLC chromatograms of the crossover reaction of **1a**, **3b** and **3a**, **1b**.

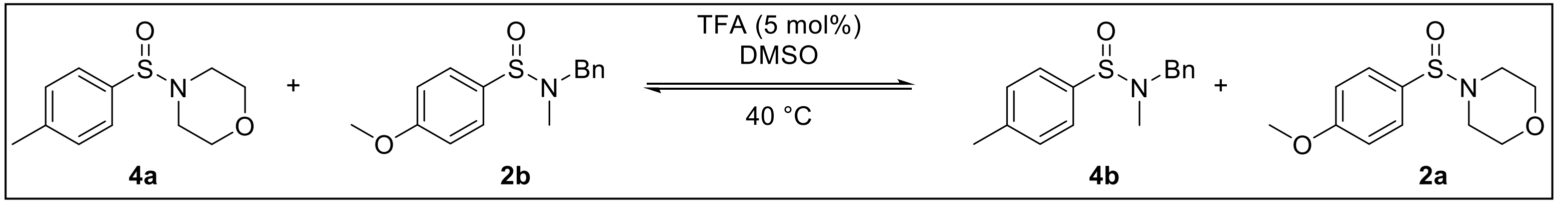

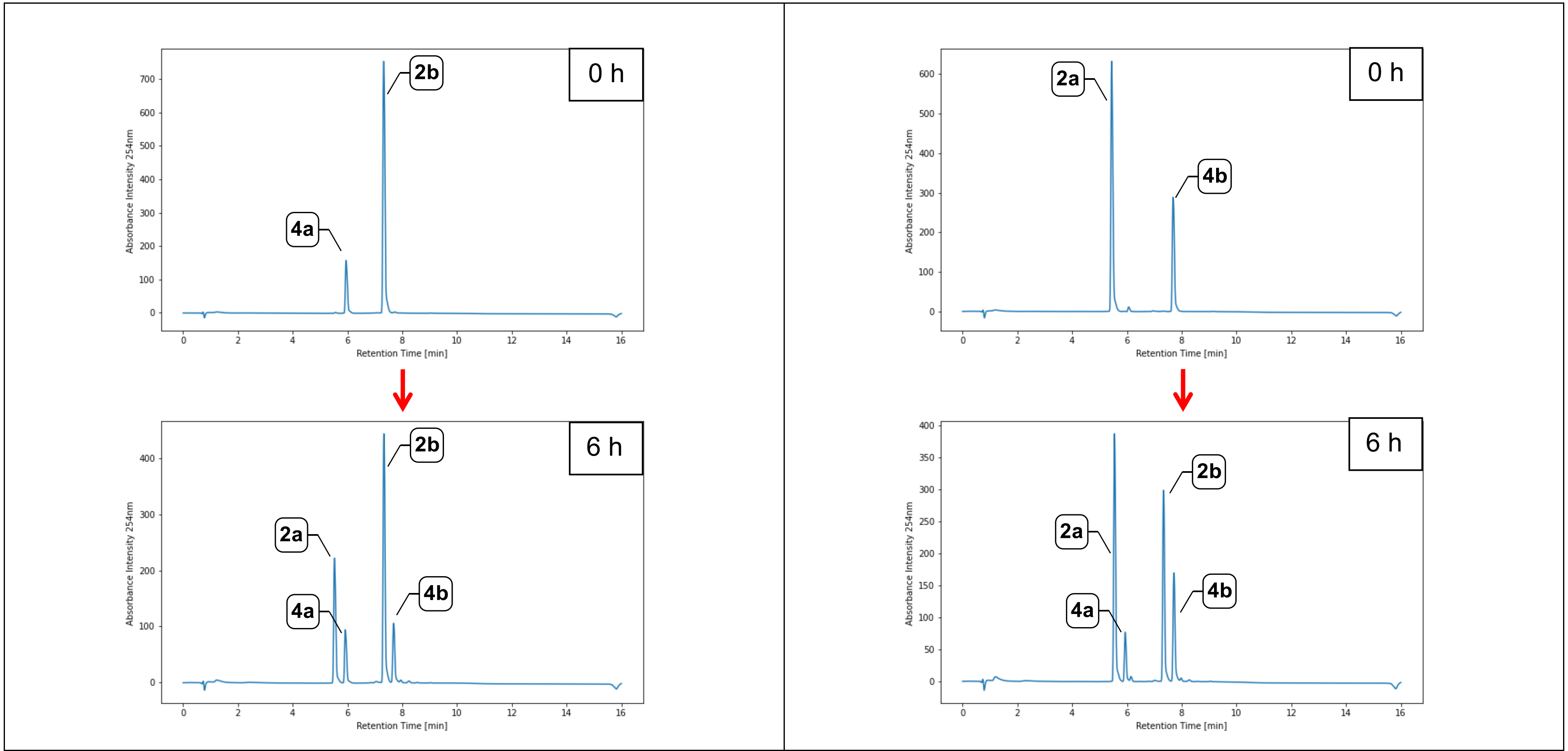

Total yield of sulfinamide products = **96%**. Molar ratio of products: **4a** : **2b** : **4b** : **2a** = 1.2 : 1.2 : 0.8 : 0.8	Total yield of sulfinamide products = **95%**. Molar ratio of products: **4a** : **2b** : **4b** : **2a** = 0.8 : 0.7 : 1.2 : 1.2

Figure S9. HPLC chromatograms of the crossover reaction of **4a**, **2b** and **4b**, **2a**.

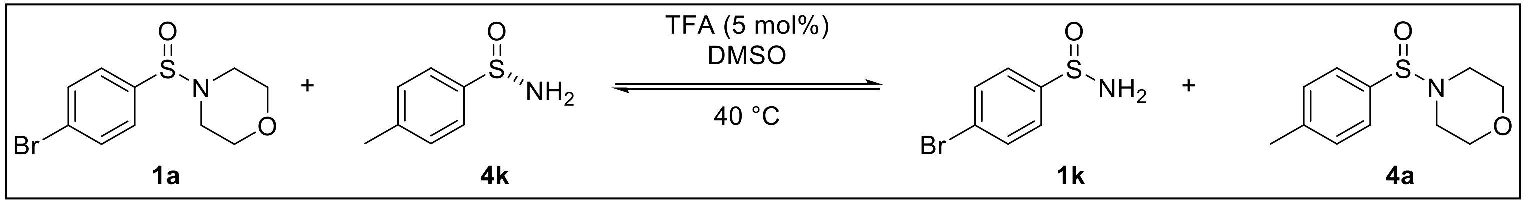

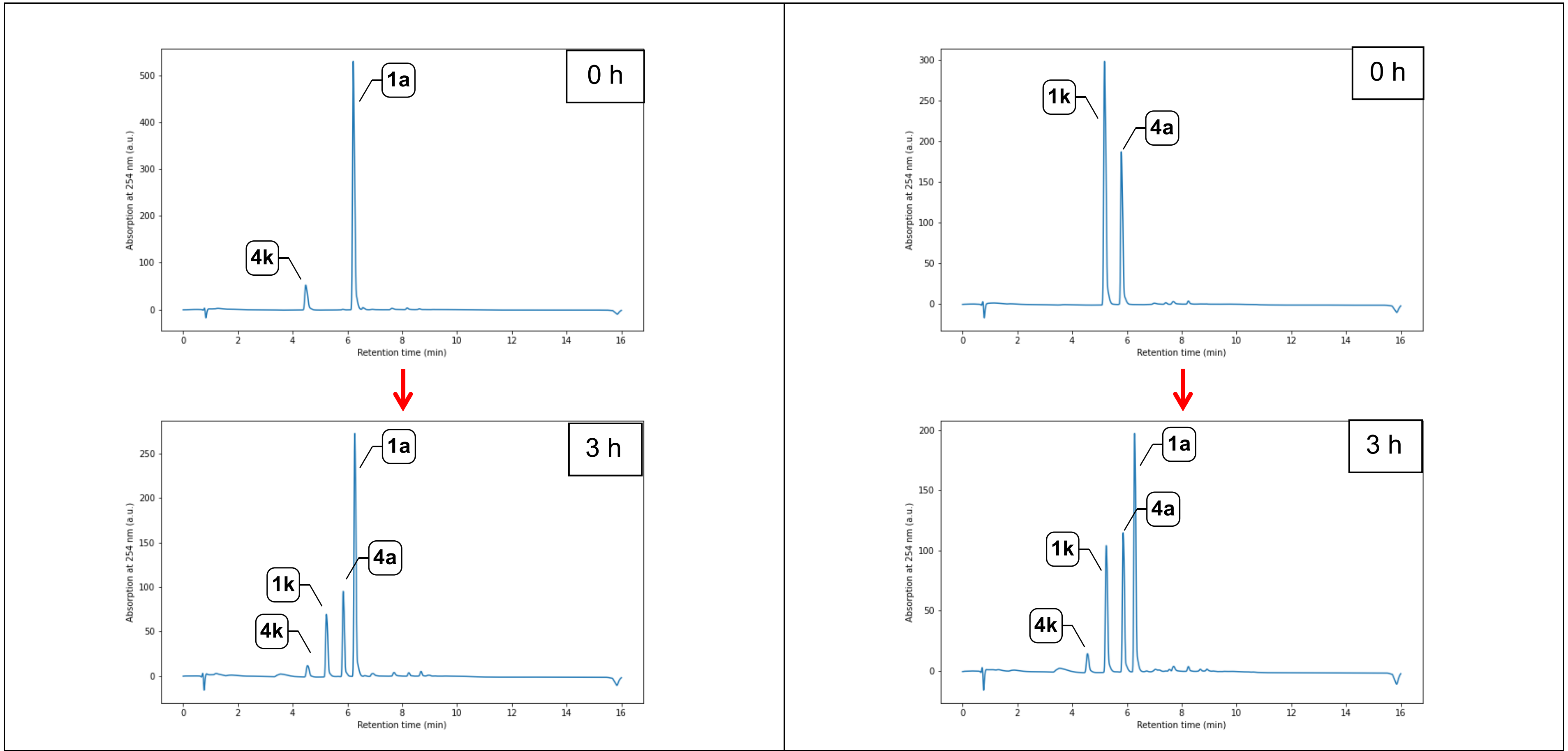

Total yield of sulfinamide products = **70 %**.
Molar ratio of products:
1a : **4k** : **1k** : **4a** = 1.6 : 0.6 : 0.6. : 1.4

Total yield of sulfinamide products = **77 %**.
Molar ratio of products:
1a : **4k** : **1k** : **4a** = 1.1 : 0.6 : 0.9 : 1.6

Figure S10. HPLC chromatograms of the crossover reaction of **1a**, **4k** and **1k**, **4a**.

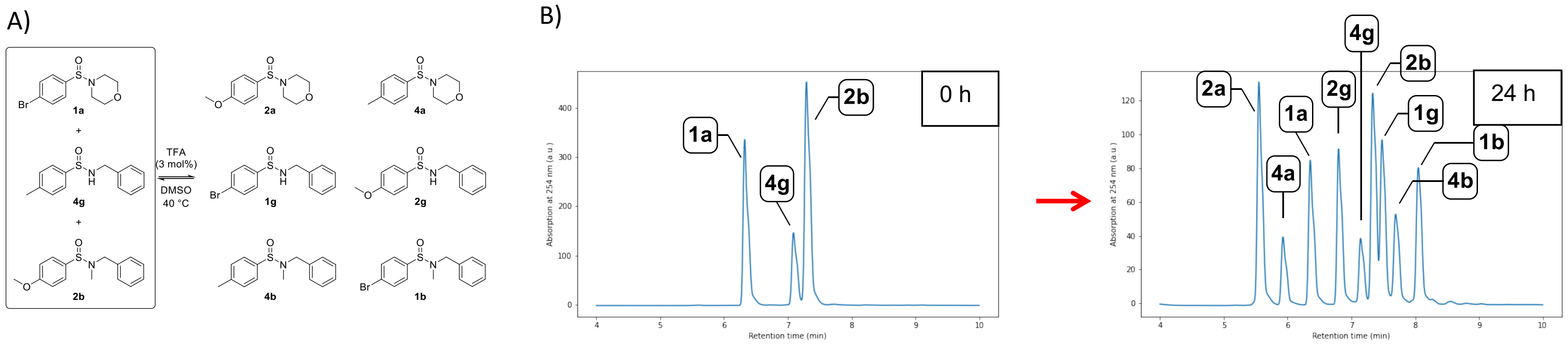

Figure S11. A) A scheme of the crossover reaction starting from substrates **1a**, **4g** and **2b**. B) HPLC chromatograms of the crossover reaction of substrates **1a**, **4g** and **2b** at the reaction times of 0 h and 24 h.

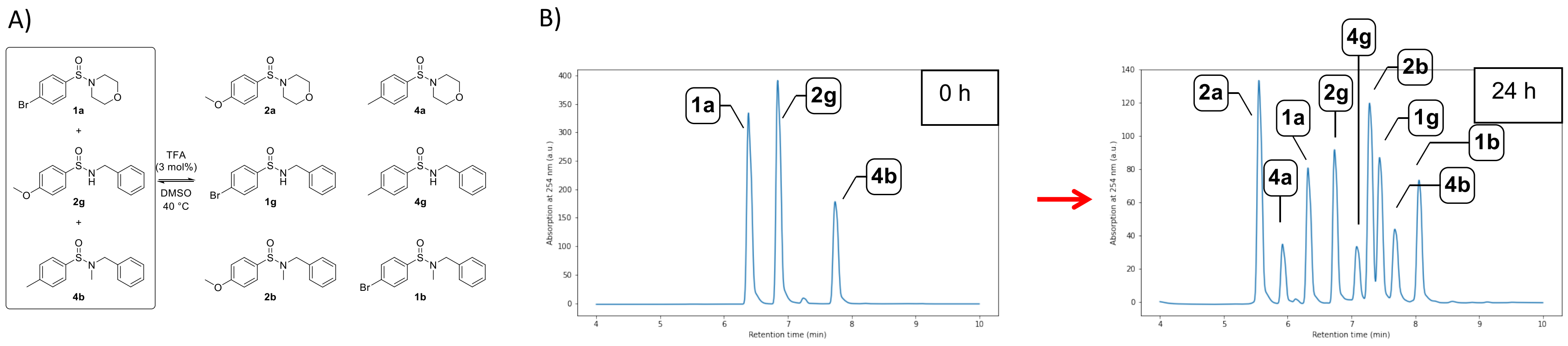

Figure S12. A) A scheme of the crossover reaction starting from substrates **1a**, **2g** and **4b**. B) HPLC chromatograms of the crossover reaction of substrates **1a**, **2g** and **4b** at the reaction times of 0 h and 24 h.

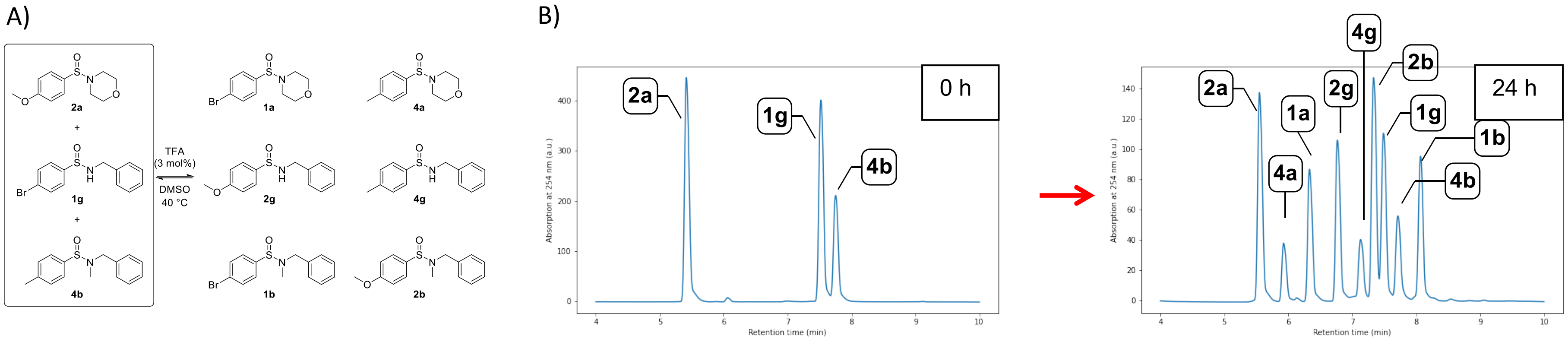

Figure S13. A) A scheme of the crossover reaction starting from substrates **2a**, **1g** and **4b**. B) HPLC chromatograms of the crossover reaction of substrates **2a**, **1g** and **4b** at the reaction times of 0 h and 24 h.

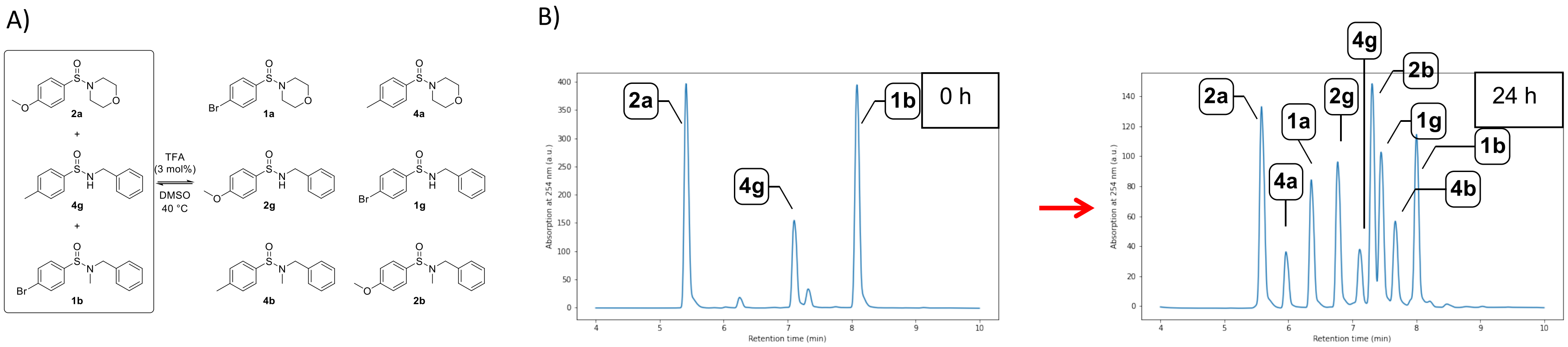

Figure S14. A) A scheme of the crossover reaction starting from substrates **2a**, **4g** and **1b**. B) HPLC chromatograms of the crossover reaction of substrates **2a**, **4g** and **1b** at the reaction times of 0 h and 24 h.

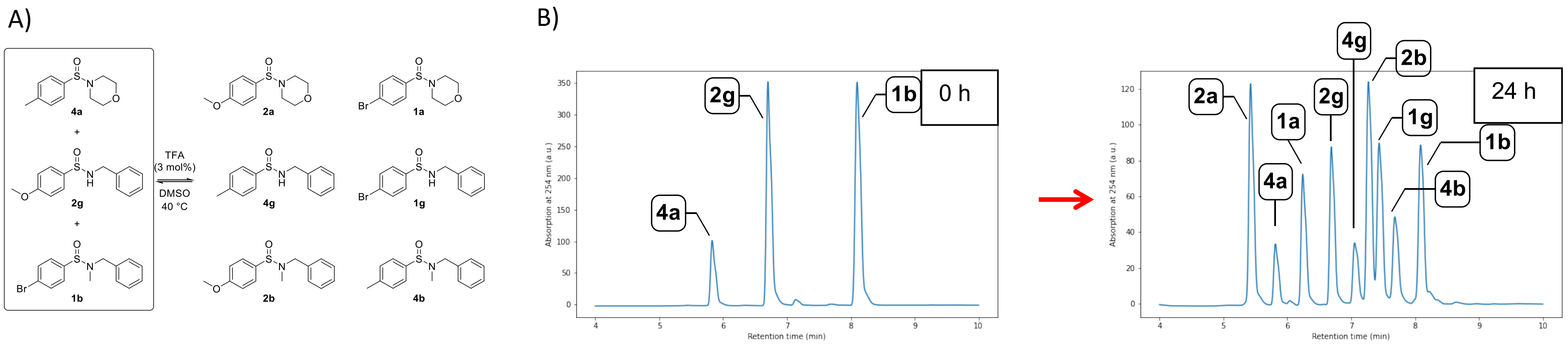

Figure S15. A) A scheme of the crossover reaction starting from substrates **4a**, **2g** and **1b**. B) HPLC chromatograms of the crossover reaction of substrates **4a**, **2g** and **1b** at the reaction times of 0 h and 24 h.

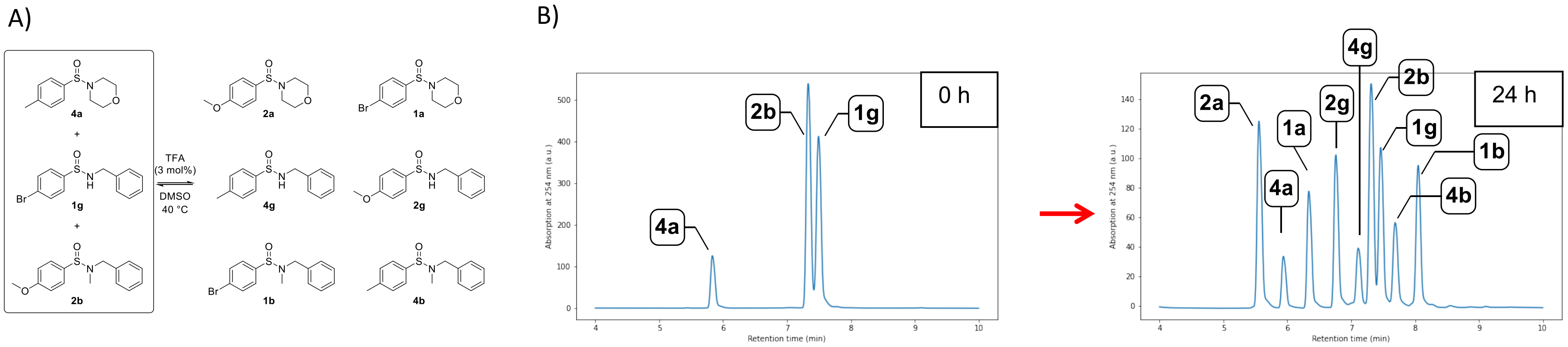

Figure S16. A) A scheme of the crossover reaction starting from substrates **4a**, **1g** and **2b**. B) HPLC chromatograms of the crossover reaction of substrates **4a**, **1g** and **2b** at the reaction times of 0 h and 24 h.

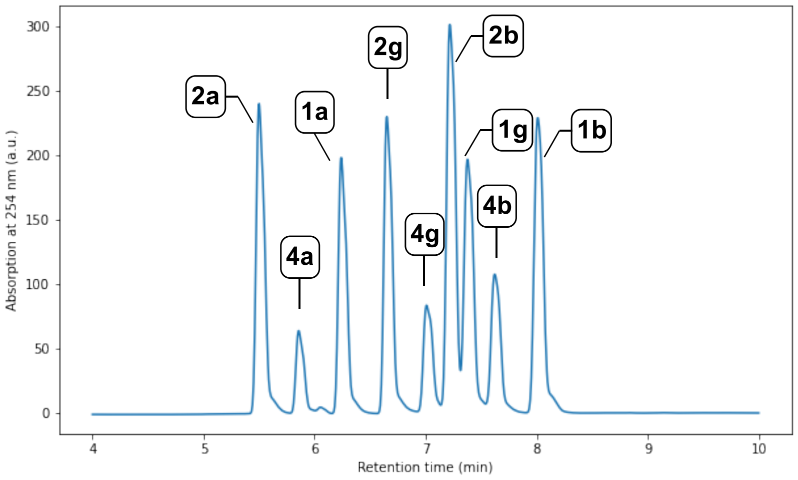

Figure S17. HPLC chromatogram of an equimolar mixture of nine sulfinamides **2a**, **4a**, **1a**, **2g**, **4g**, **2b**, **1g**, **4b** and **1b**.

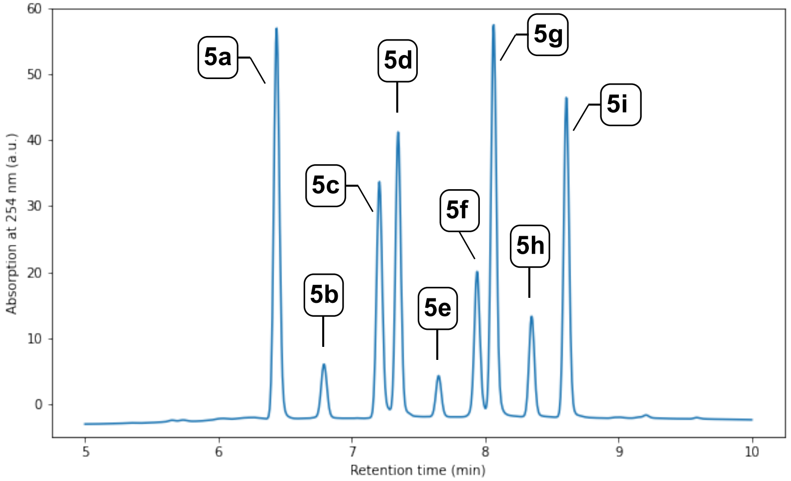

Figure S18. HPLC chromatogram of an equimolar mixture of sulfonamides **5a-i**.

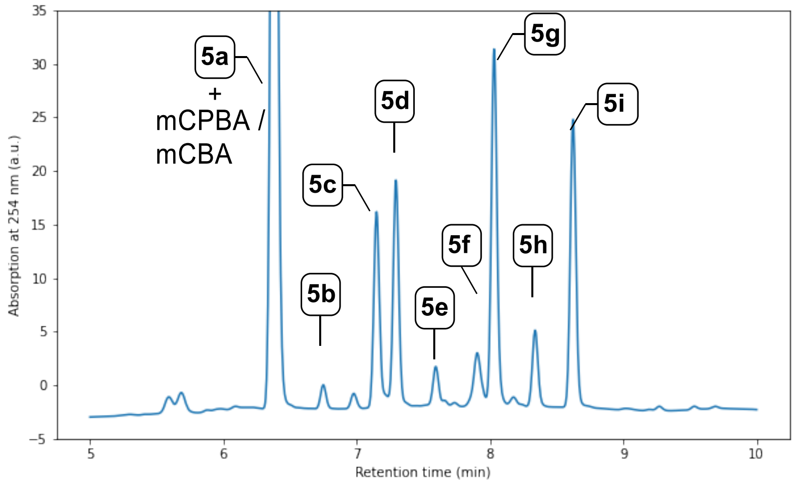

Figure S19. HPLC chromatogram of the oxidation reaction of an equimolar mixture of sulfinamides **2a**, **4a**, **1a**, **2g**, **4g**, **2b**, **1g**, **4b** and **1b** leading to sulfonamides **5a-i**.

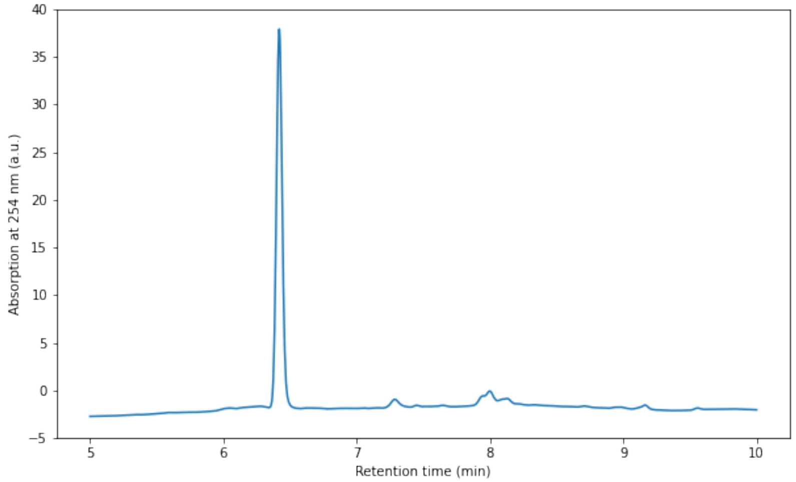

Figure S20. HPLC chromatogram of a solution of mCPBA (9 mg) in CH_2Cl_2 (0.5 ml) incubated at rt for 5 min.

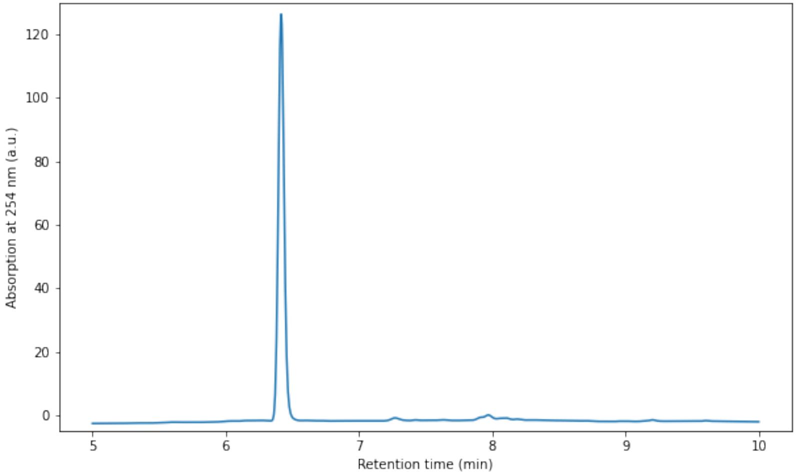

Figure S21. HPLC chromatogram of a solution of mCPBA (9 mg) in DMSO (0.5 ml) incubated at rt for 5 min.

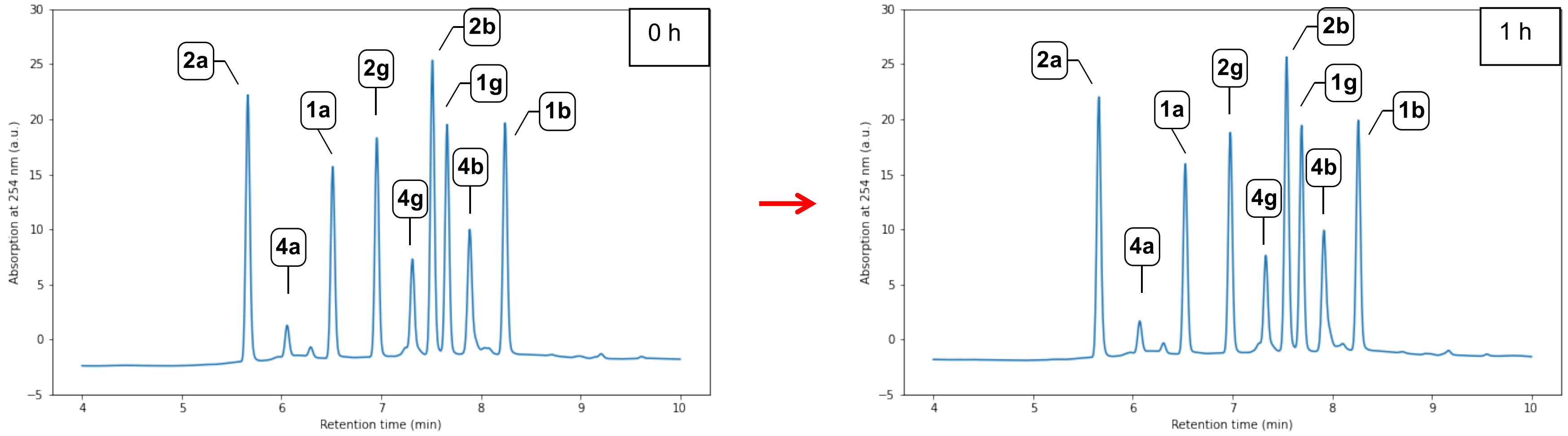

Figure S22. HPLC chromatograms of an equimolar mixture of sulfinamides **2a**, **4a**, **1a**, **2g**, **4g**, **2b**, **1g**, **4b** and **1b** incubated in a neutral aqueous solution for 1 h.

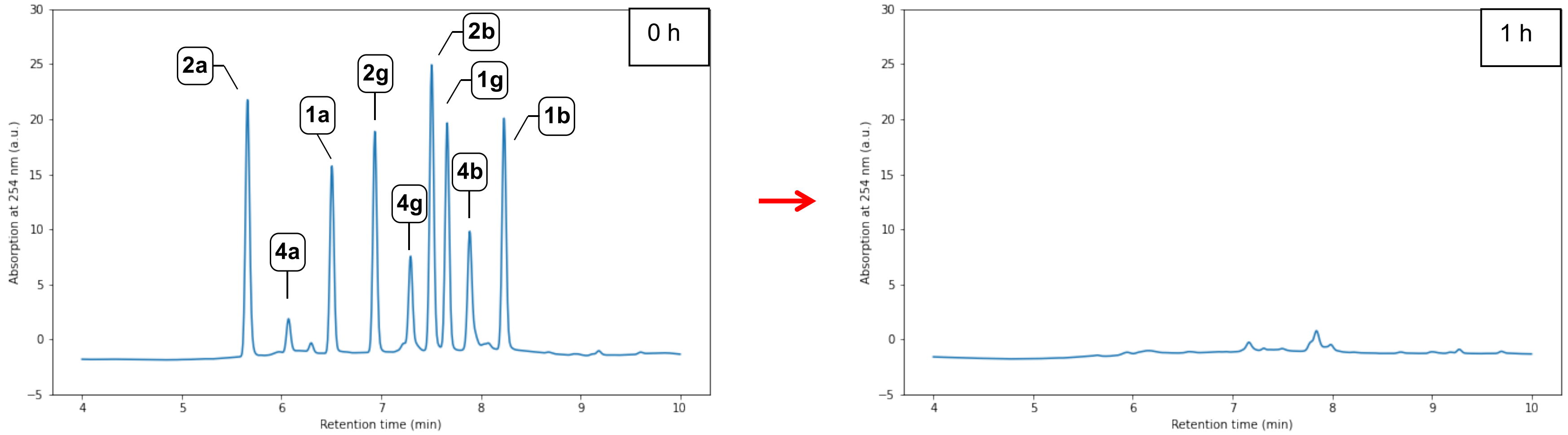

Figure S23. HPLC chromatograms of an equimolar mixture of sulfinamides **2a**, **4a**, **1a**, **2g**, **4g**, **2b**, **1g**, **4b** and **1b** incubated in an acidic aqueous solution for 1 h.

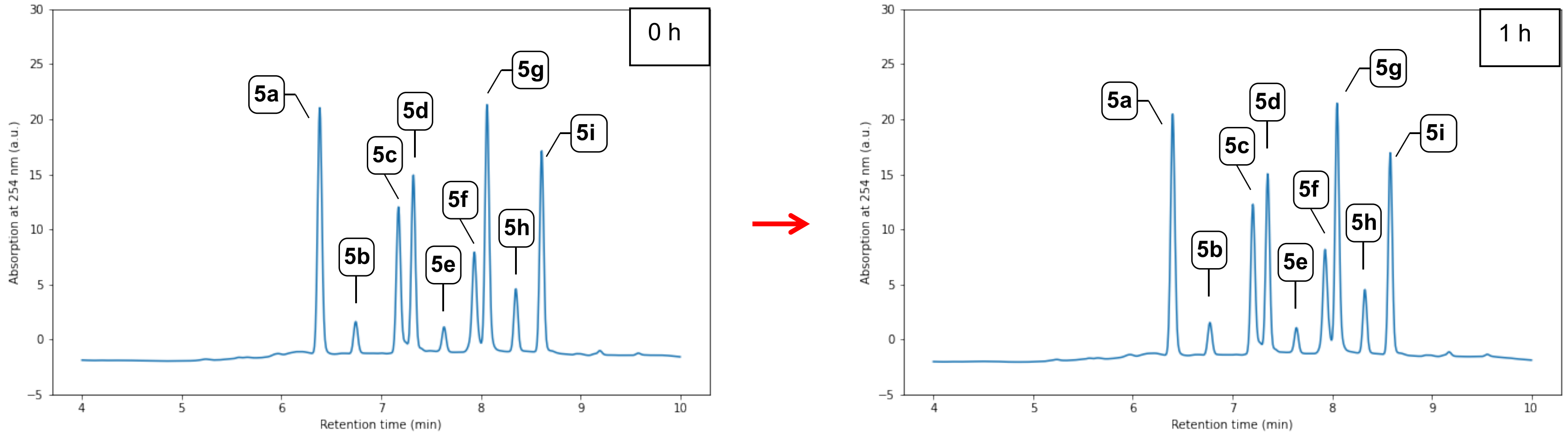

Figure S24. HPLC chromatograms of an equimolar mixture of sulfonamides **5a-l** incubated in a neutral aqueous solution for 1 h.

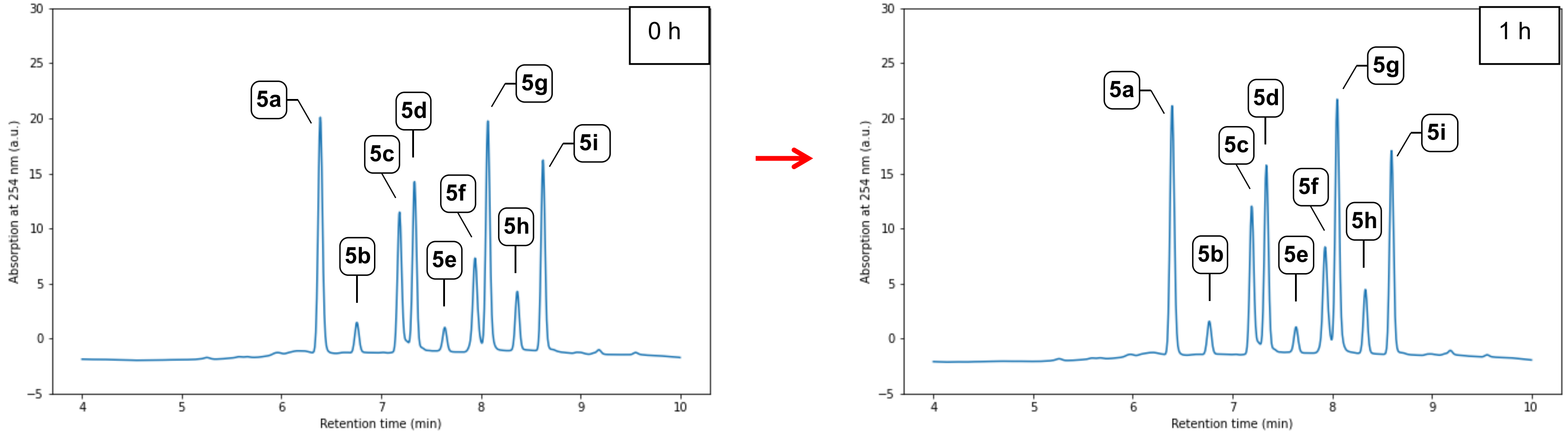

Figure S25. HPLC chromatograms of an equimolar mixture of sulfonamides **5a-l** incubated in an acidic aqueous solution for 1 h.

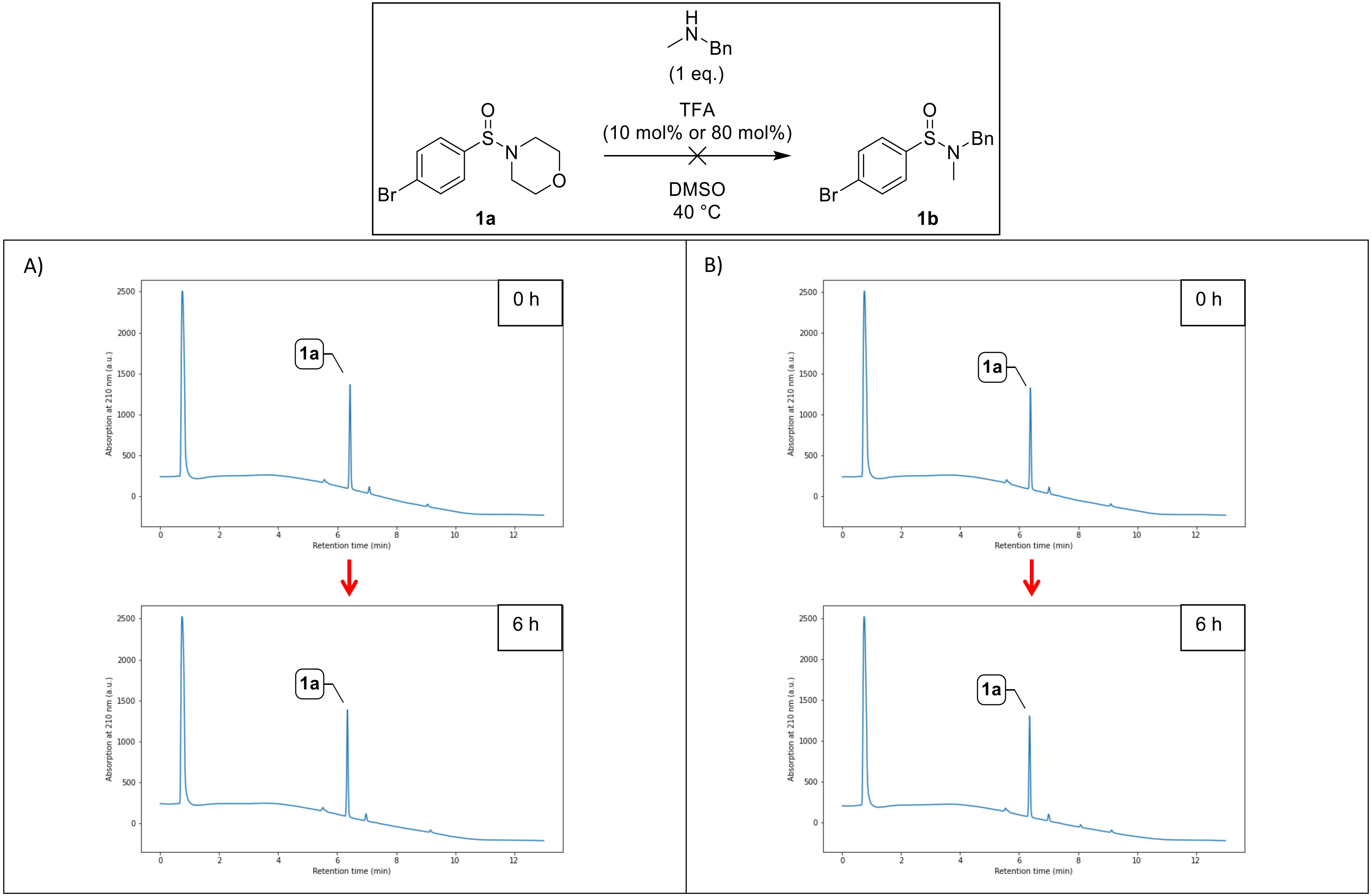

Figure S26. HPLC chromatograms of the reaction of **1a** with *N*-methyl-1-phenylmethanamine and A) TFA 10 mol %, B) TFA 80 mol %.

12. Time course of the Reaction of 1a and 2b

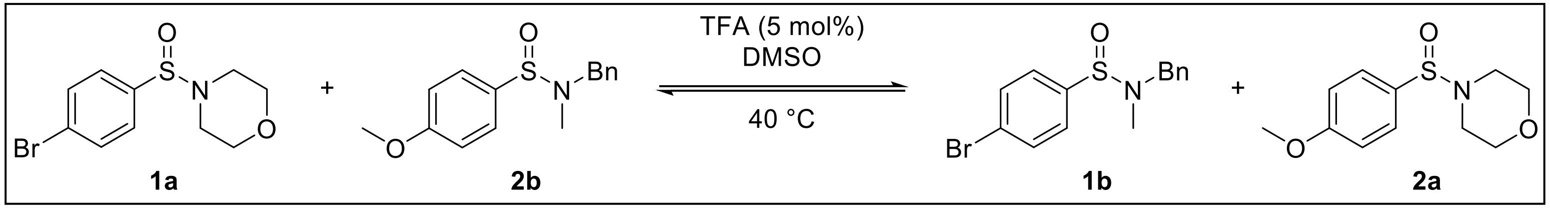

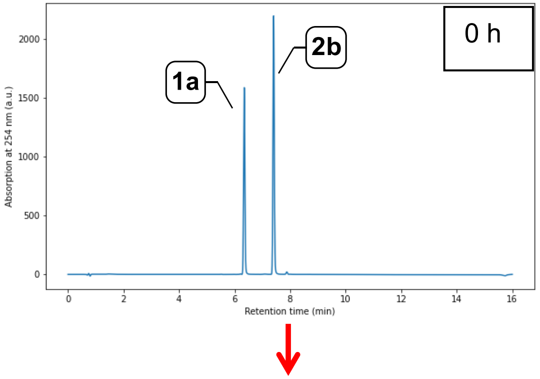

Molar ratio of products:

1a : **2b** : **1b** : **2a** = 1.0 : 1.0 : 0.0 : 0.0

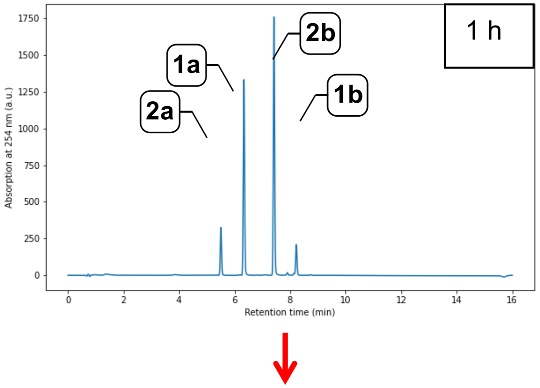

Total yield of sulfinamide products = **94%**

Molar ratio of products:

1a : **2b** : **1b** : **2a** = 1.7 : 1.7 : 0.2 : 0.3

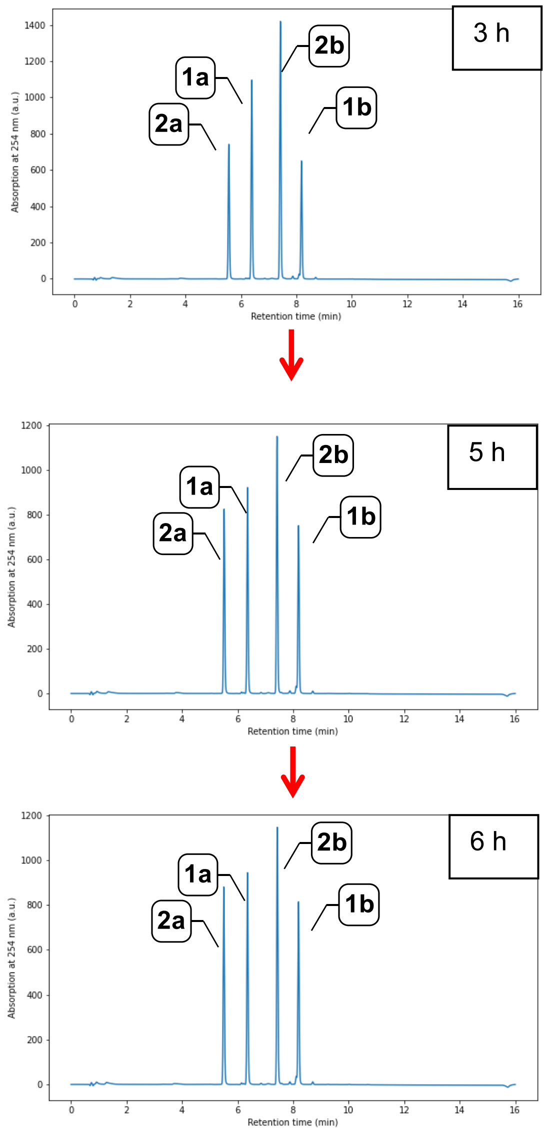

Total yield of sulfinamide products = **92%**
Molar ratio of products:
1a : **2b** : **1b** : **2a** = 1.3 : 1.3 : 0.7 : 0.7

Total yield of sulfinamide products = **91%**
Molar ratio of products:
1a : **2b** : **1b** : **2a** = 1.2 : 1.1 : 0.8 : 0.9

Total yield of sulfinamide products = **88%**
Molar ratio of products:
1a : **2b** : **1b** : **2a** = 1.2 : 1.1 : 0.9 : 0.9

Figure S27. HPLC chromatograms of the crossover reaction of **1a** and **2b**.

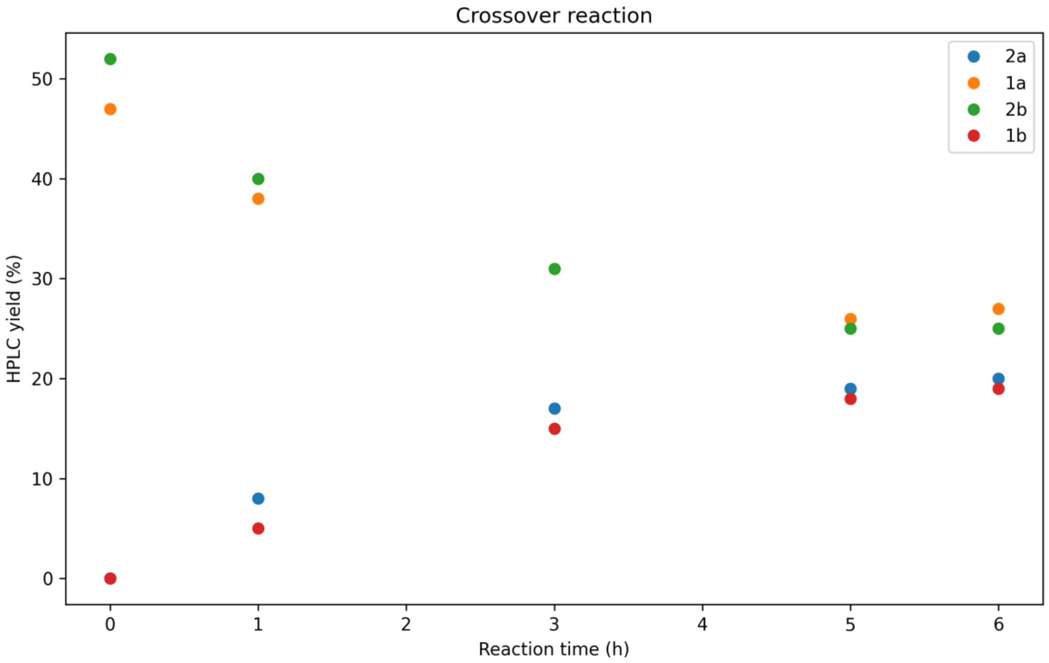

Figure S28: Timecourse of the crossover reaction of **1a** and **2b**.

13. Extracted HPLC-MS Chromatograms of Solvent and Acid Screens

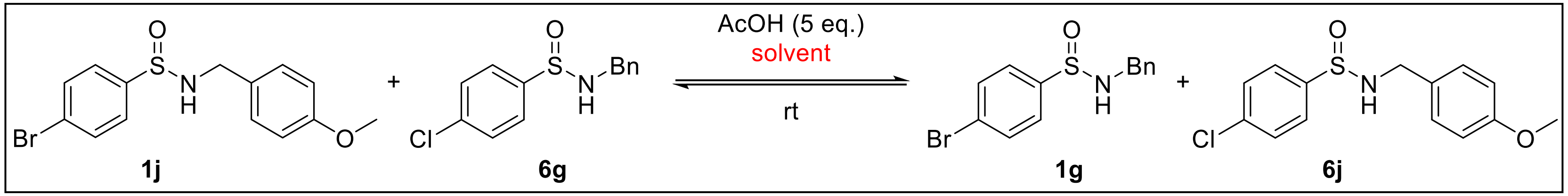

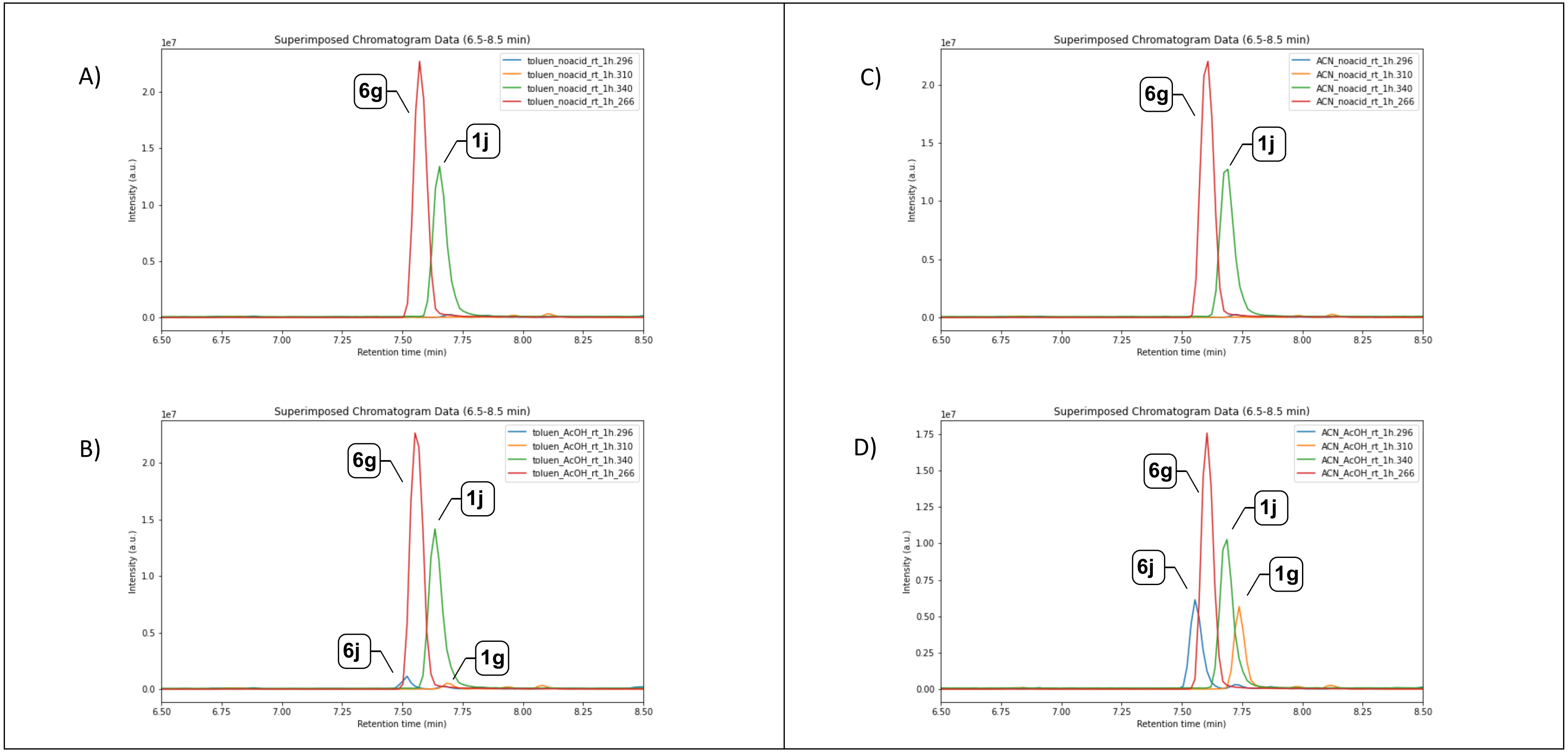

Figure S29. Extracted HPLC-MS chromatograms of the preliminary screening of the crossover reaction of **1j** and **6g** in different solvents. Superimposed extracted chromatograms of products **1j** (*m/z* [M+H$^+$] = 340), **6g** (*m/z* [M+H$^+$] = 266) and **1g** (*m/z* [M+H$^+$] = 310), **6j** (*m/z* [M+H$^+$] = 296) at 1h at rt; A) in toluene without acid (blank), B) in toluene with AcOH, C) in CH_3CN without acid (blank), D) in CH_3CN with AcOH.

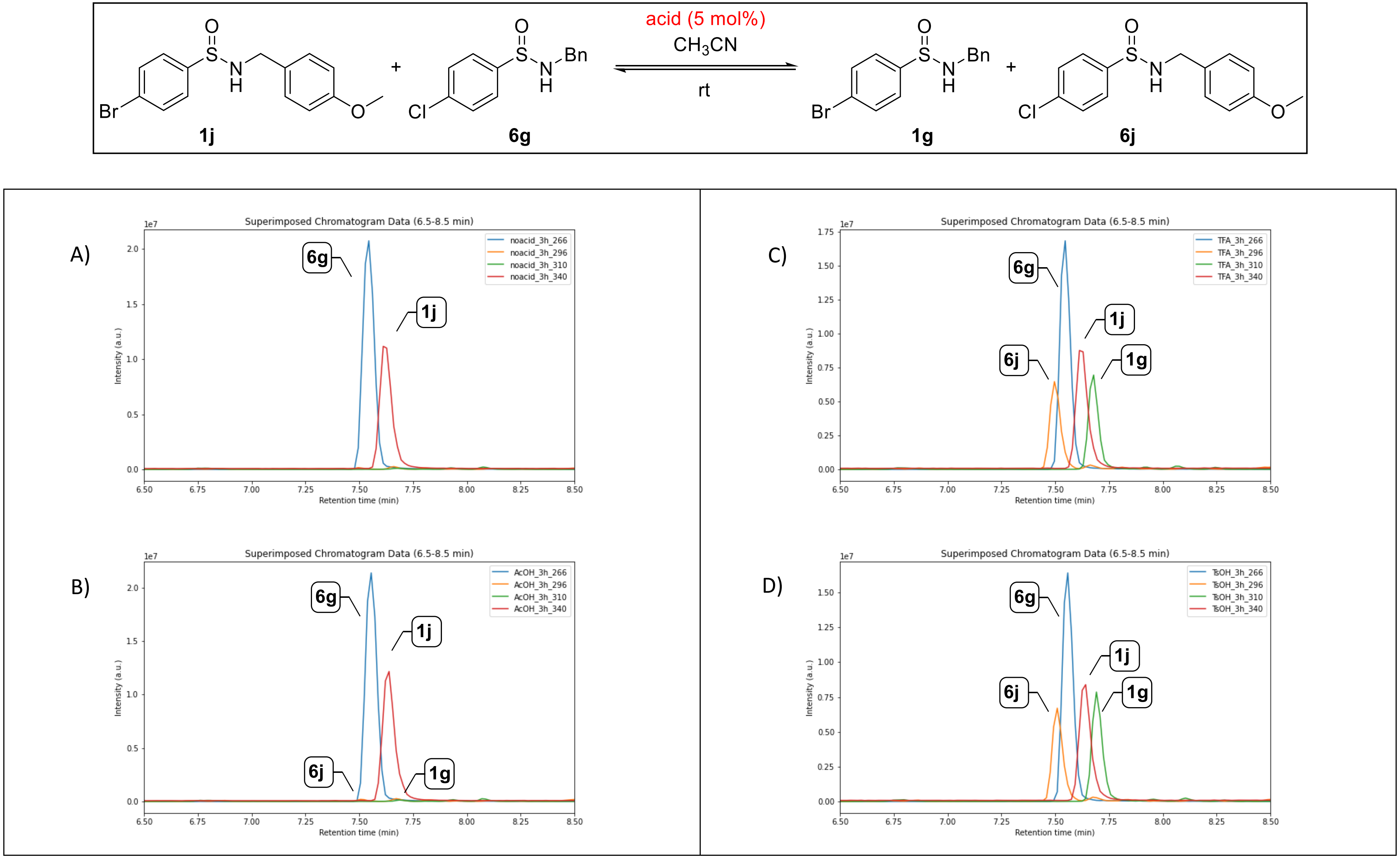

Figure S30. Extracted HPLC-MS chromatograms of the preliminary screening of the crossover reaction of **1j** and **6g** in CH_3CN with different acids. Superimposed extracted chromatograms of products **1j** (*m/z* $[M+H^+]$ = 340), **6g** (*m/z* $[M+H^+]$ = 266) and **1g** (*m/z* $[M+H^+]$ = 310), **6j** (*m/z* $[M+H^+]$ = 296) at 3h at rt; A) without acid (blank), B) AcOH, C) TFA, D) $TsOH{\cdot}H_2O$.

14. Extracted HPLC-MS Chromatograms of Sulfinamide 4i Under Acidic Conditions

A)

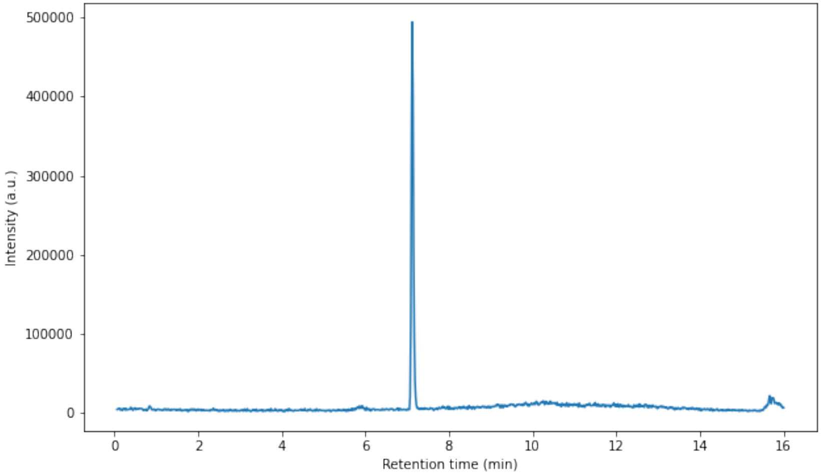

B)

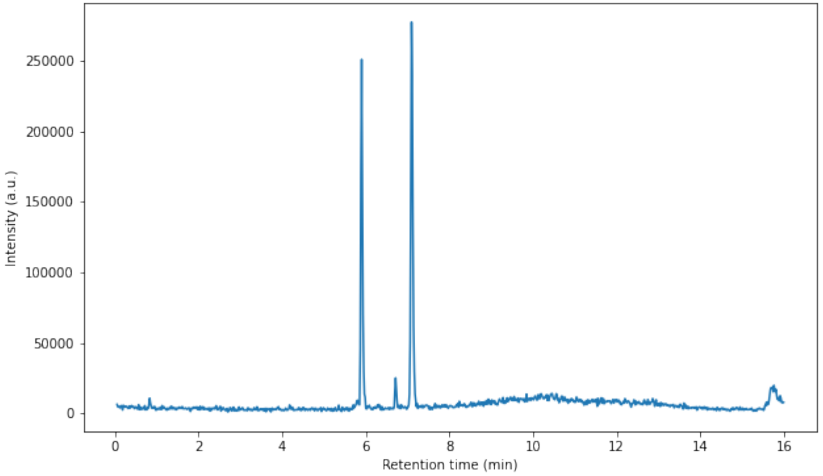

Figure S31. Extracted HPLC-MS chromatograms of sulfinamide **4j** (*m/z* [M+H$^+$] = 232) in CH_3CN at 30°C. A) without acid at 0h, B) with TFA (10 mol %) at 22h.

15. ^{1}H and ^{13}C NMR Spectra

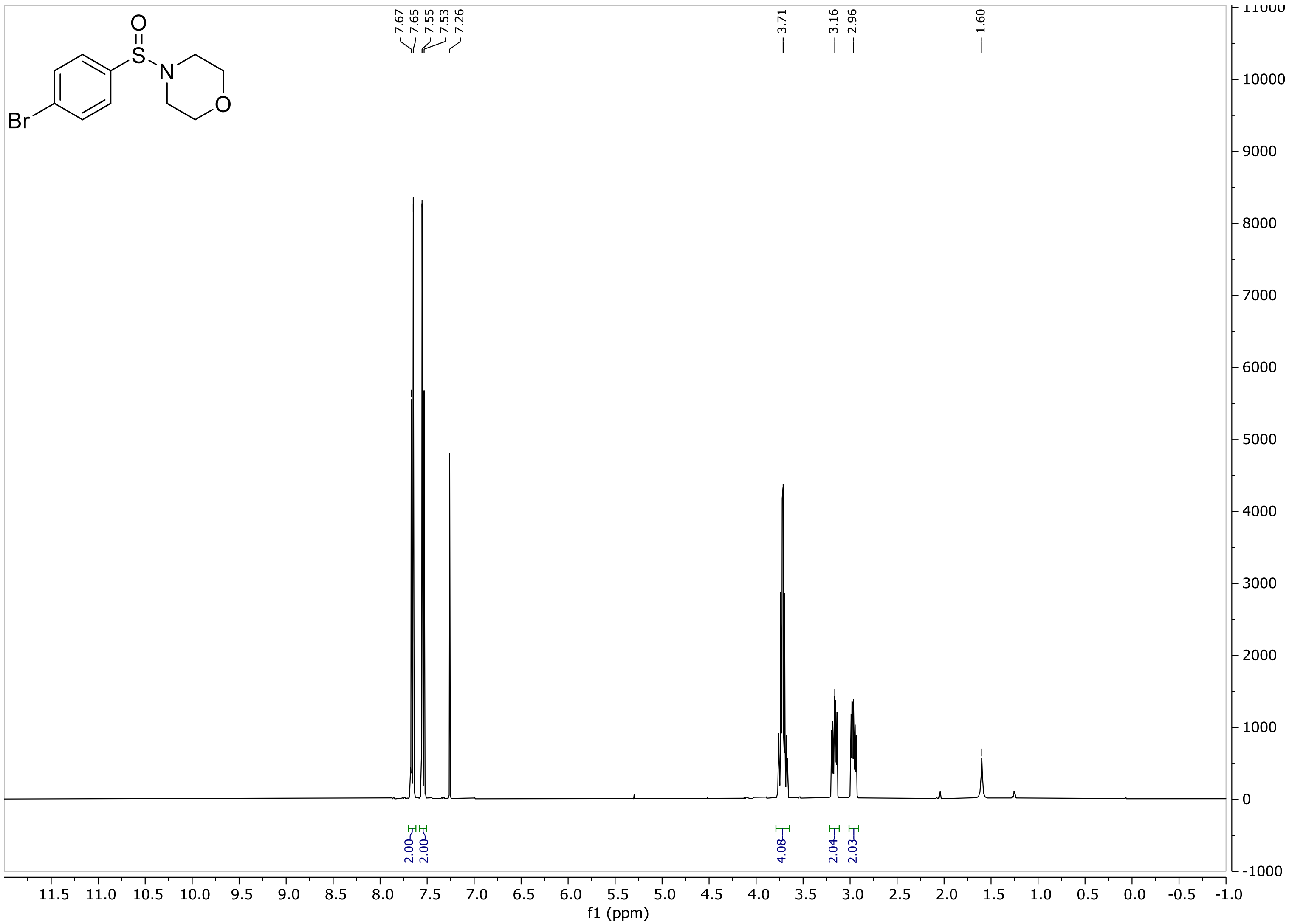

Figure S32. ^{1}H NMR (400 MHz) of **1a** in $CDCl_3$.

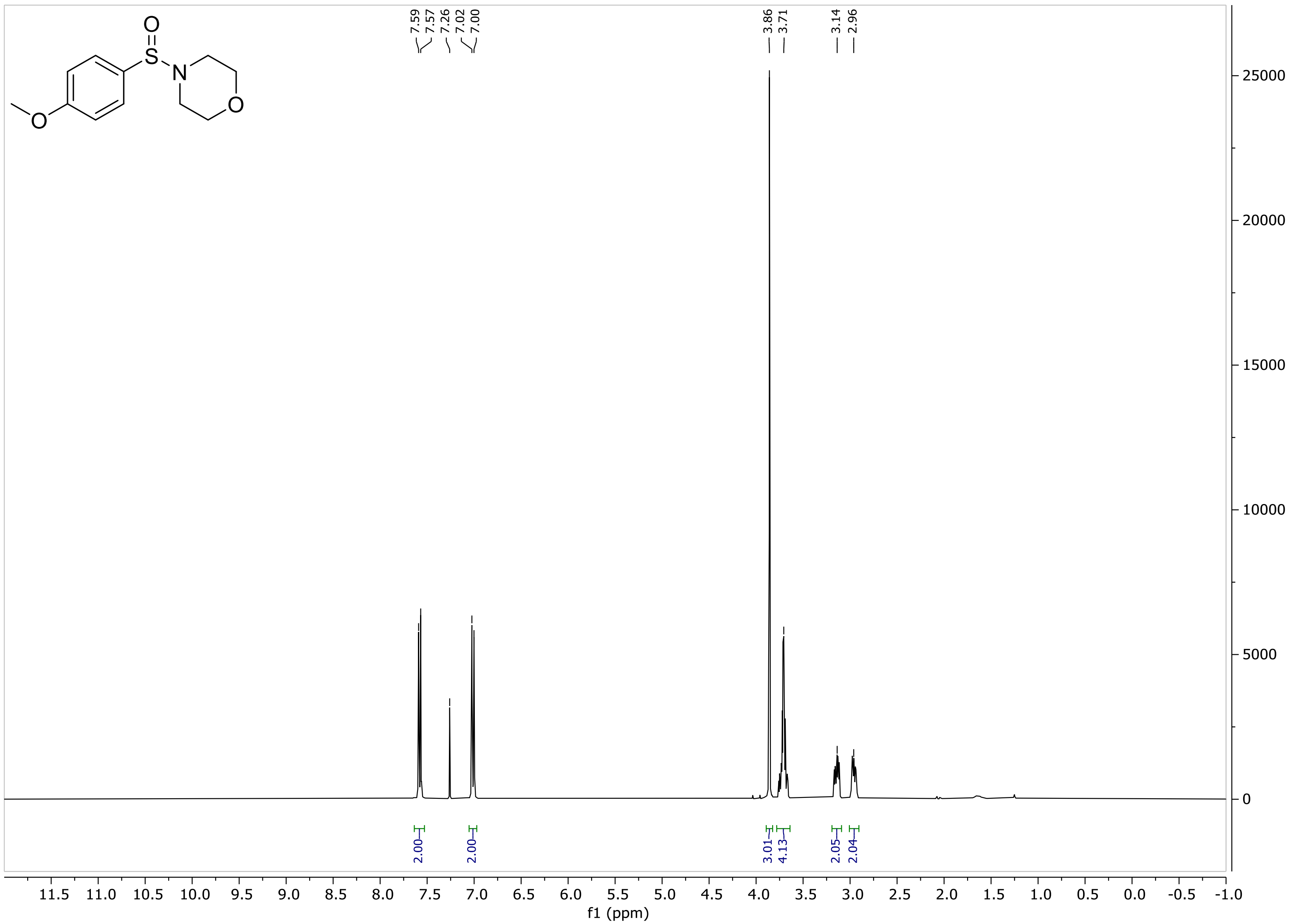

Figure S33. ^{1}H NMR (400 MHz) of **2a** in $CDCl_3$.

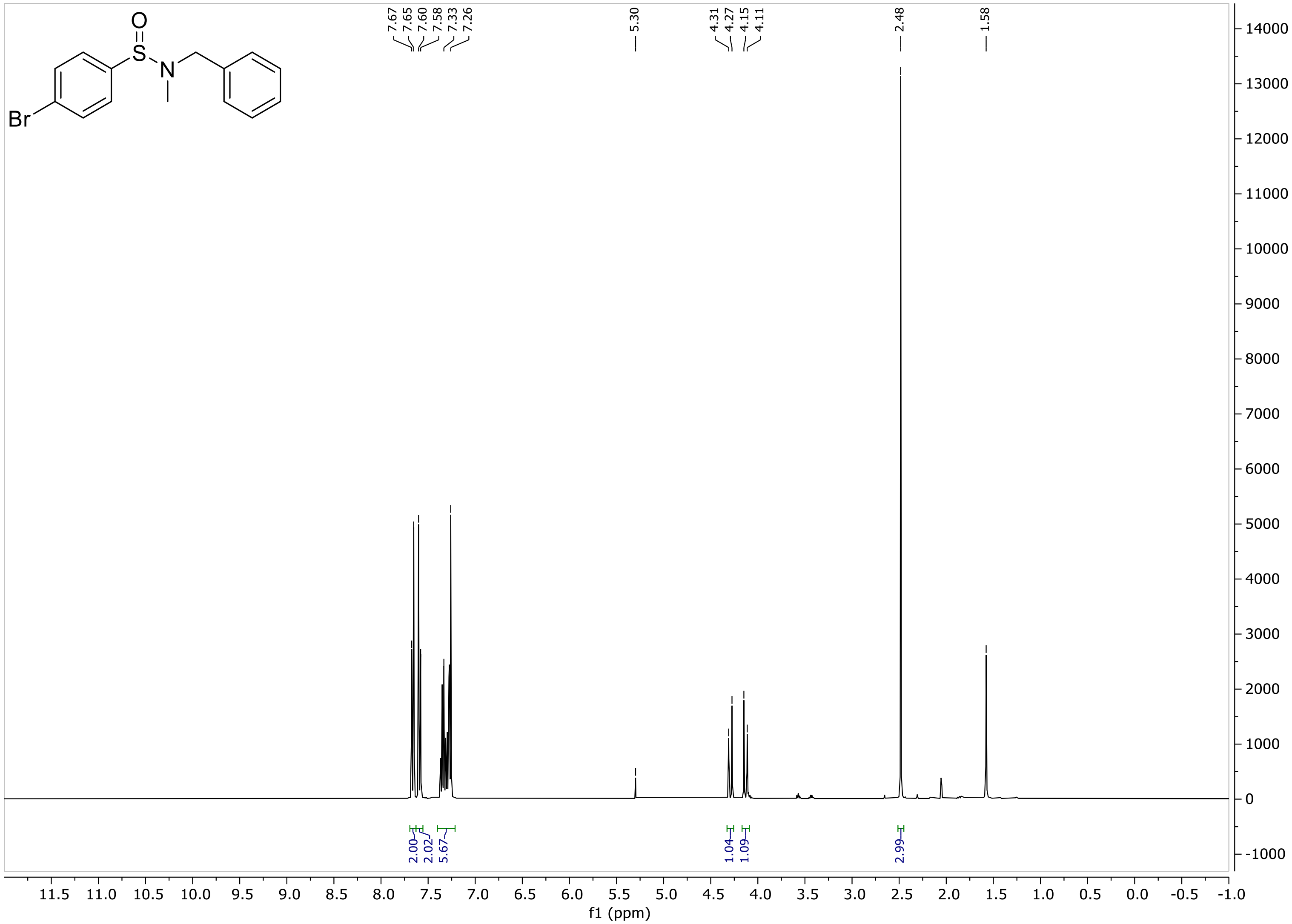

Figure S34. ^{1}H NMR (400 MHz) of **1b** in $CDCl_3$.

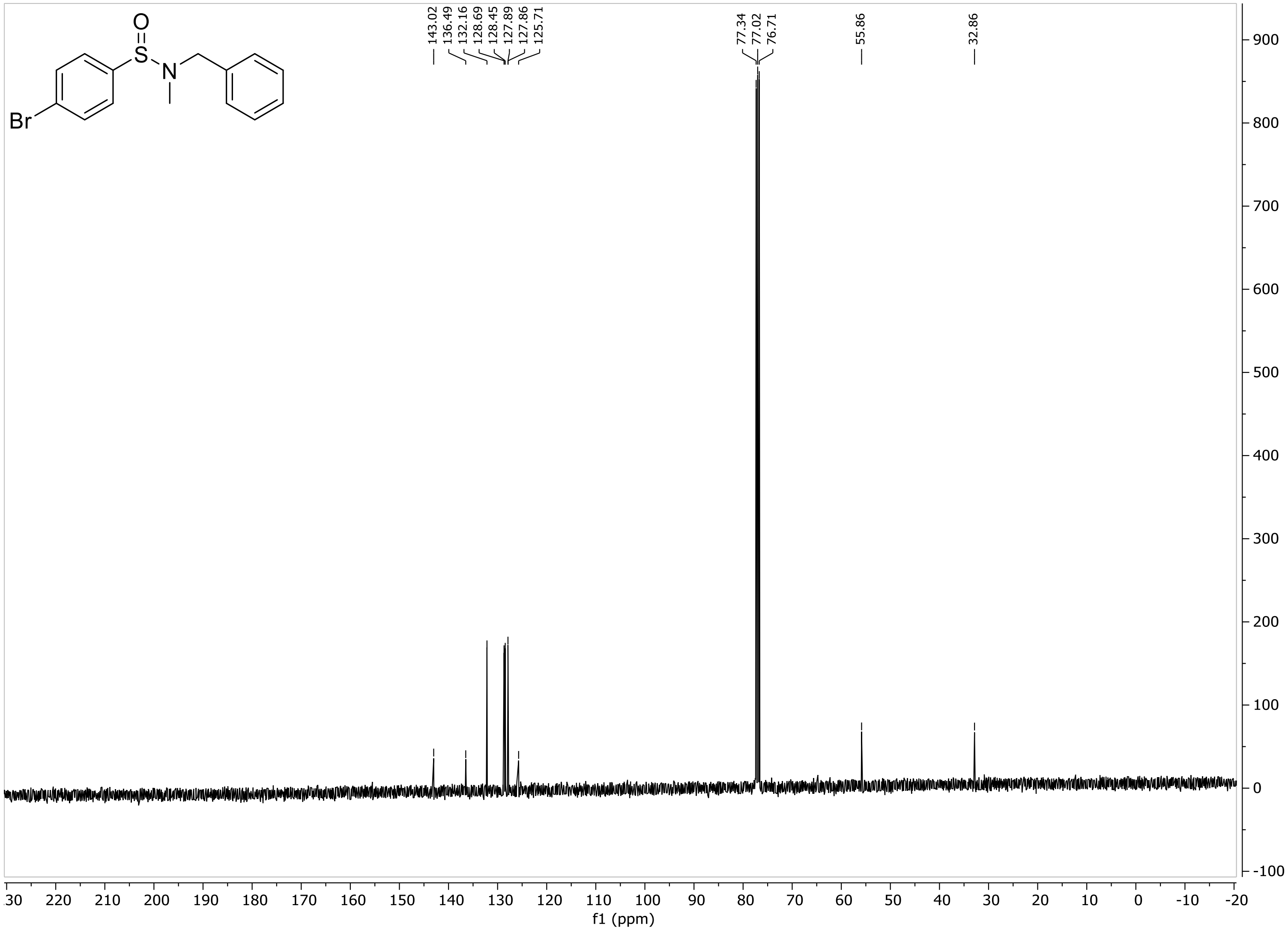

Figure S35. $^{13}C\{^1H\}$ NMR (101 MHz) of **1b** in $CDCl_3$.

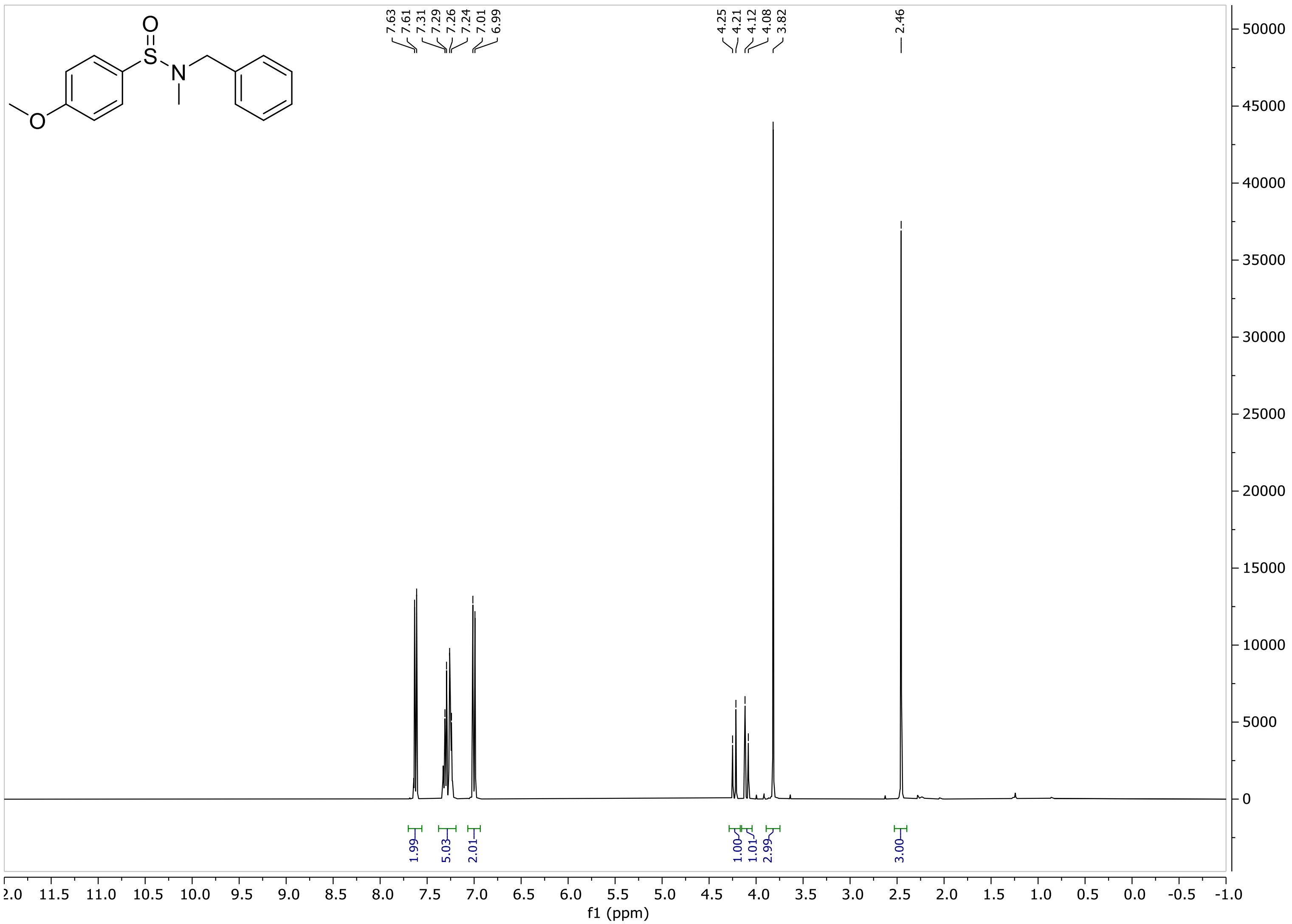

Figure S36. ^{1}H NMR (400 MHz) of **2b** in $CDCl_3$.

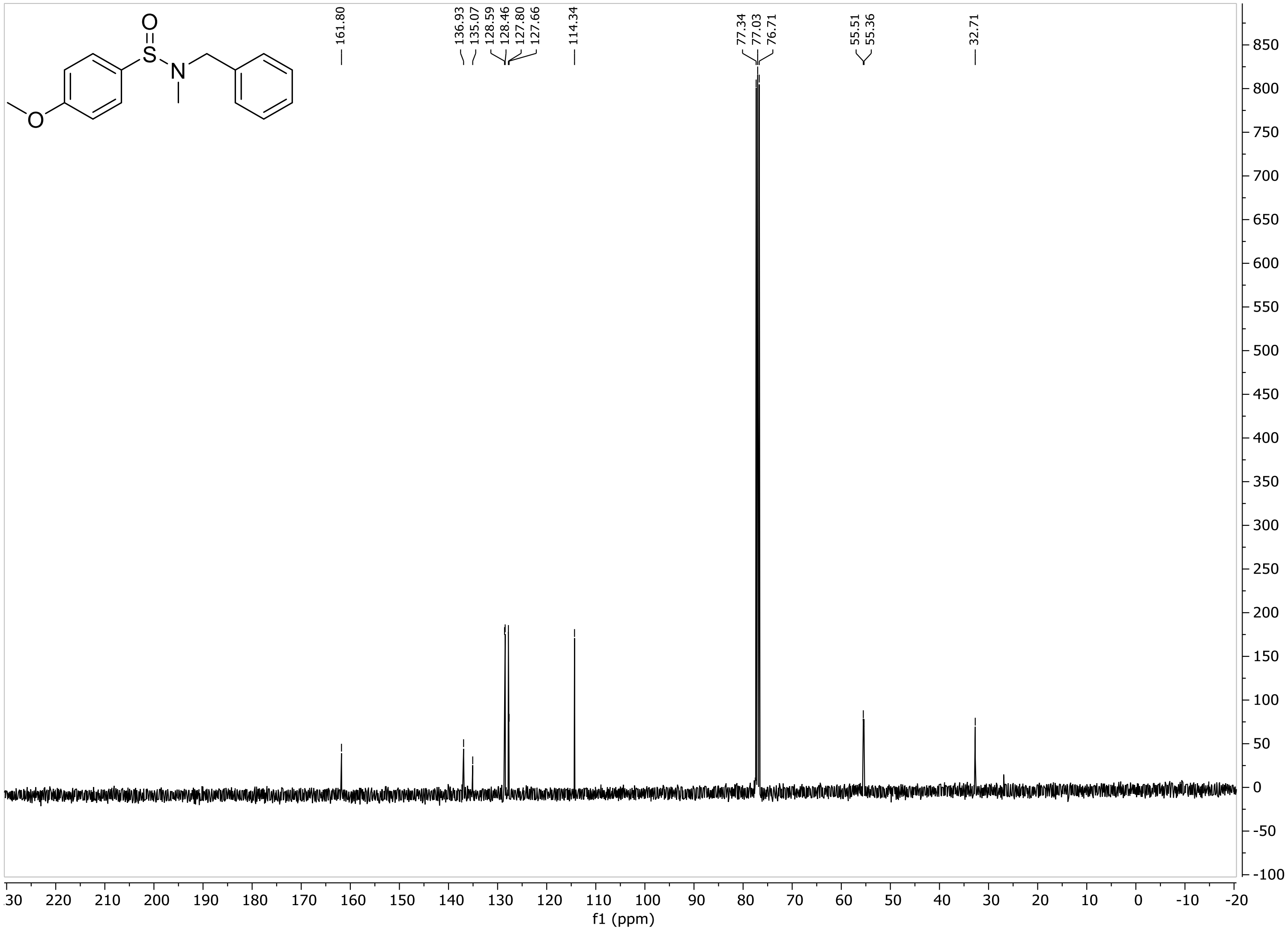

Figure S37. $^{13}C\{^{1}H\}$ NMR (101 MHz) of **2b** in $CDCl_3$.

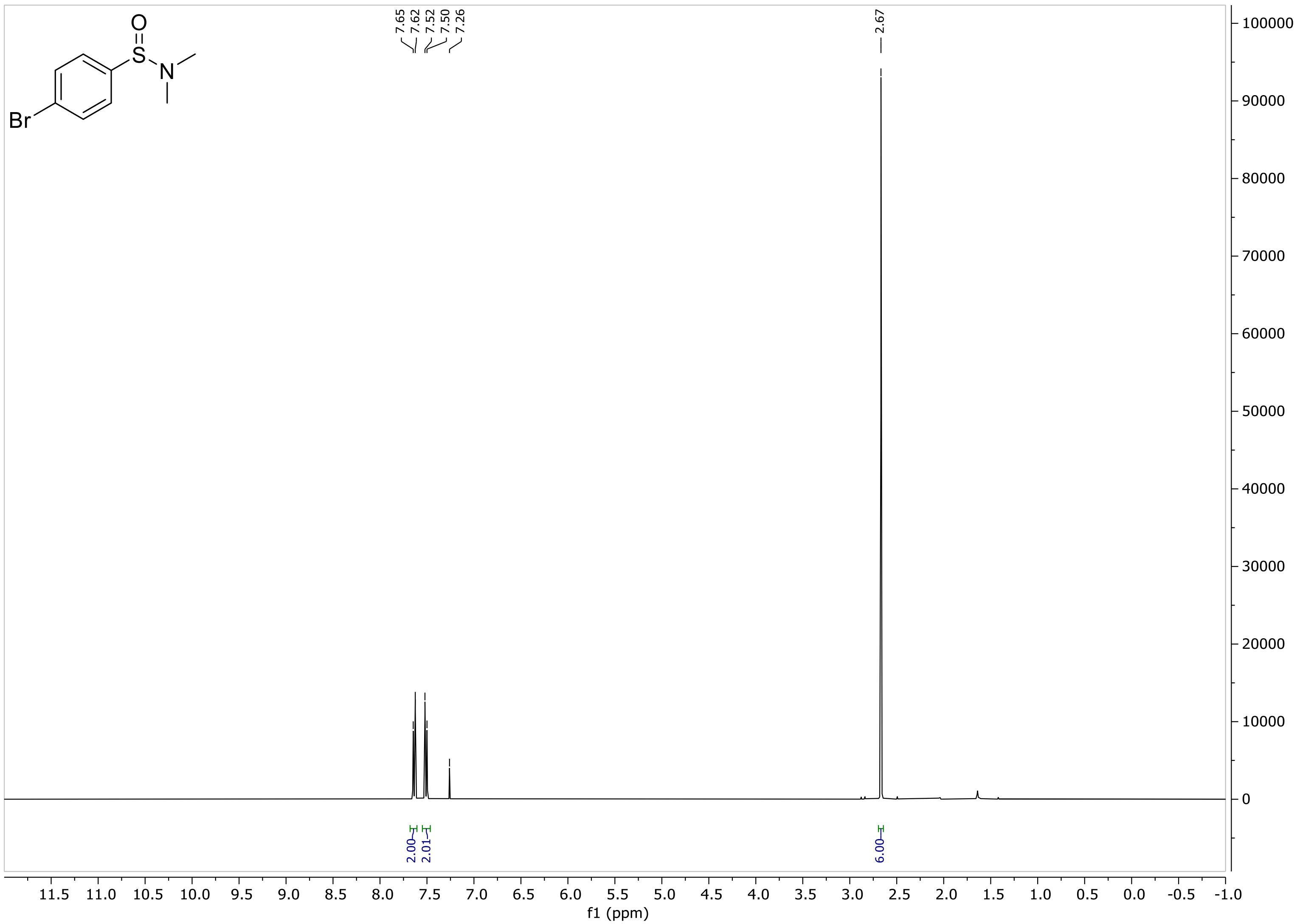

Figure S38. ^{1}H NMR (400 MHz) of **1c** in $CDCl_3$.

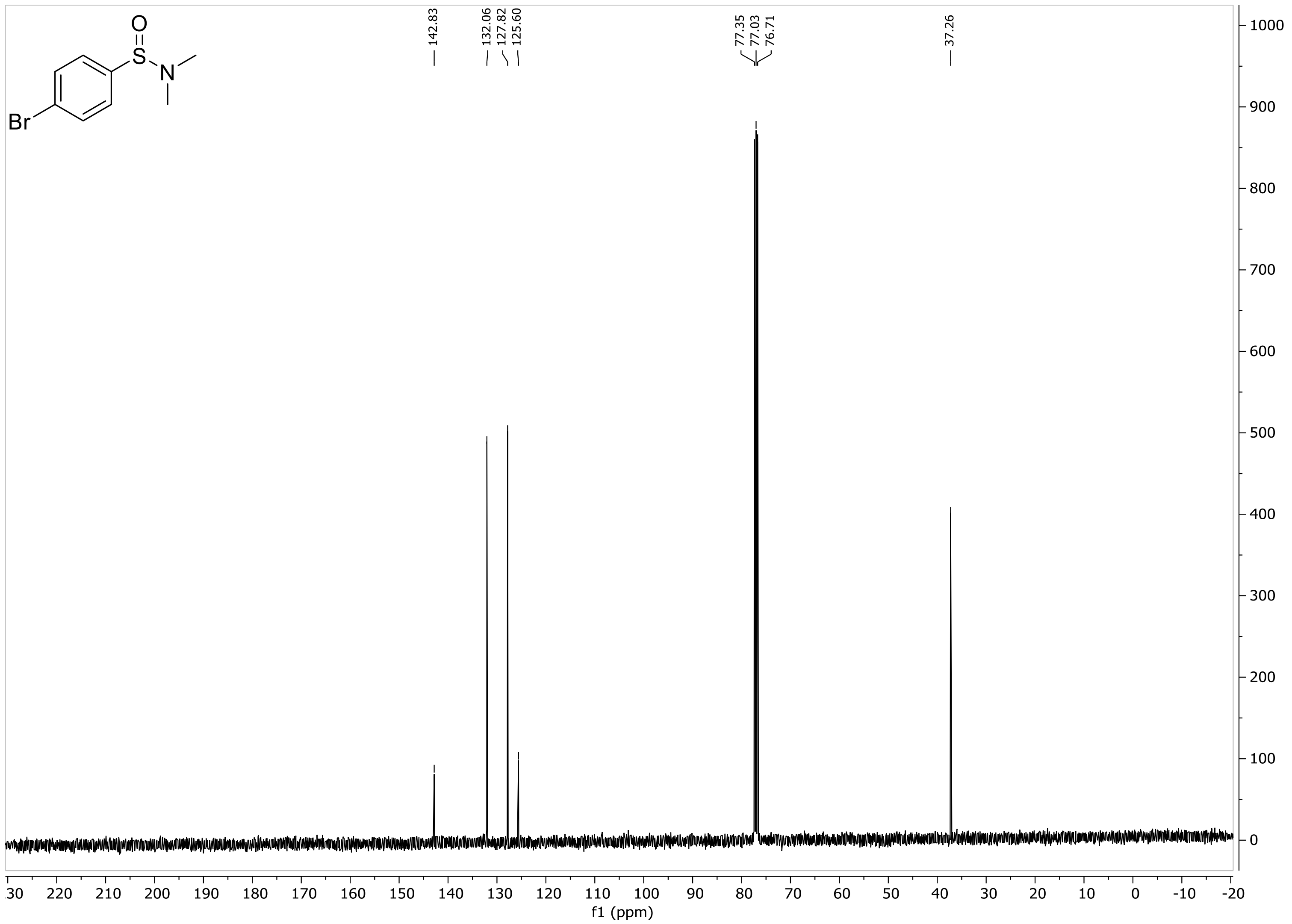

Figure S39. $^{13}C\{^{1}H\}$ NMR (101 MHz) of **1c** in $CDCl_3$.

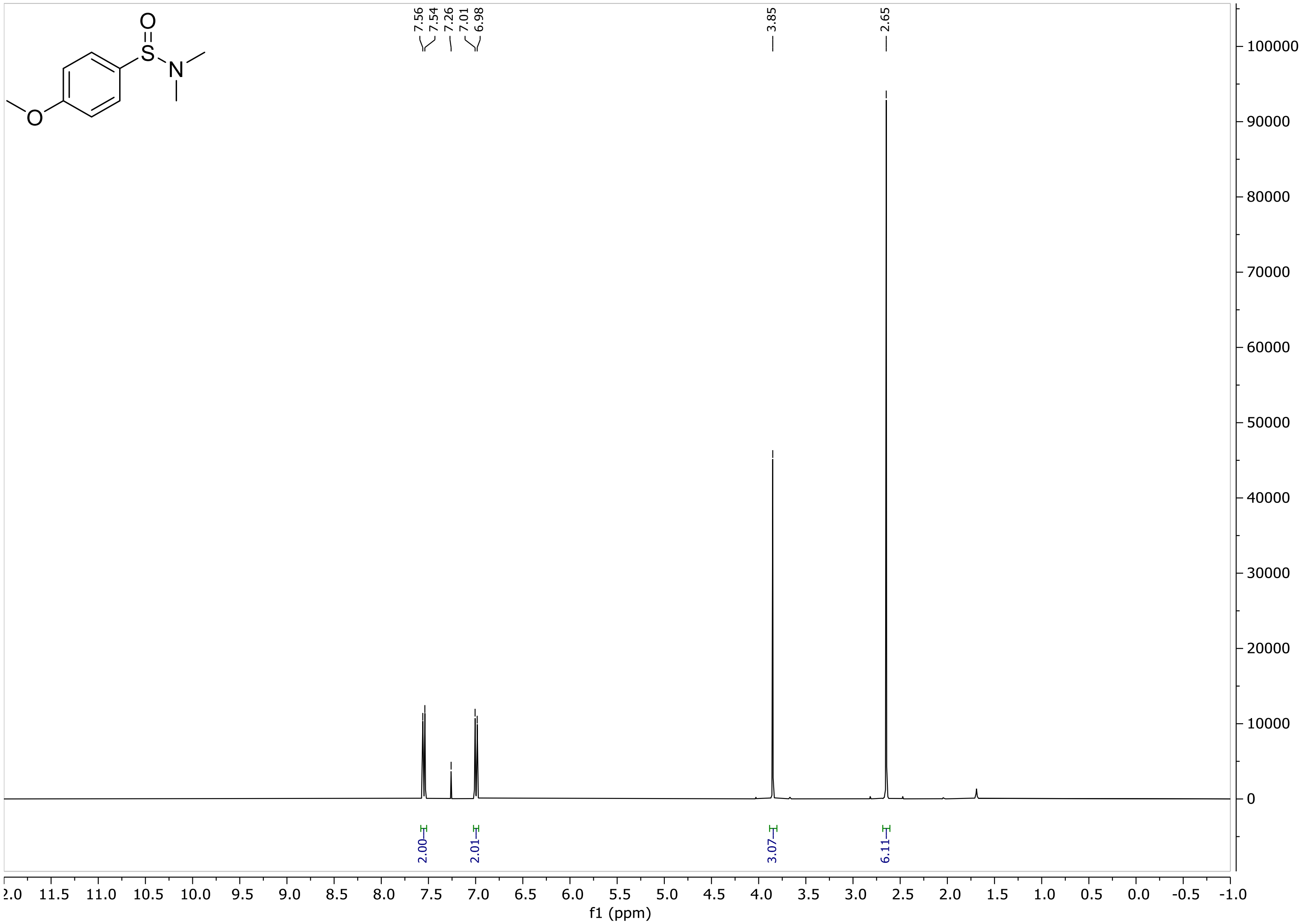

Figure S40. ^{1}H NMR (400 MHz) of **2c** in $CDCl_3$.

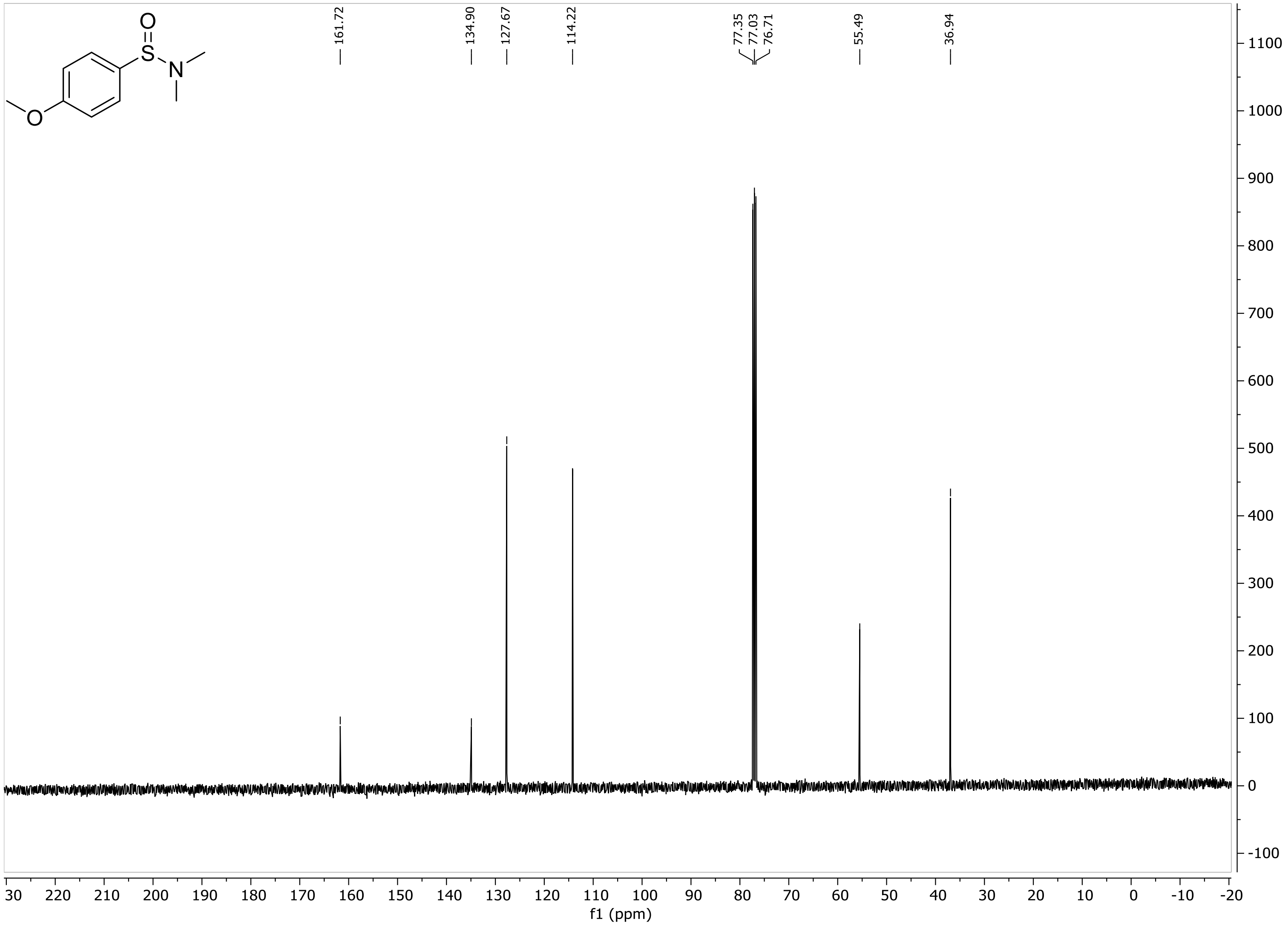

Figure S41. $^{13}C\{^{1}H\}$ NMR (101 MHz) of **2c** in $CDCl_3$.

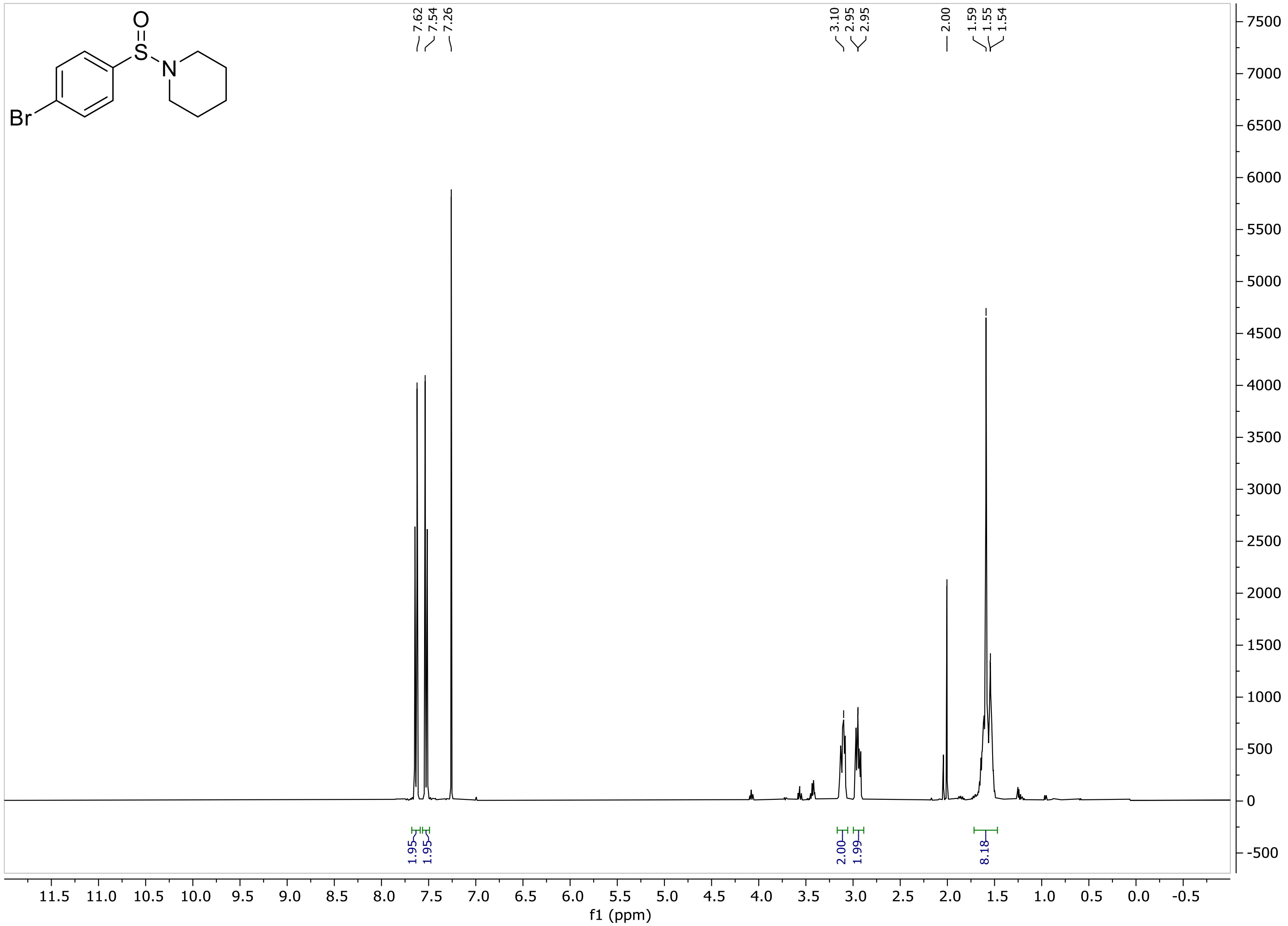

Figure S42. ^{1}H NMR (400 MHz) of **1d** in $CDCl_3$.

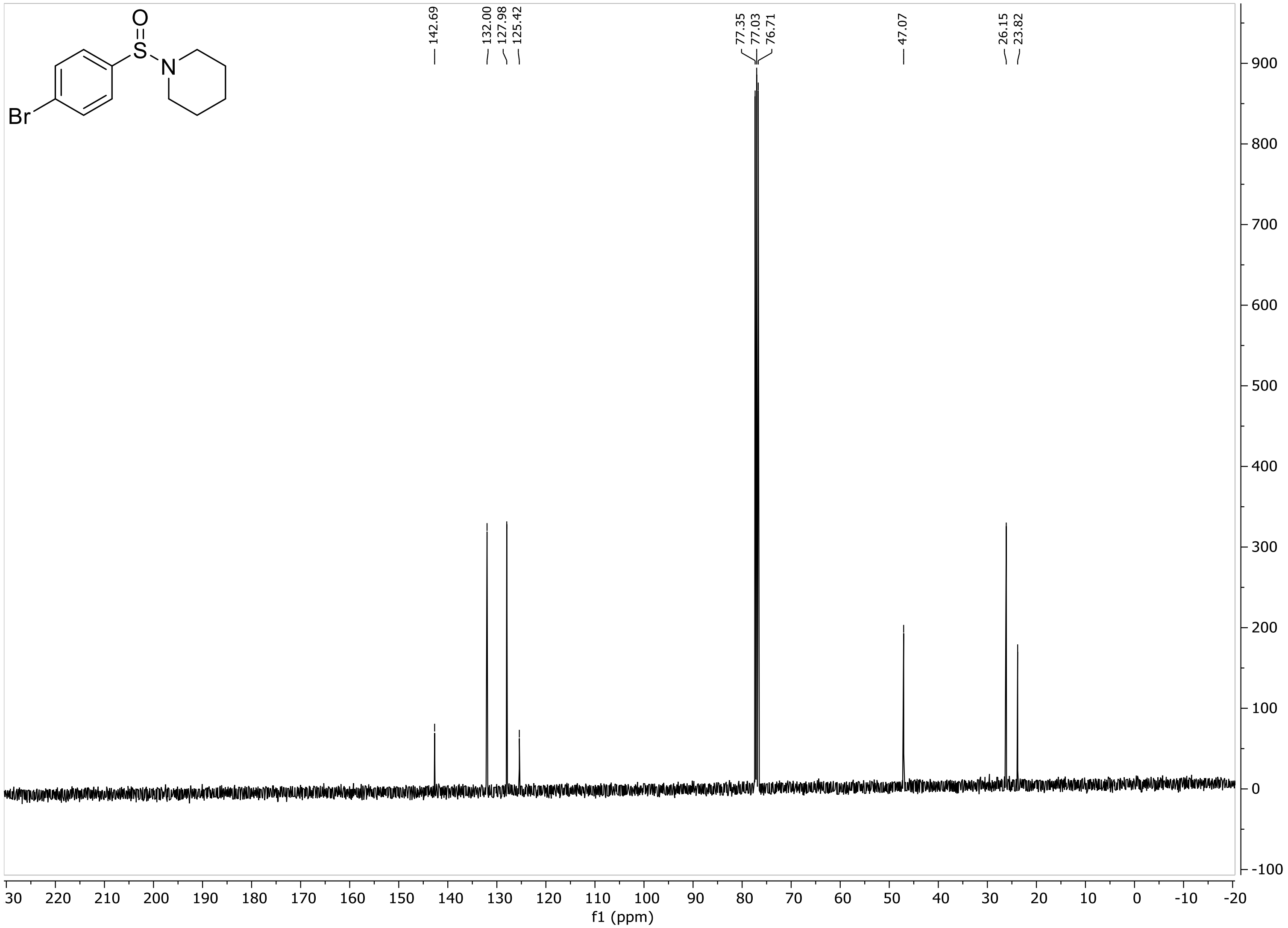

Figure S43. $^{13}C\{^{1}H\}$ NMR (101 MHz) of **1d** in $CDCl_3$.

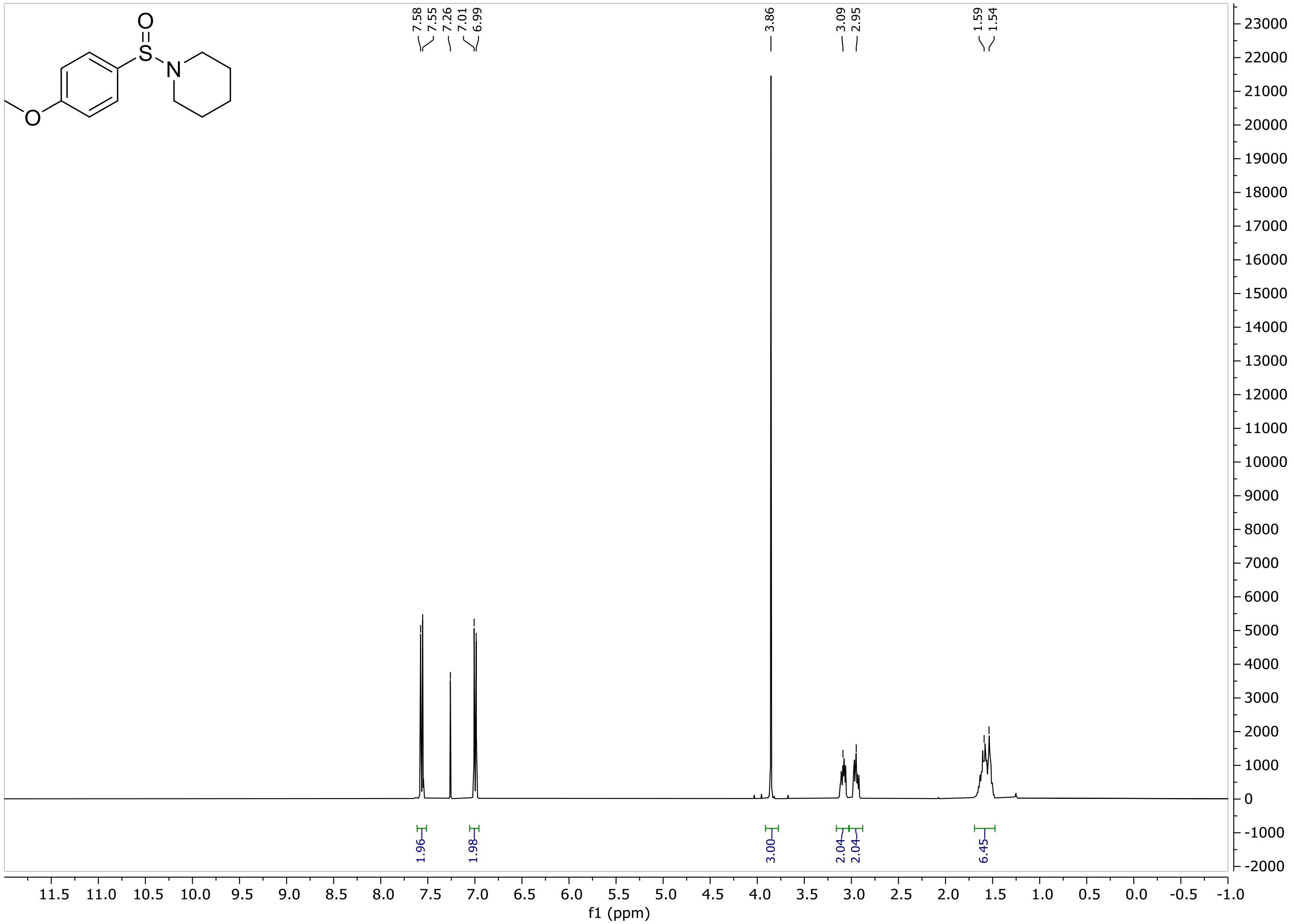

Figure S44. ^{1}H NMR (400 MHz) of **2d** in $CDCl_3$.

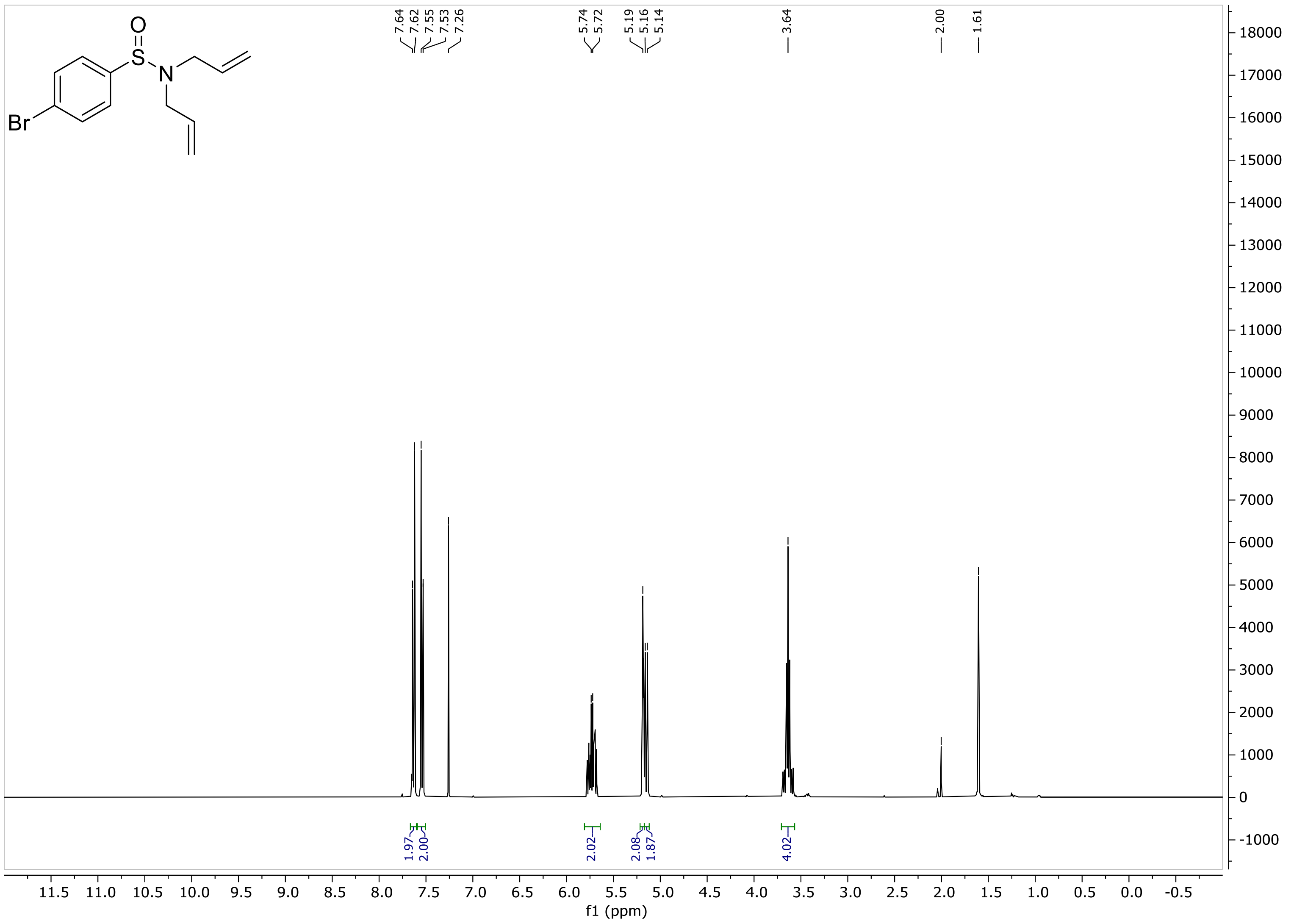

Figure S45. ^{1}H NMR (400 MHz) of **1e** in $CDCl_3$.

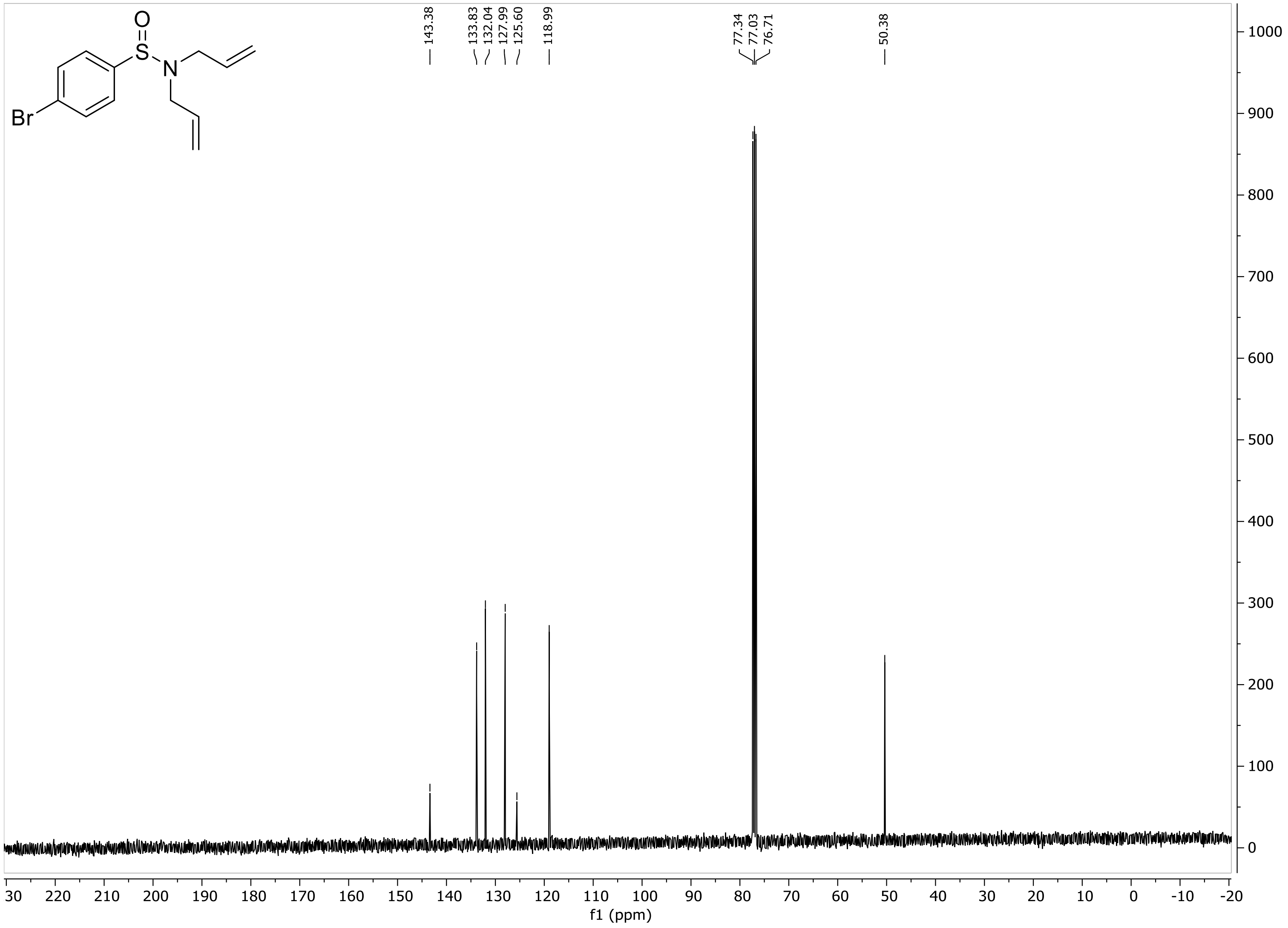

Figure S46. $^{13}C\{^{1}H\}$ NMR (101 MHz) of **1e** in $CDCl_3$.

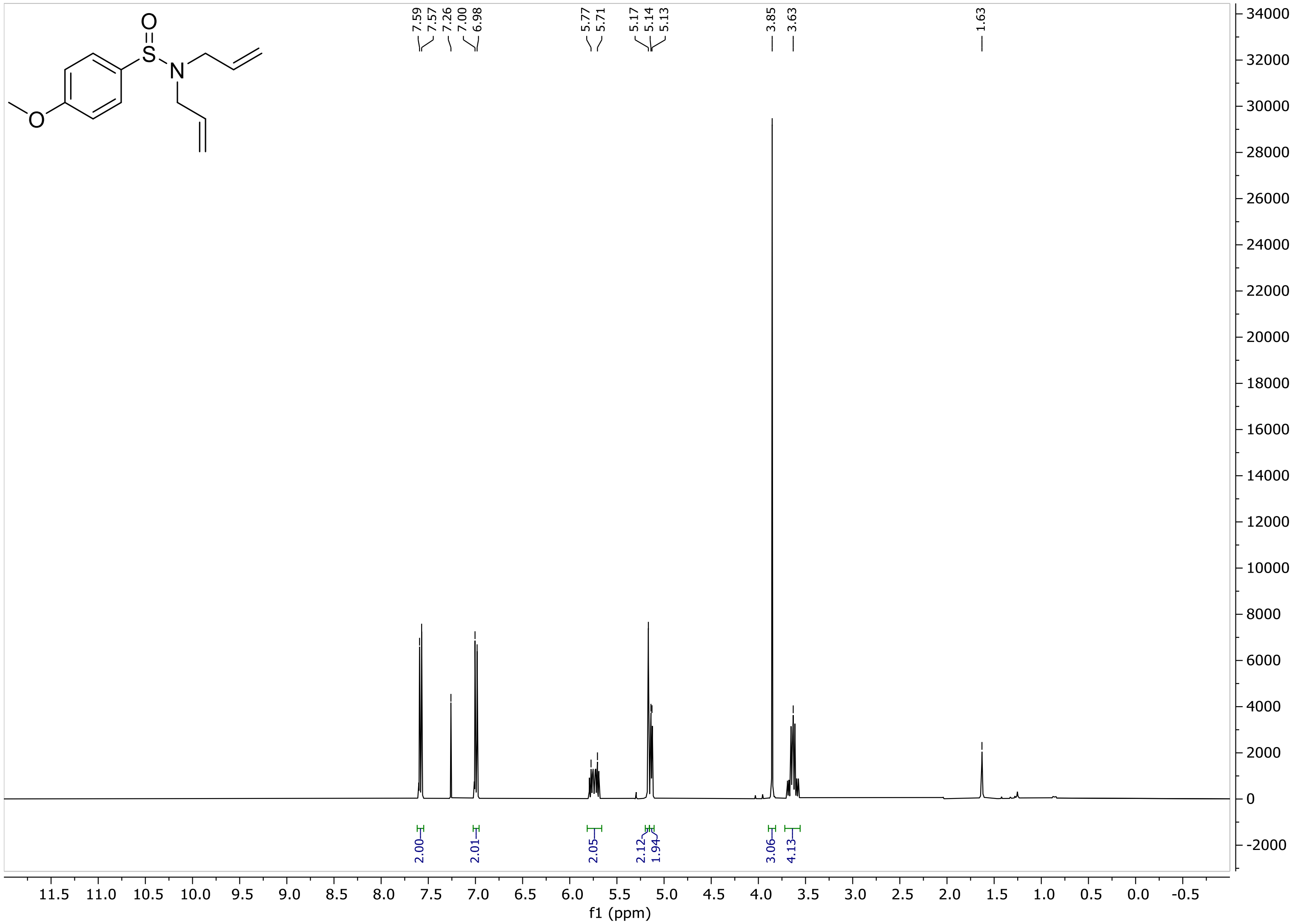

Figure S47. ^{1}H NMR (400 MHz) of **2e** in $CDCl_3$.

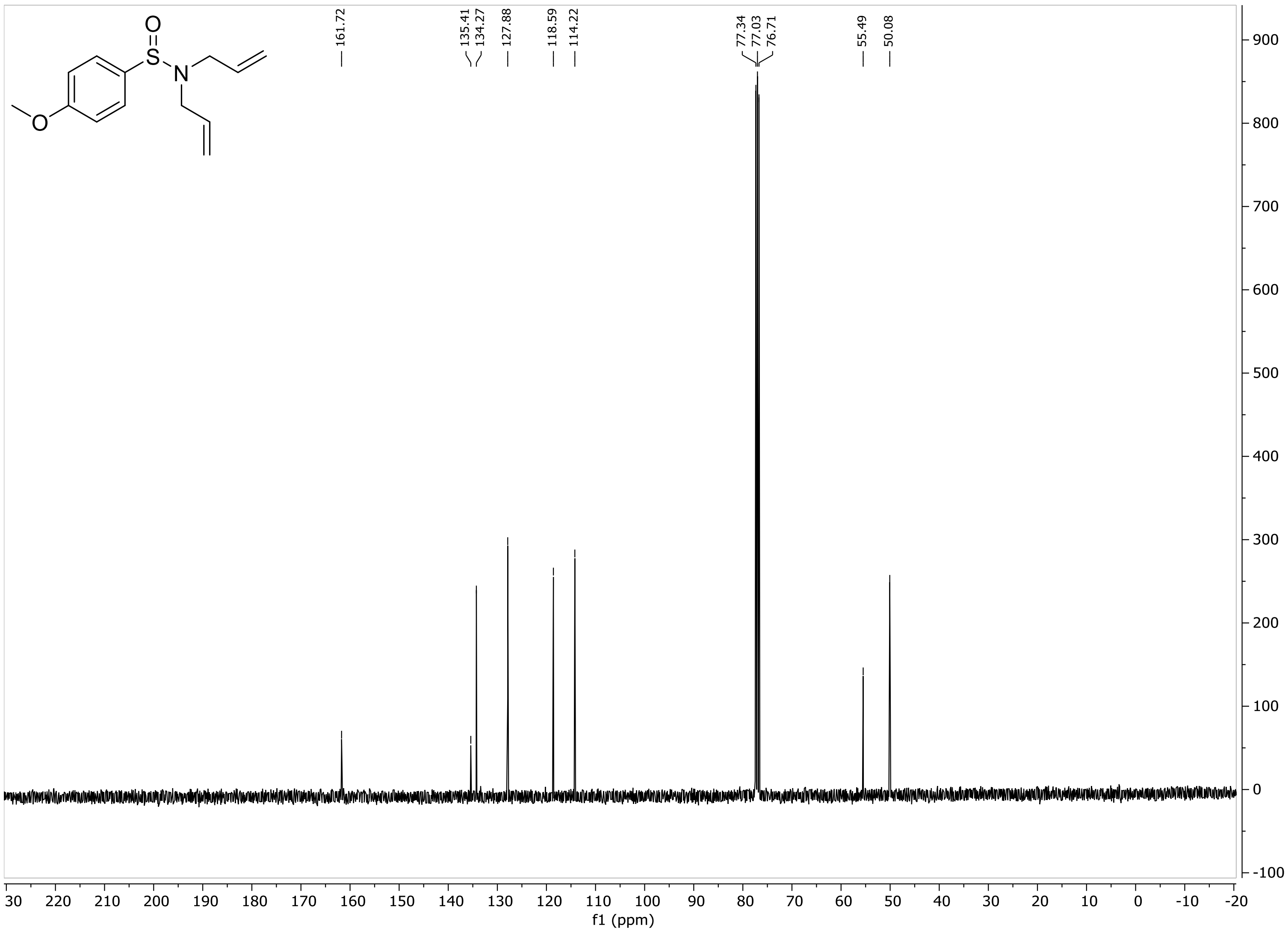

Figure S48. $^{13}C\{^{1}H\}$ NMR (101 MHz) of **2e** in $CDCl_3$.

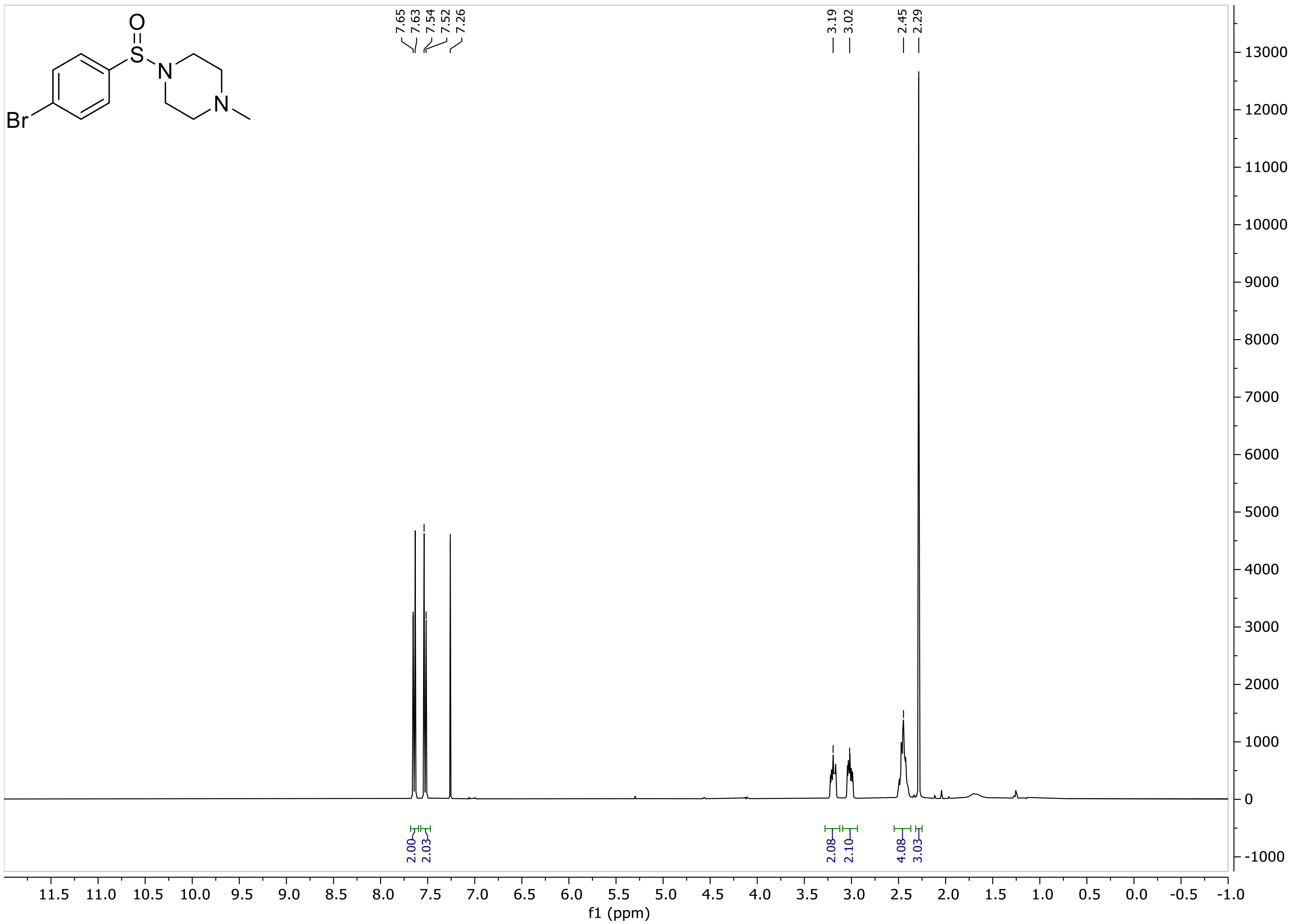

Figure S49. ^{1}H NMR (400 MHz) of **1f** in $CDCl_3$.

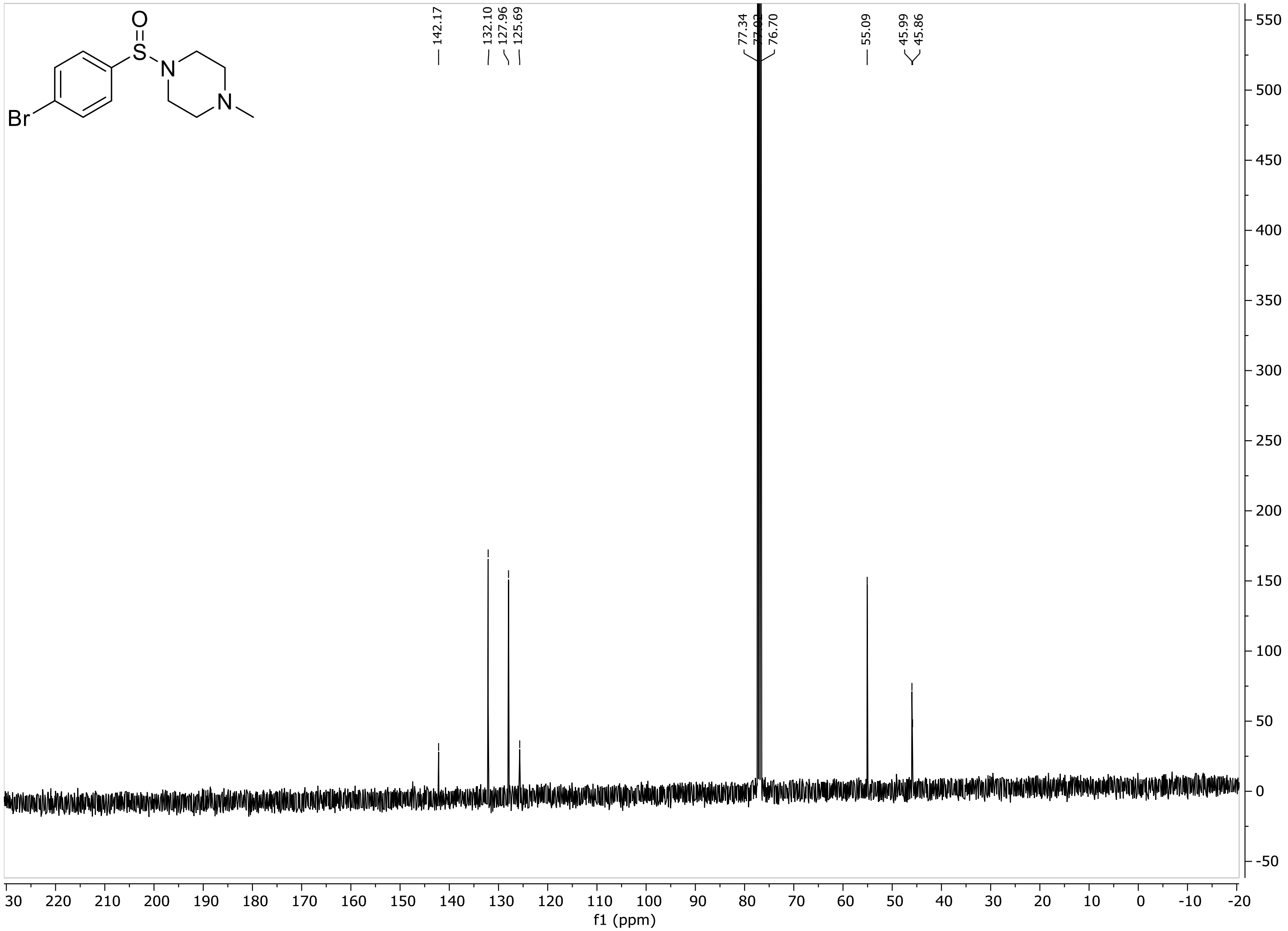

Figure S50. $^{13}C\{^{1}H\}$ NMR (101 MHz) of **1f** in $CDCl_3$.

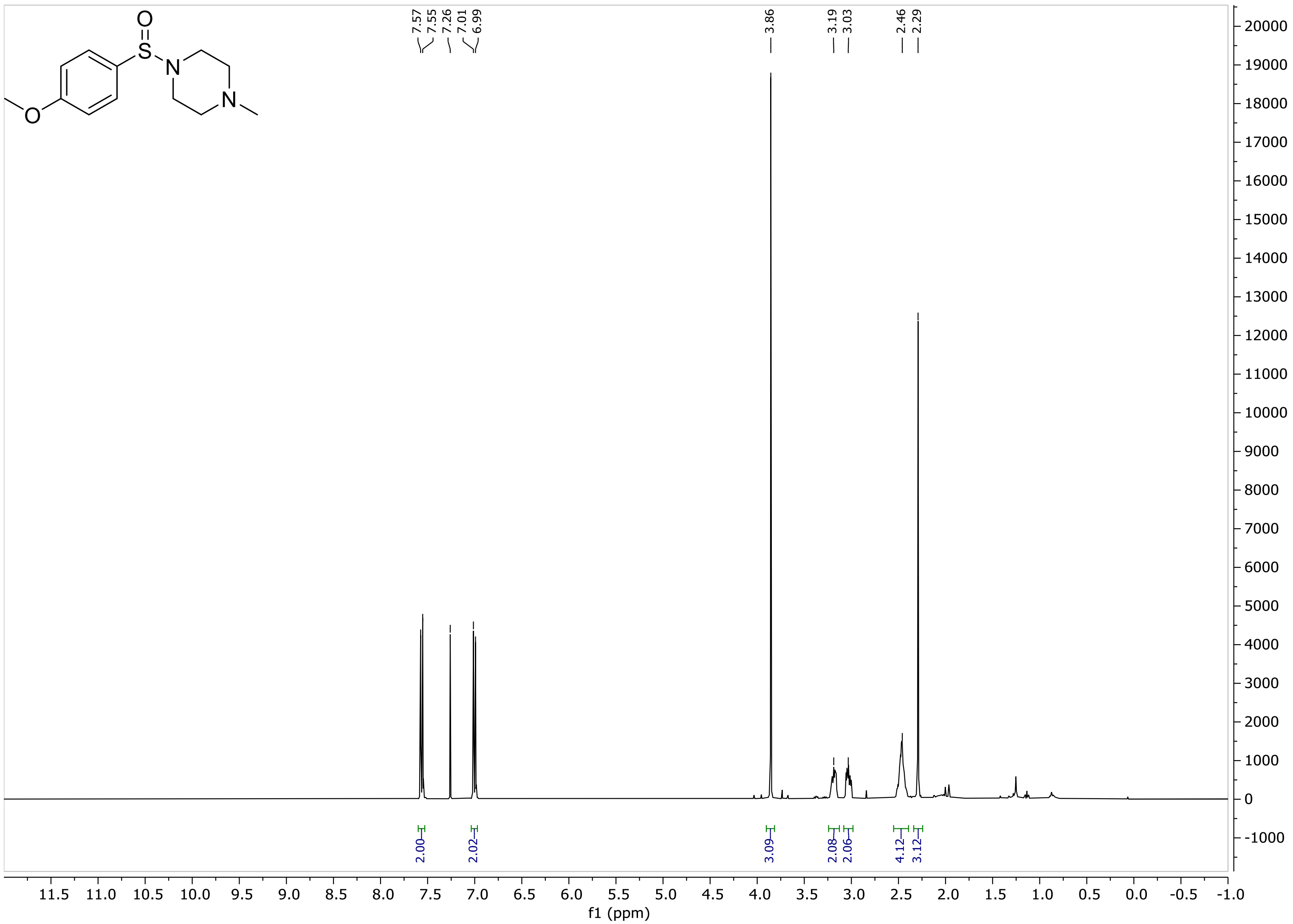

Figure S51. ^{1}H NMR (400 MHz) of **2f** in $CDCl_3$.

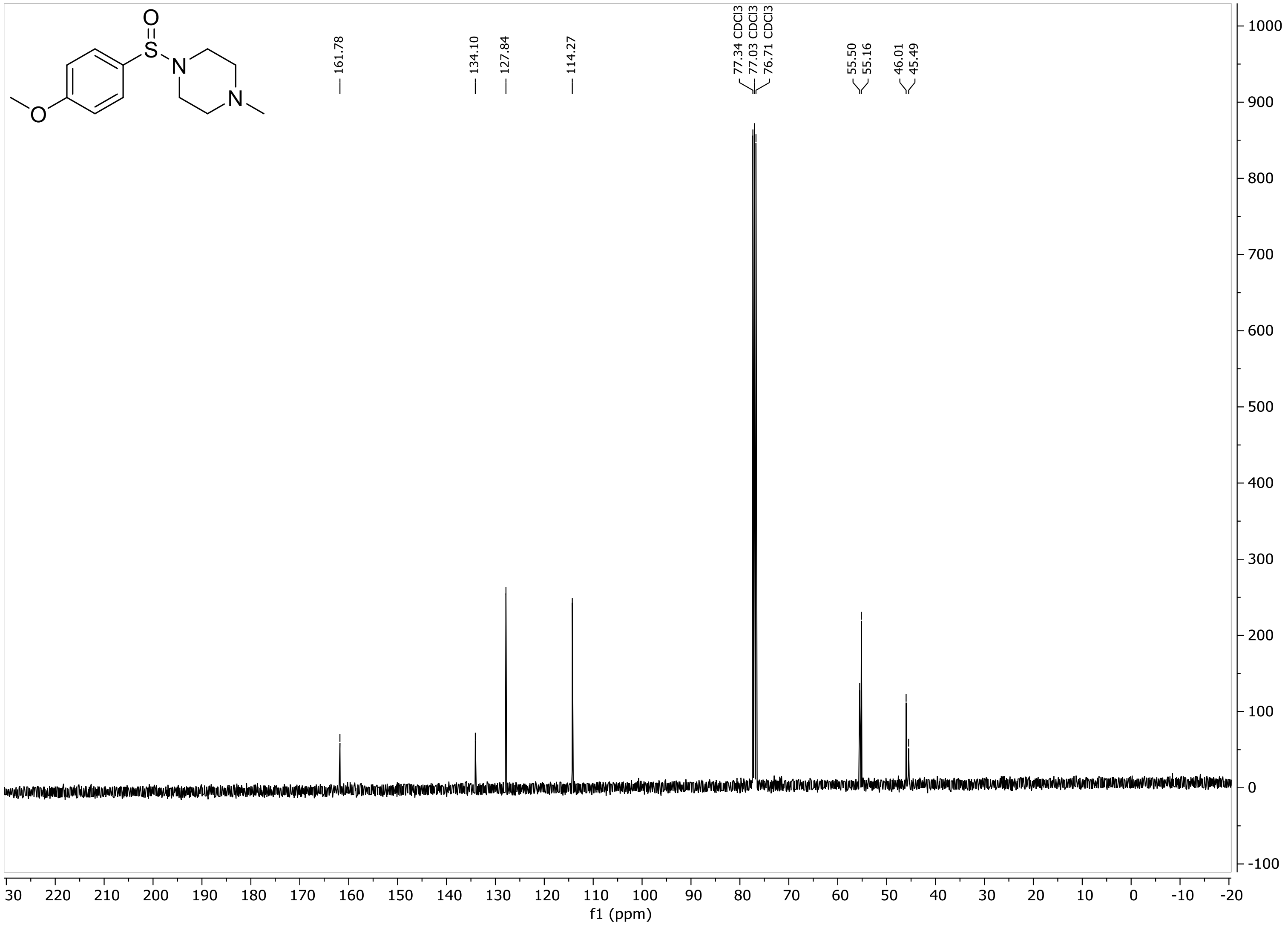

Figure S52. $^{13}C\{^{1}H\}$ NMR (101 MHz) of **2f** in $CDCl_3$.

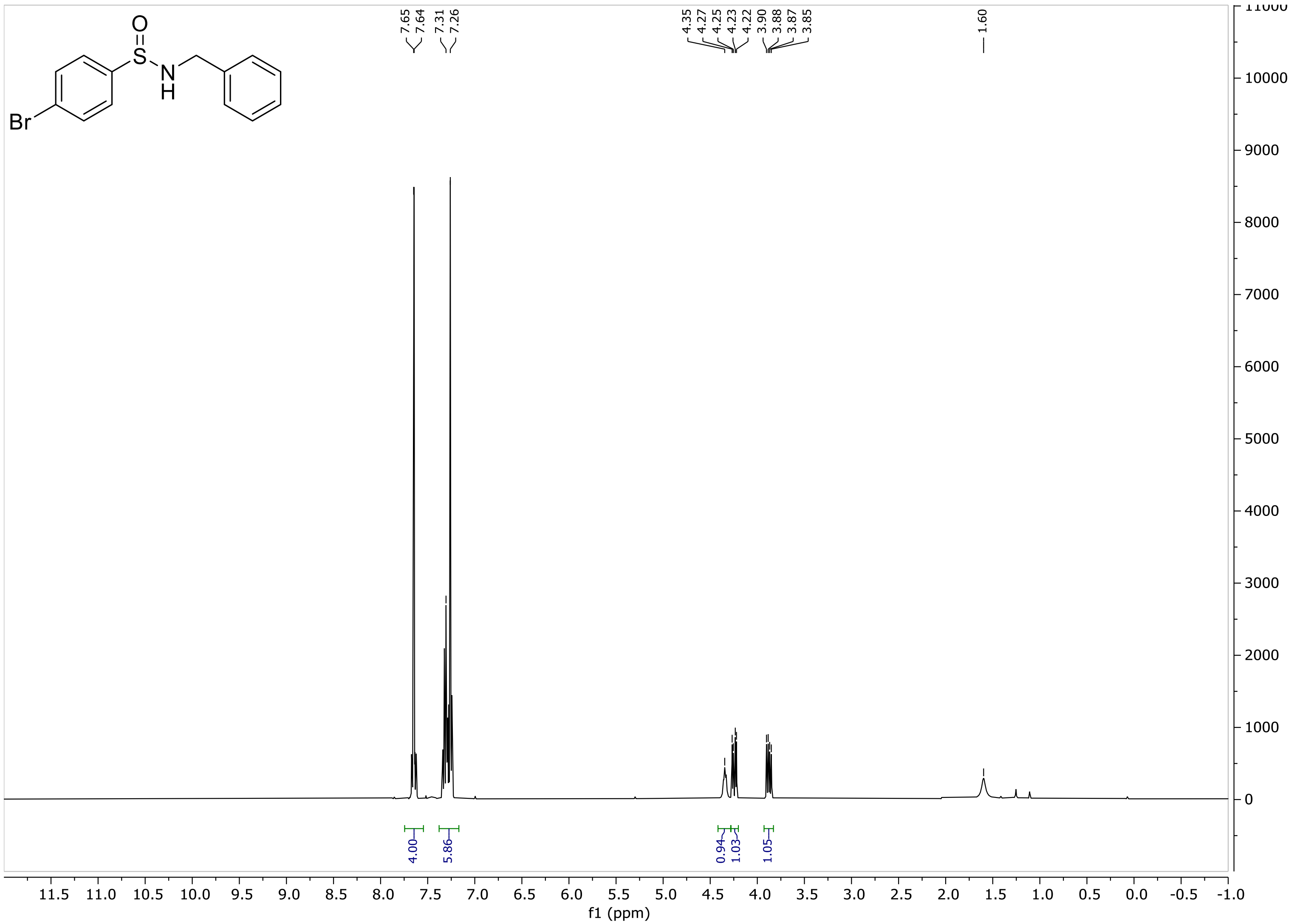

Figure S53. ^{1}H NMR (400 MHz) of **1g** in $CDCl_3$.

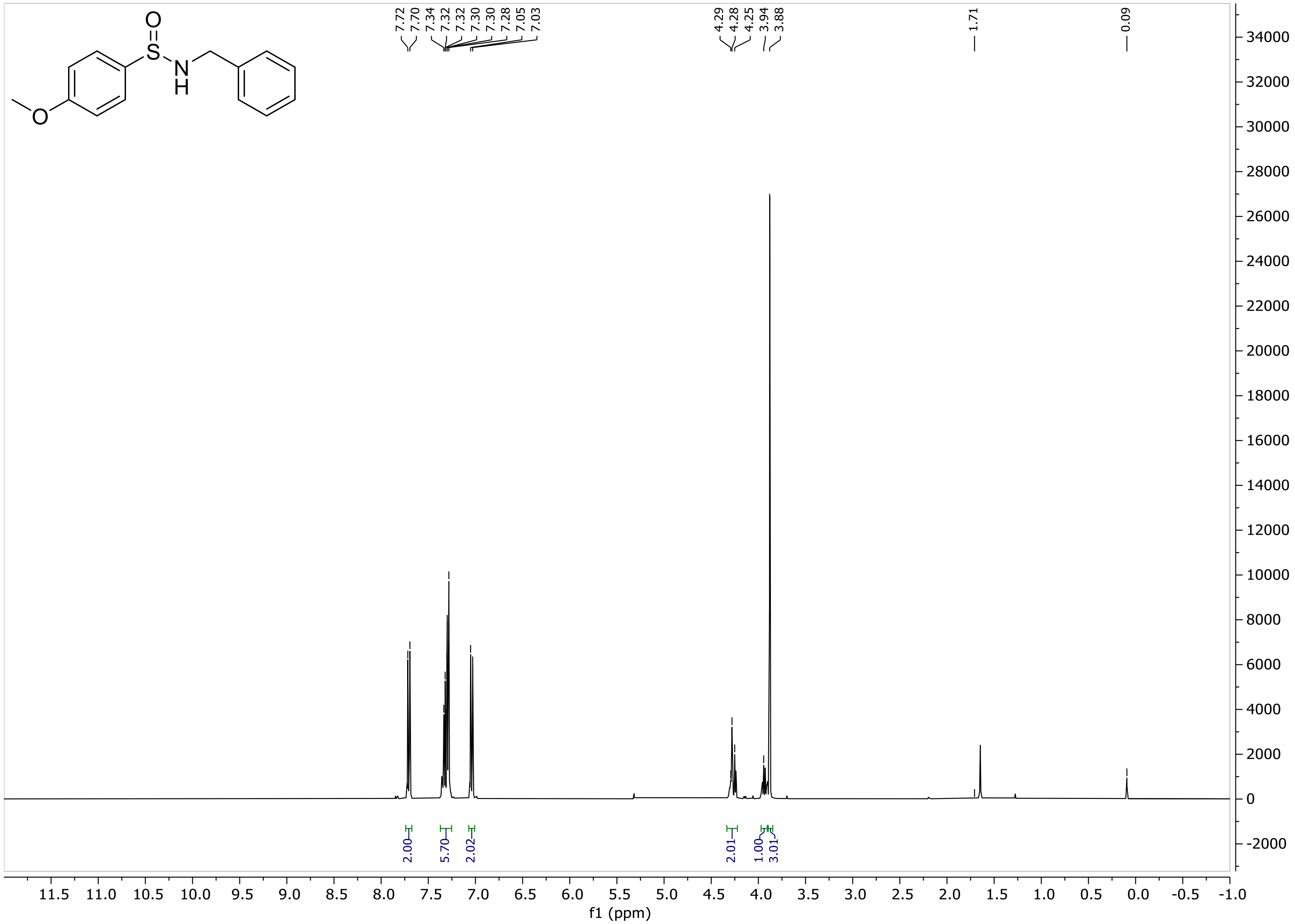

Figure S54. ^{1}H NMR (400 MHz) of **2g** in $CDCl_3$.

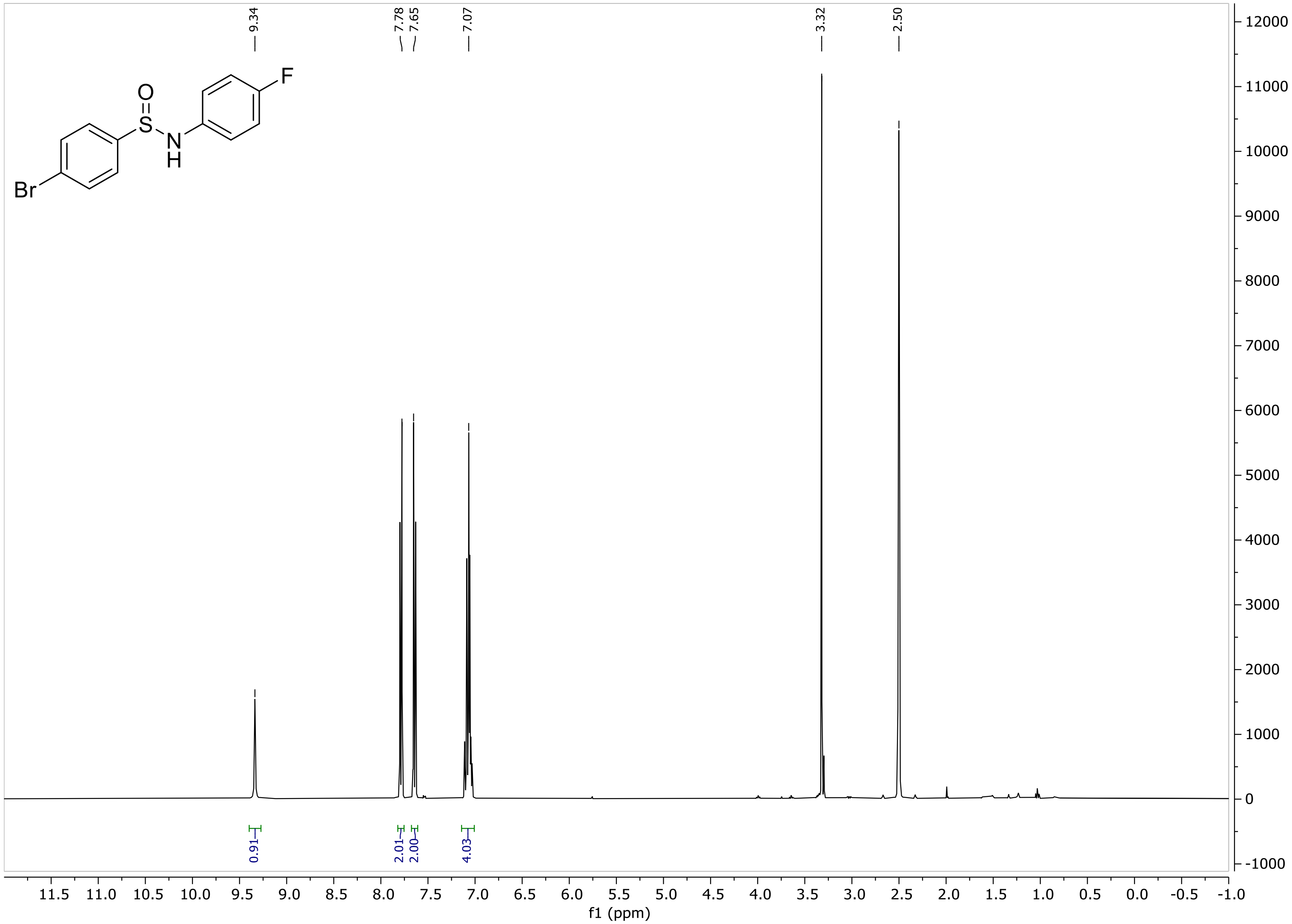

Figure S55. ^{1}H NMR (400 MHz) of **1h** in D_6-DMSO.

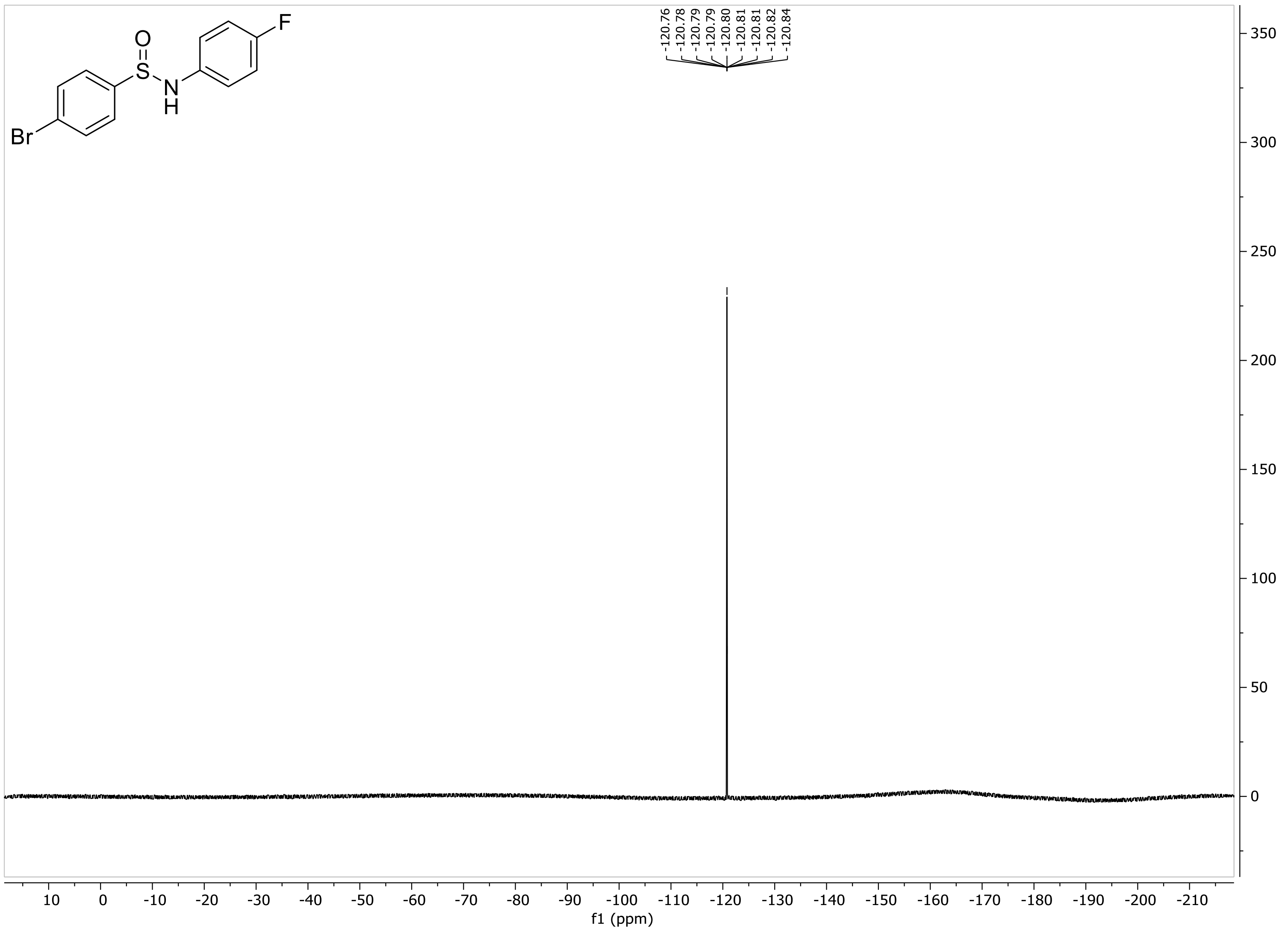

Figure S56. ^{19}F NMR (376 MHz) of **1h** in D_6-DMSO.

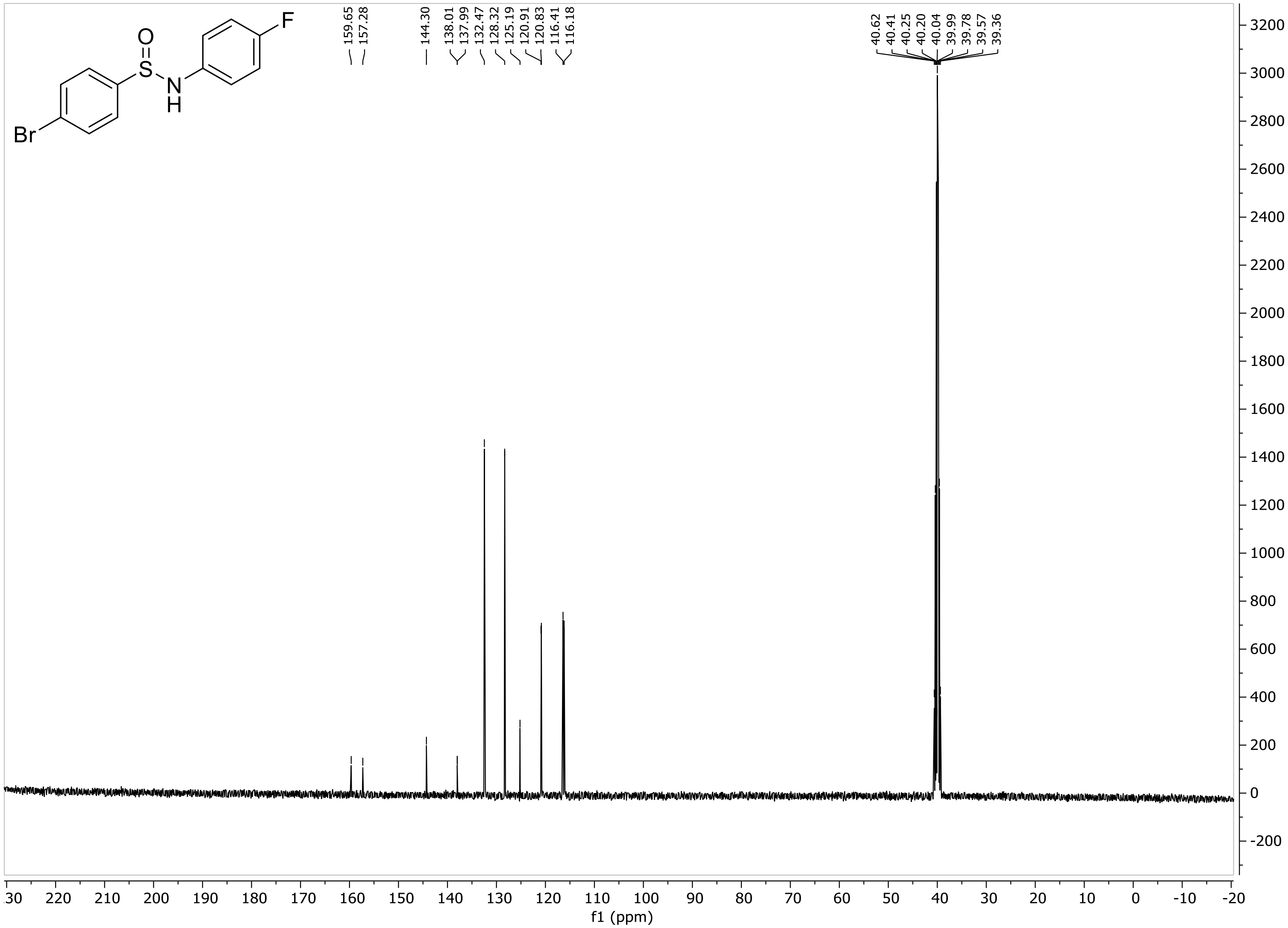

Figure S57. $^{13}C\{^{1}H\}$ NMR (101 MHz) of **1h** in D_6-DMSO.

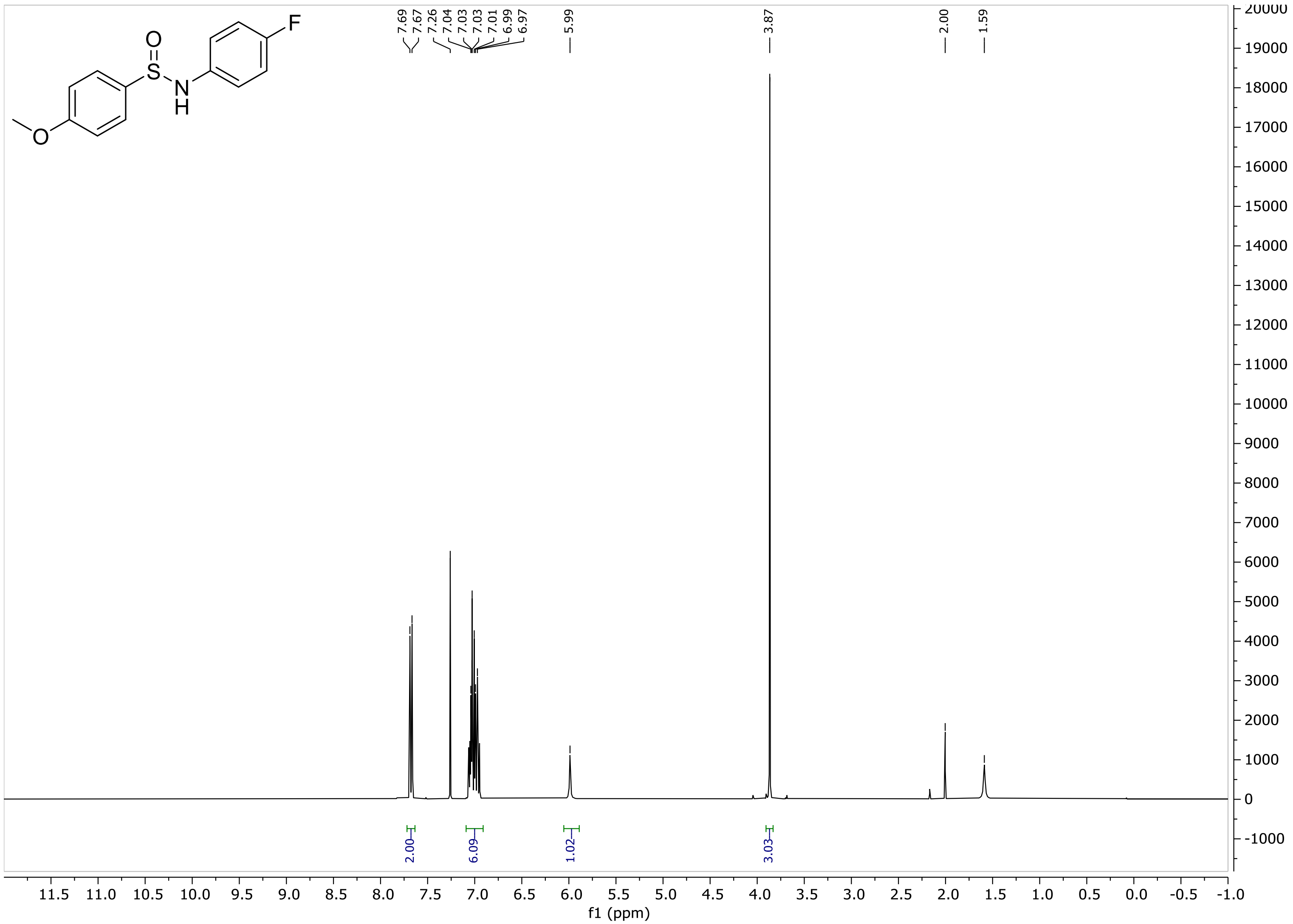

Figure S58. ^{1}H NMR (400 MHz) of **2h** in $CDCl_3$.

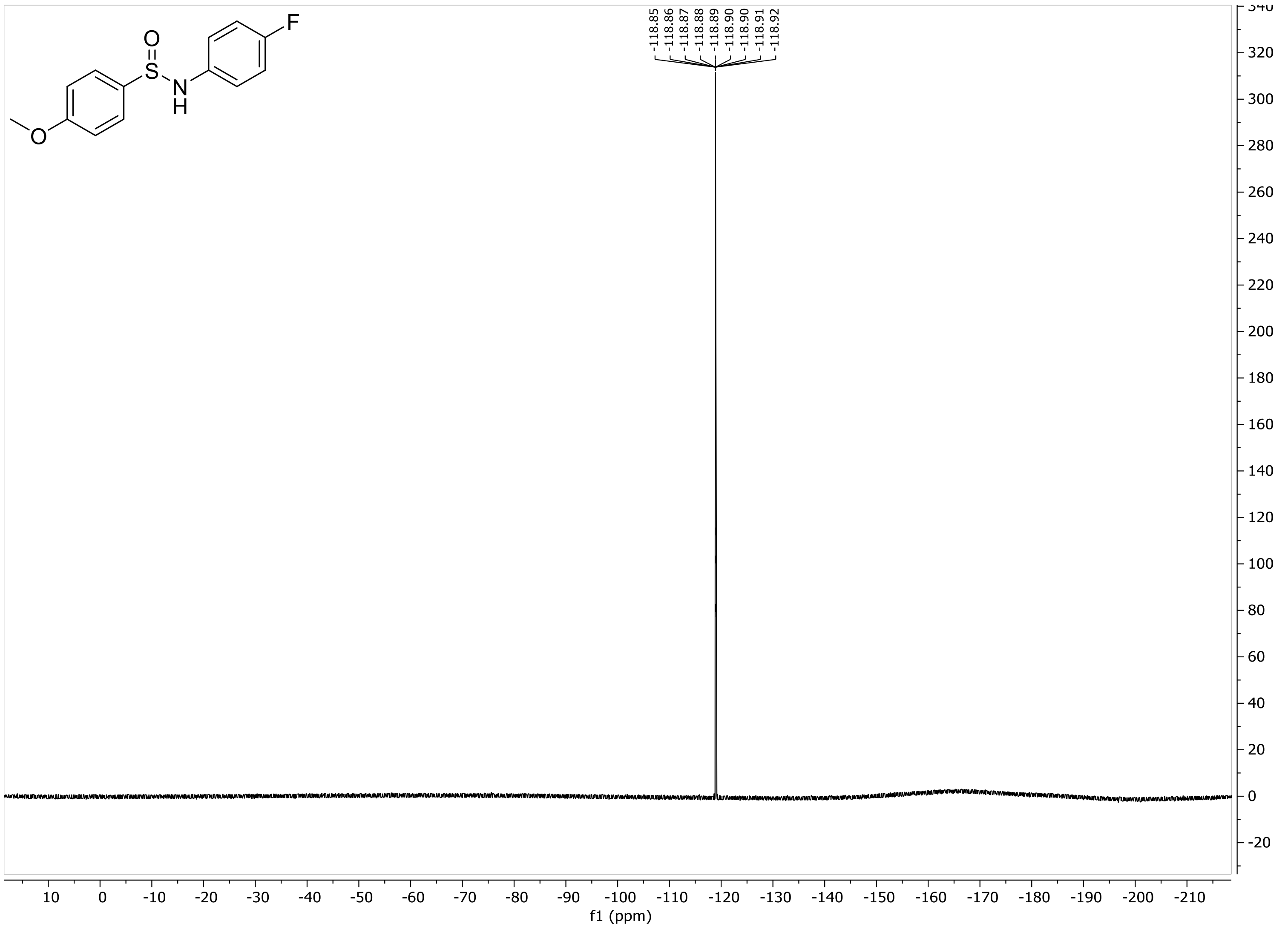

Figure S59.^{19}F NMR (376 MHz) of **2h** in $CDCl_3$.

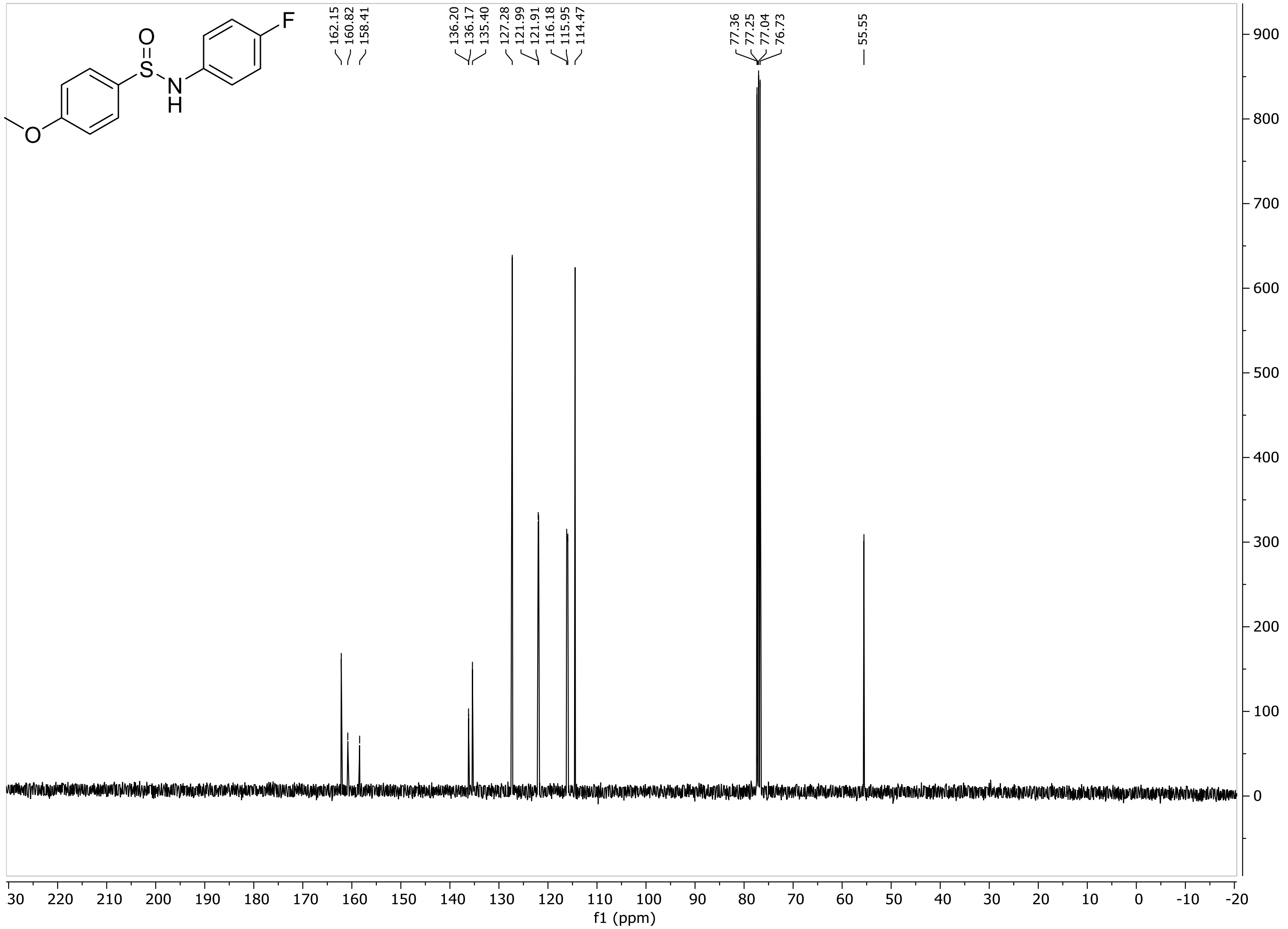

Figure S60. $^{13}C\{^1H\}$ NMR (101 MHz) of **2h** in $CDCl_3$.

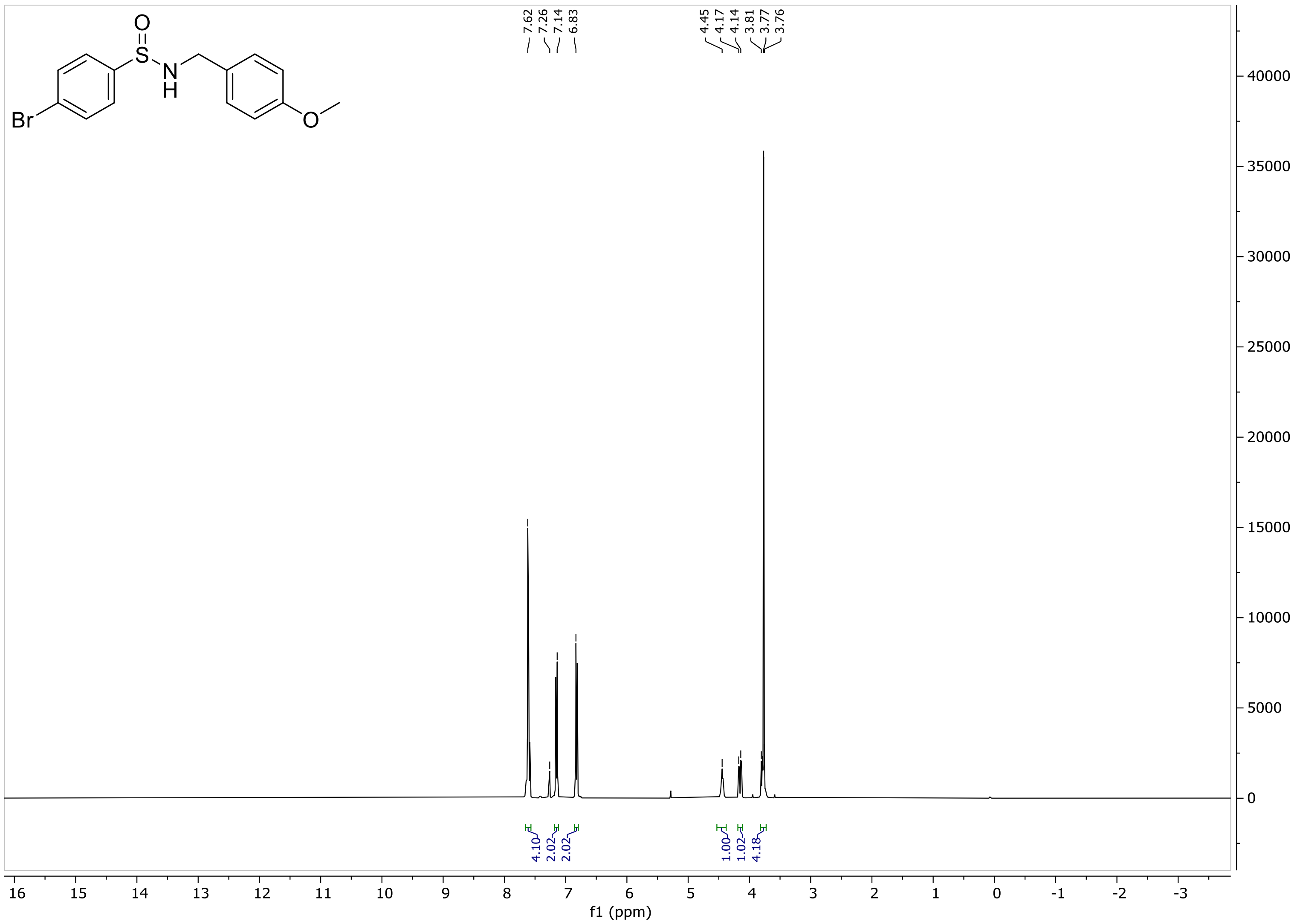

Figure S61. ^{1}H NMR (400Mhz) of **1j** in $CDCl_3$.

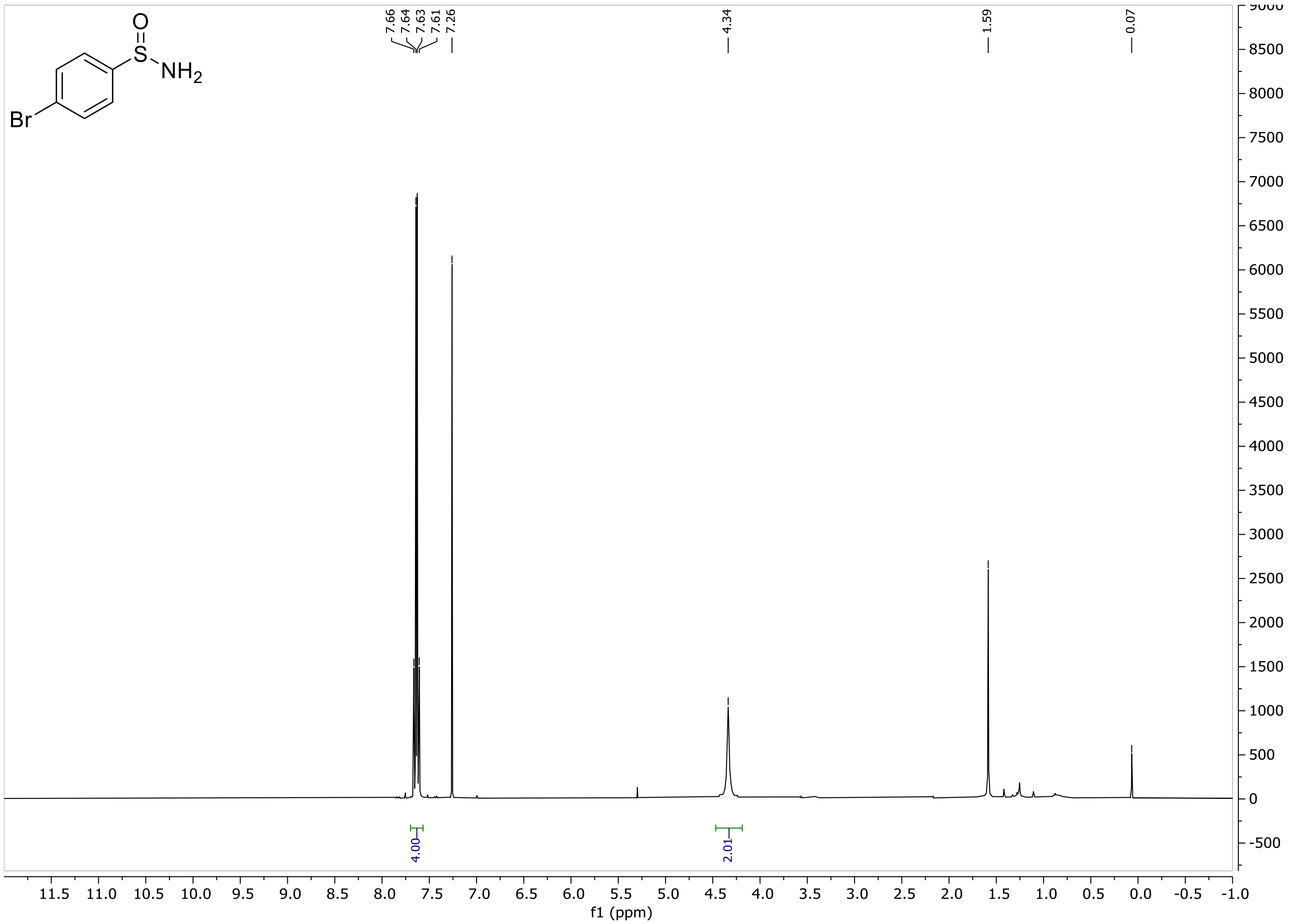

Figure S62 ^{1}H NMR (400Mhz) of **1k** in $CDCl_3$.

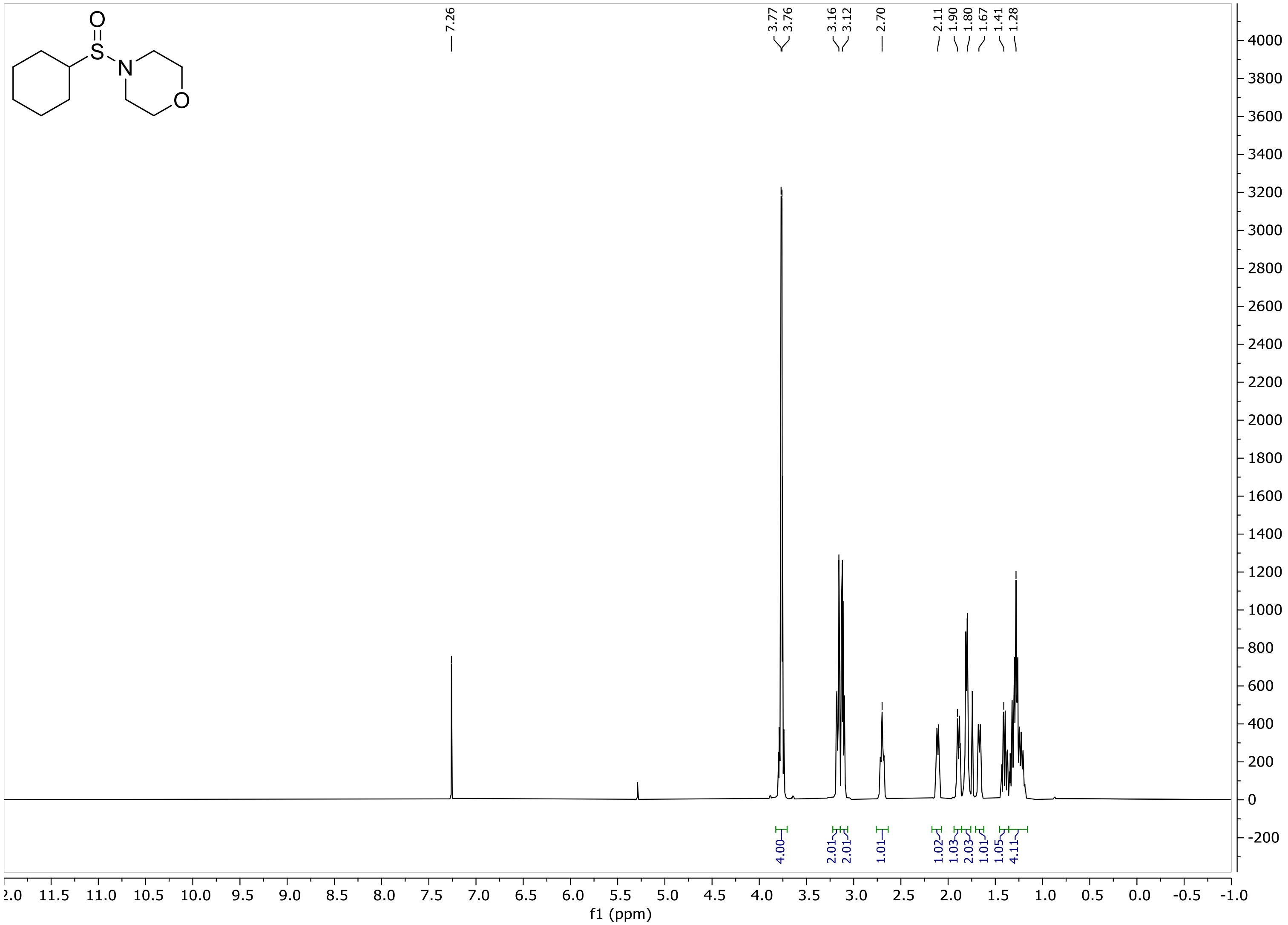

Figure S63. ^1H NMR (600 MHz) of **3a** in $CDCl_3$.

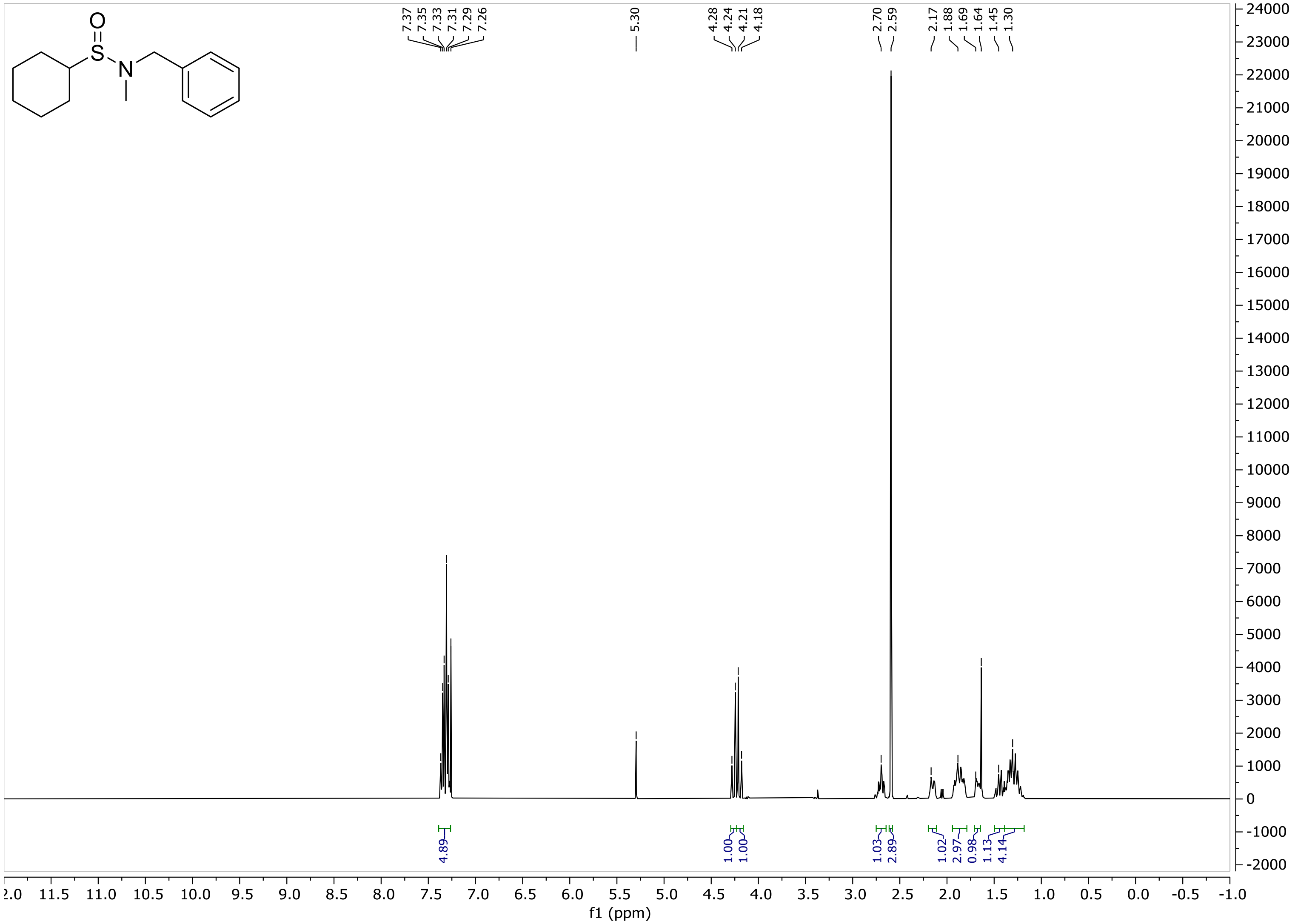

Figure S64. ^{1}H NMR (400 MHz) of **3b** in $CDCl_3$.

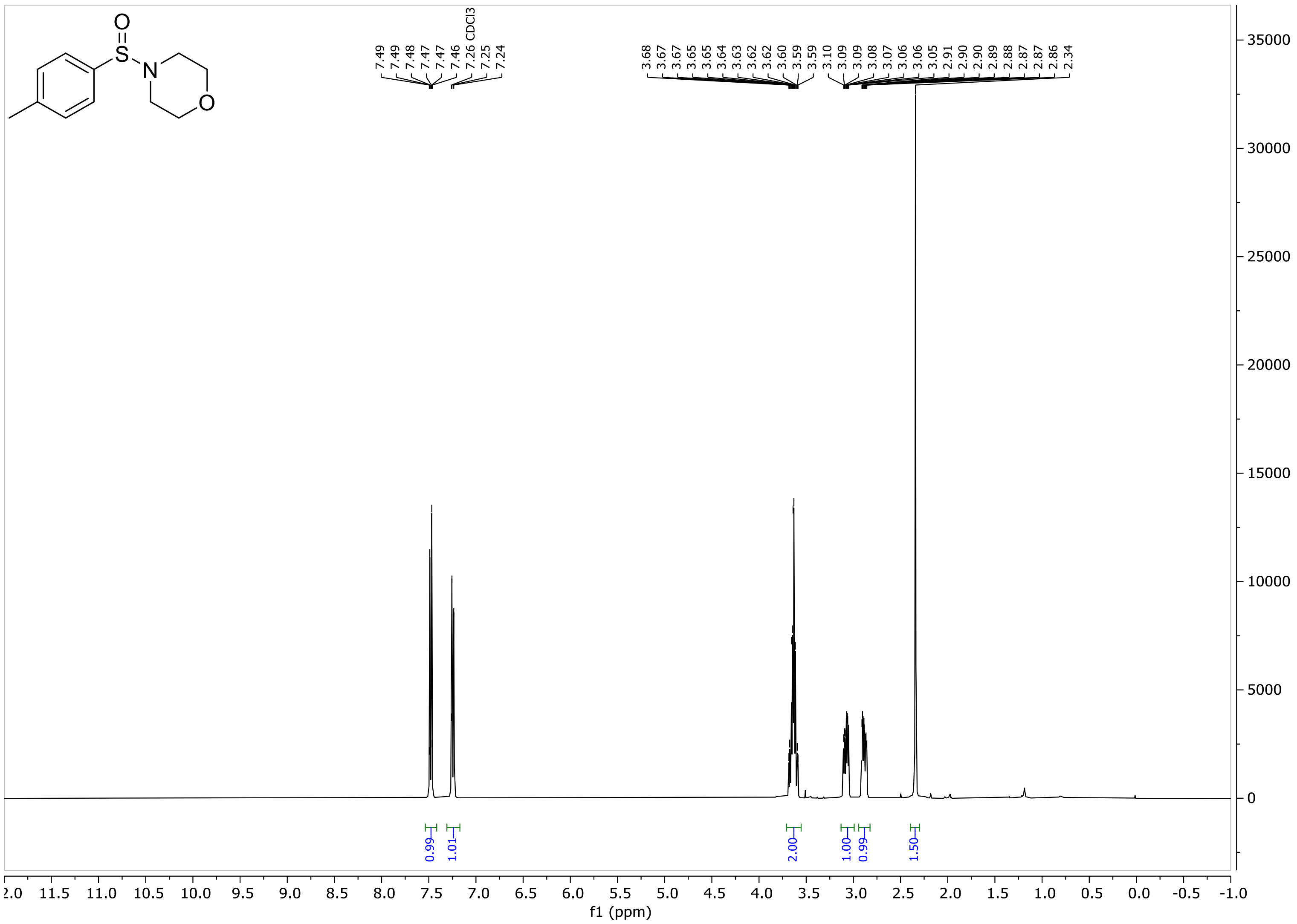

Figure S65.. ^{1}H NMR (400 MHz) of **4a** in $CDCl_3$.

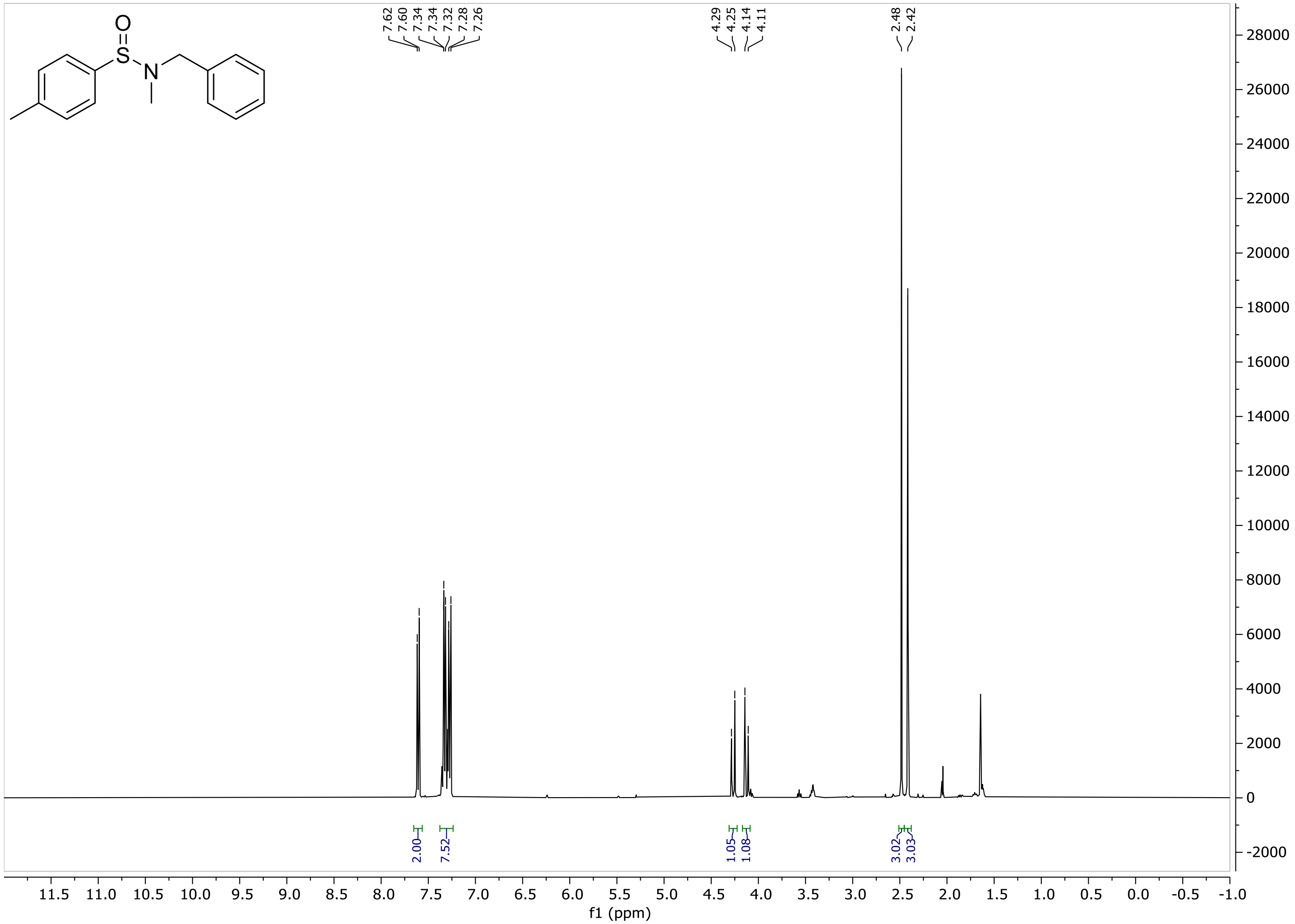

Figure S66. ^{1}H NMR (400 MHz) of **4b** in $CDCl_3$.

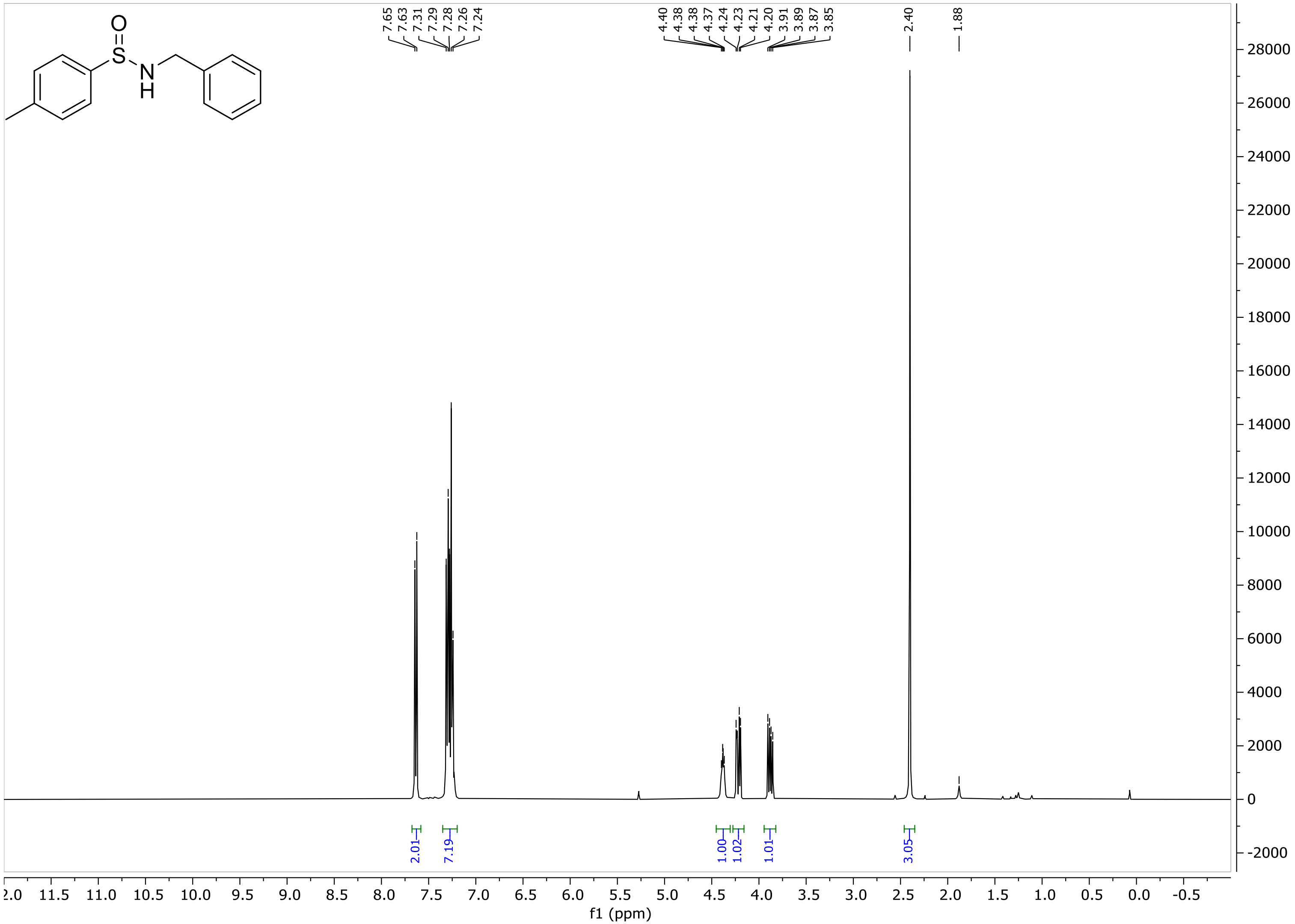

Figure S67. ^{1}H NMR (400 MHz) of **4g** in $CDCl_3$.

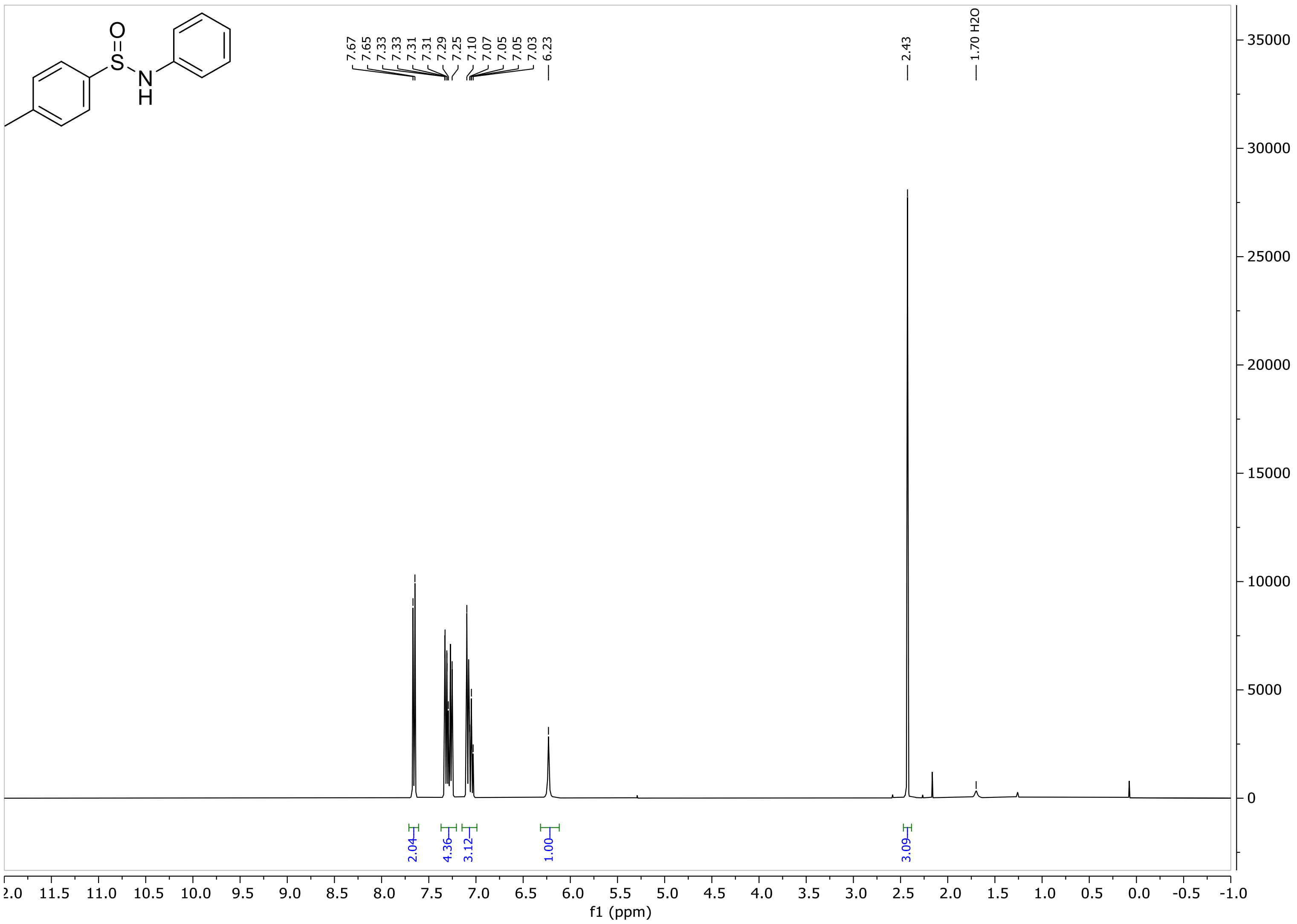

Figure S68. ^{1}H NMR (400 MHz) of **3i** in $CDCl_3$.

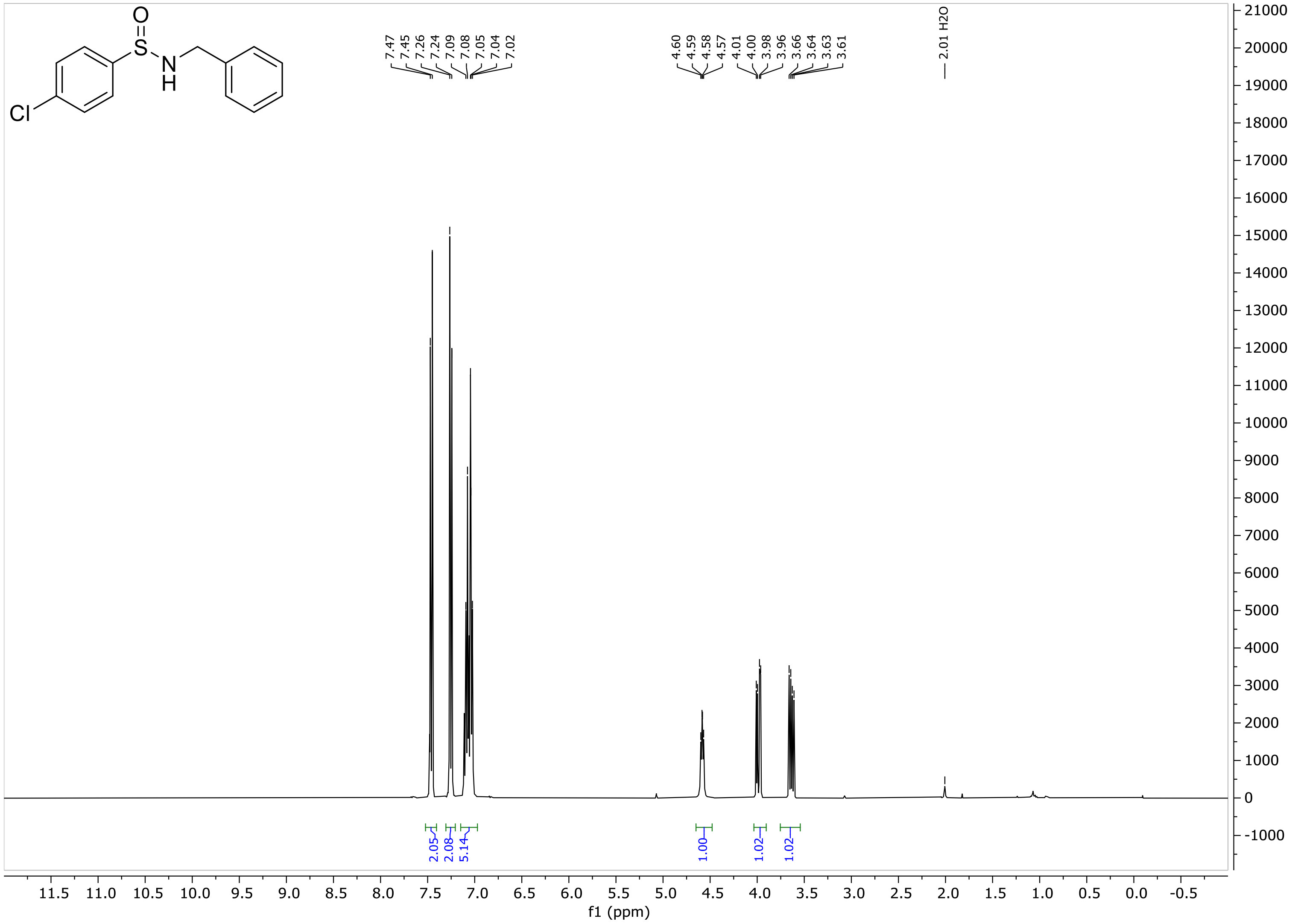

Figure S69. ^{1}H NMR (400MHz) of **6g** in $CDCl_3$.

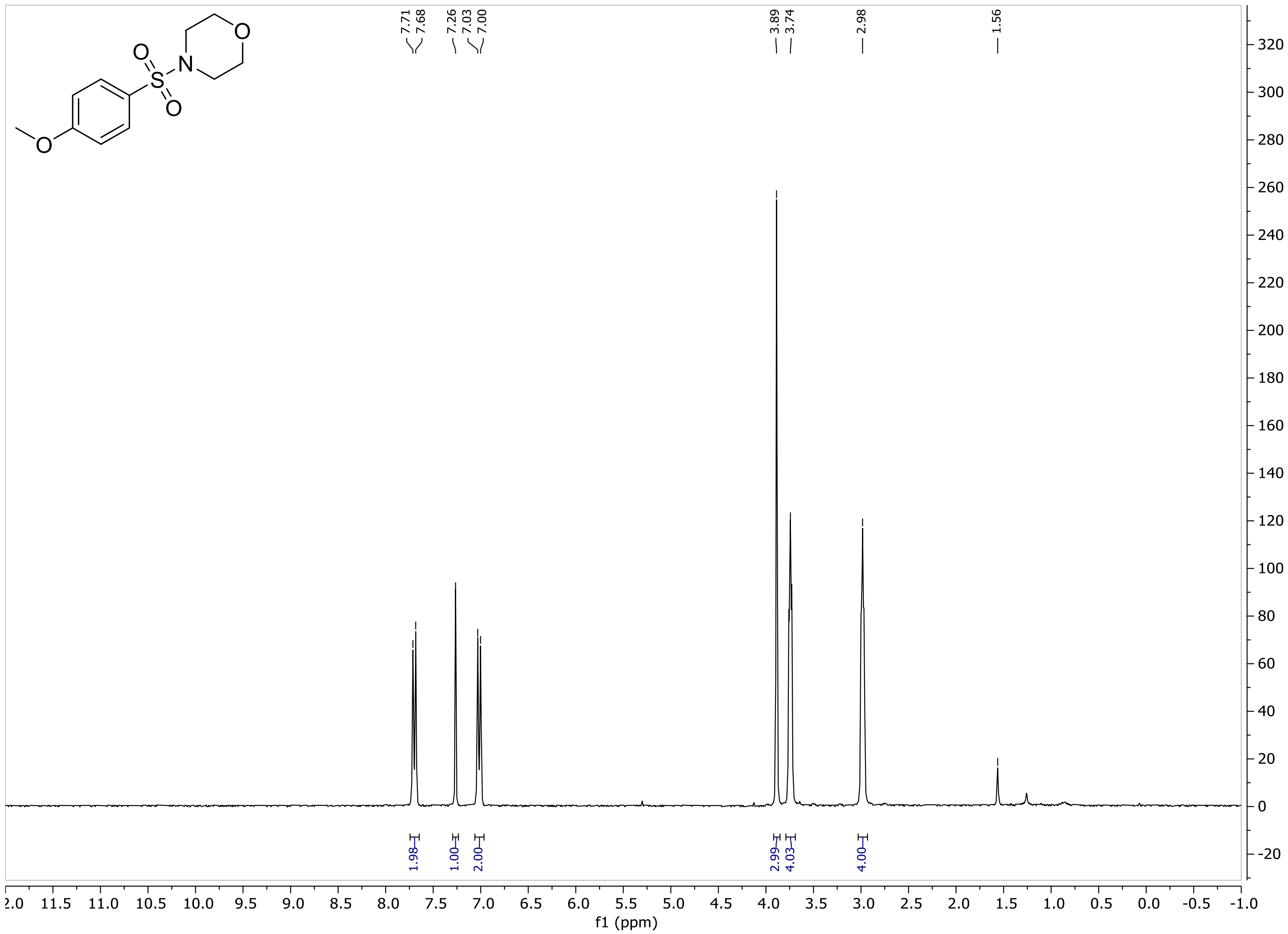

Figure S70. ^{1}H NMR (300 MHz) of **5a** in $CDCl_3$.

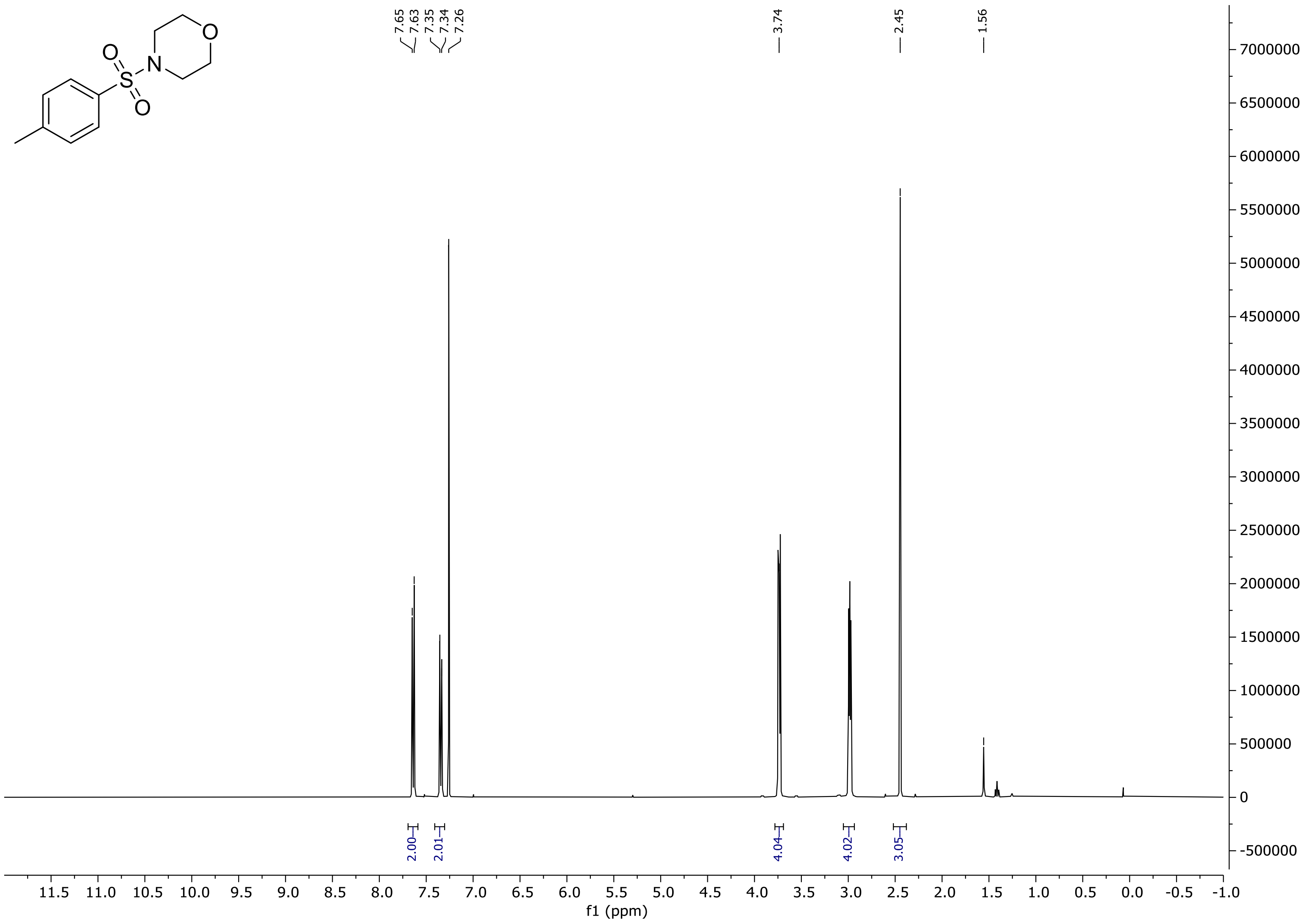

Figure S72. ^{1}H NMR (400 MHz) of **5b** in $CDCl_3$.

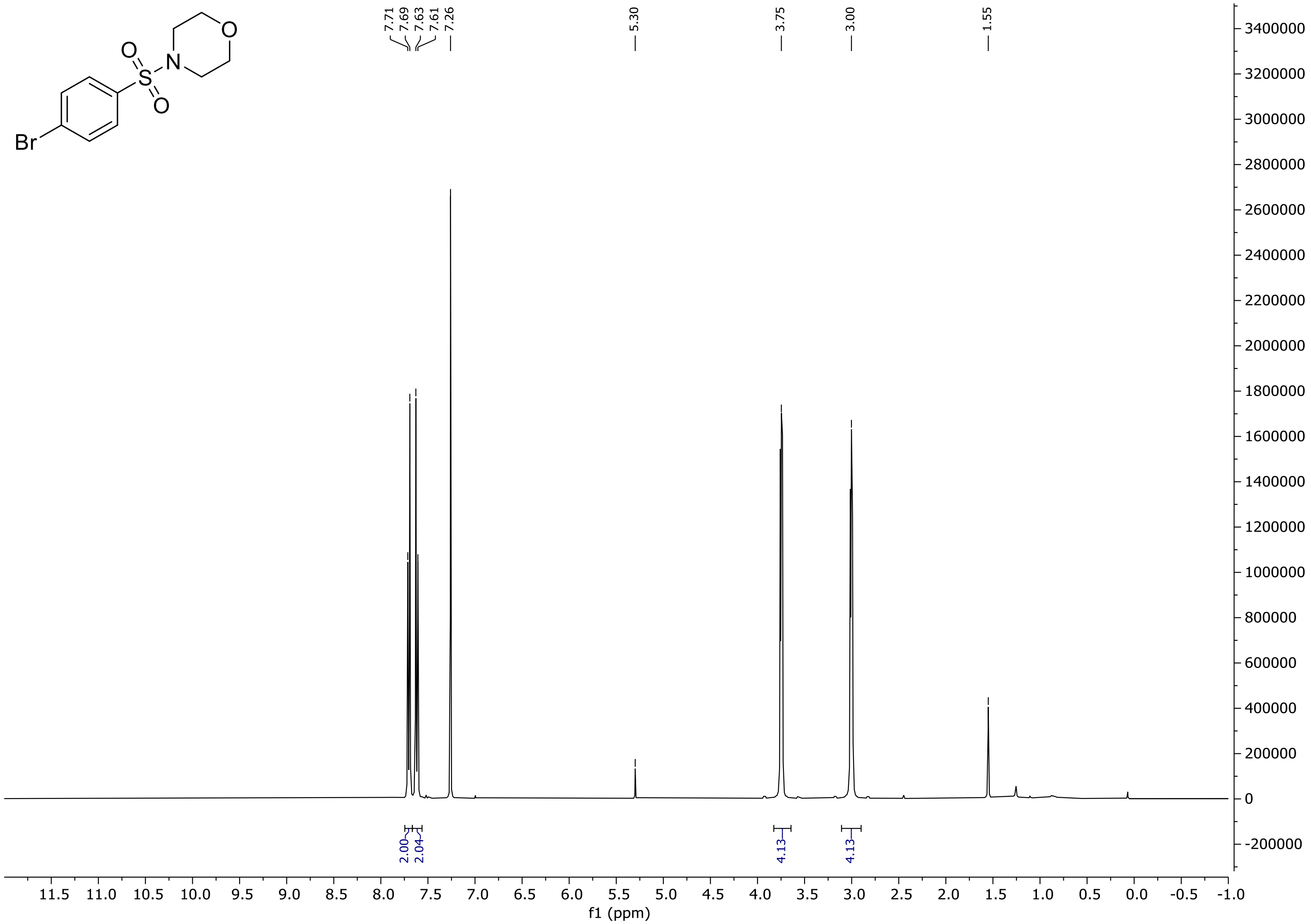

Figure S73. ^{1}H NMR (400 MHz) of **5c** in $CDCl_3$.

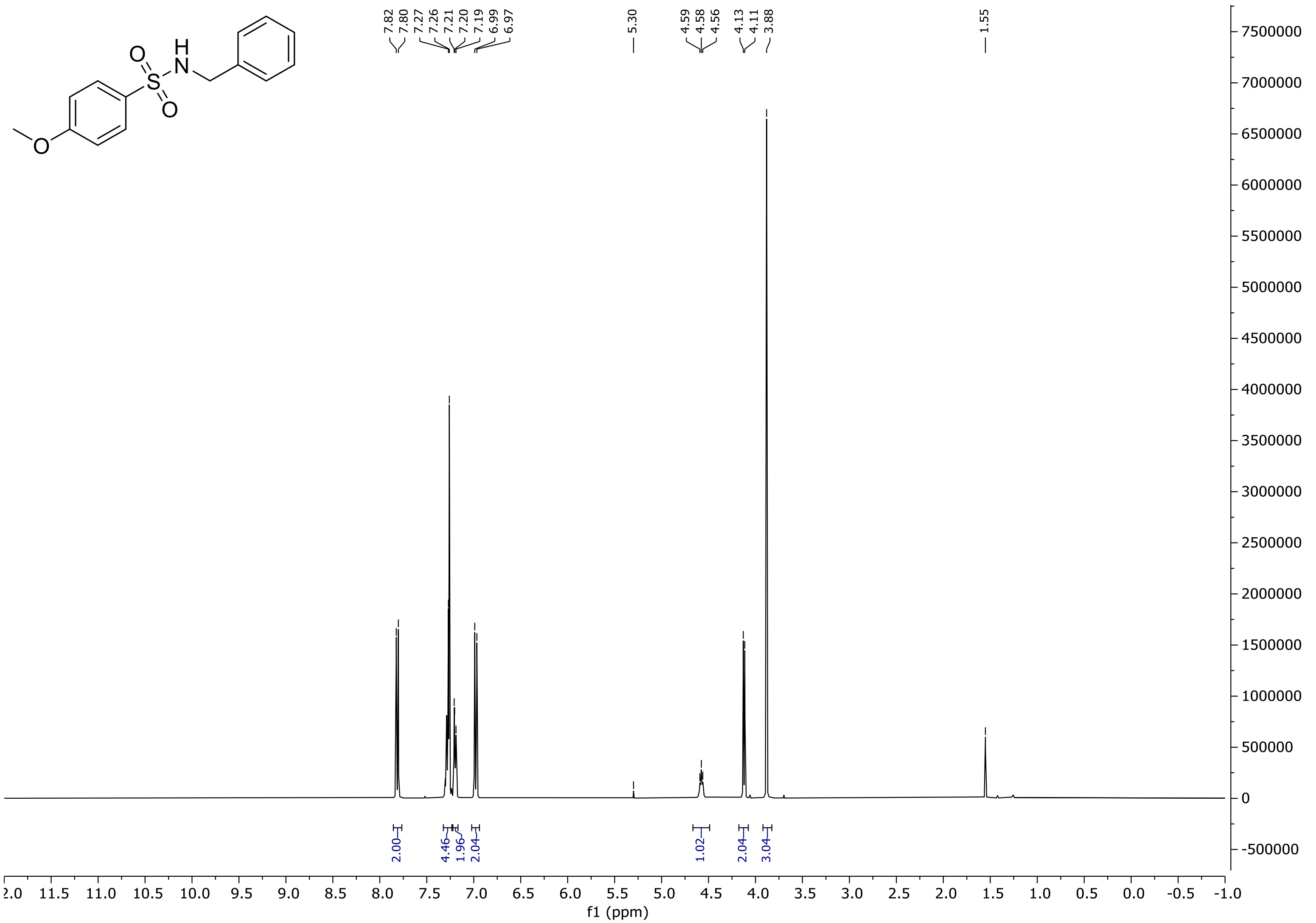

Figure S74. ^{1}H NMR (400 MHz) of **5d** in $CDCl_3$.

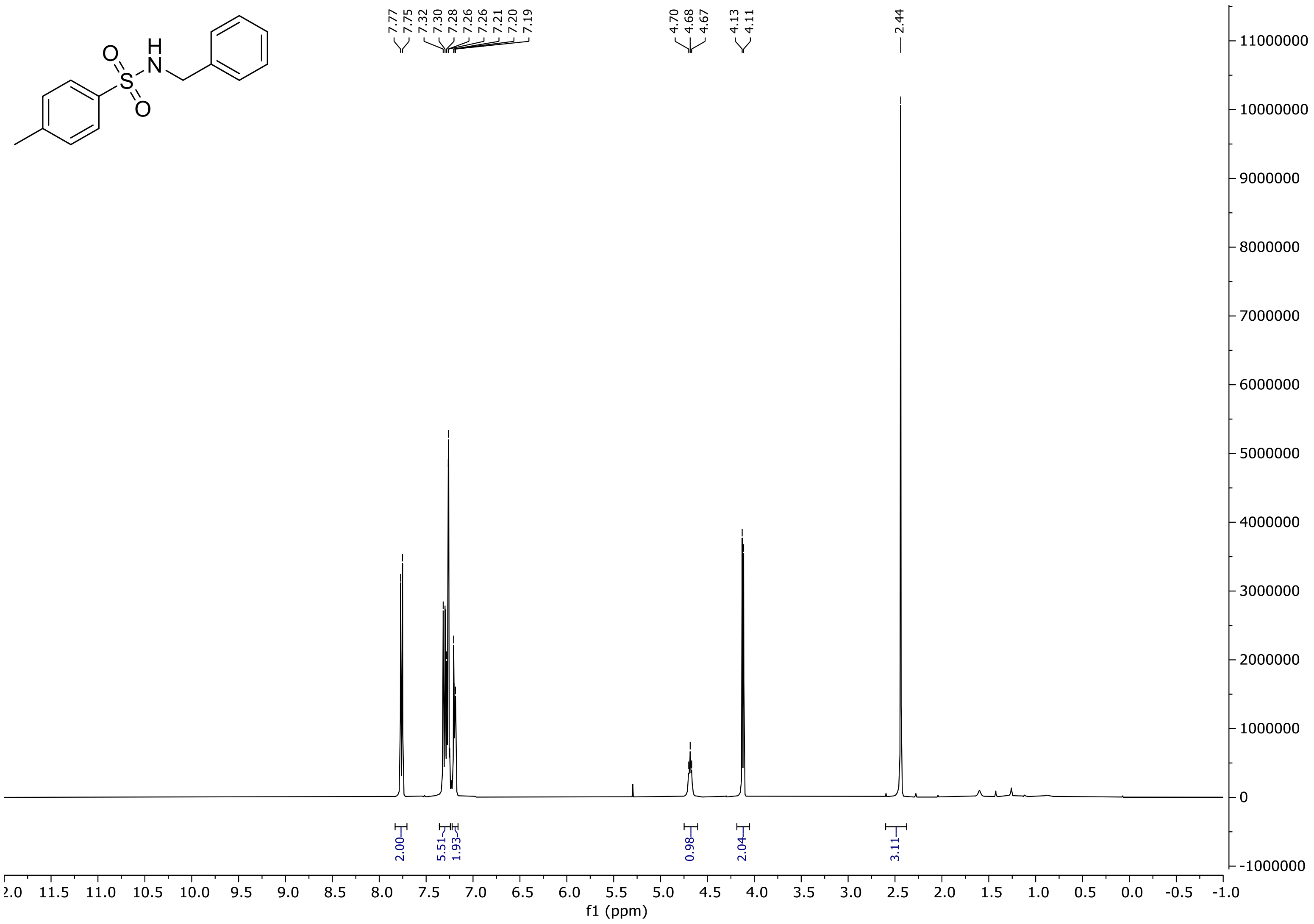

Figure S75. ^{1}H NMR (400 MHz) of **5e** in $CDCl_3$.

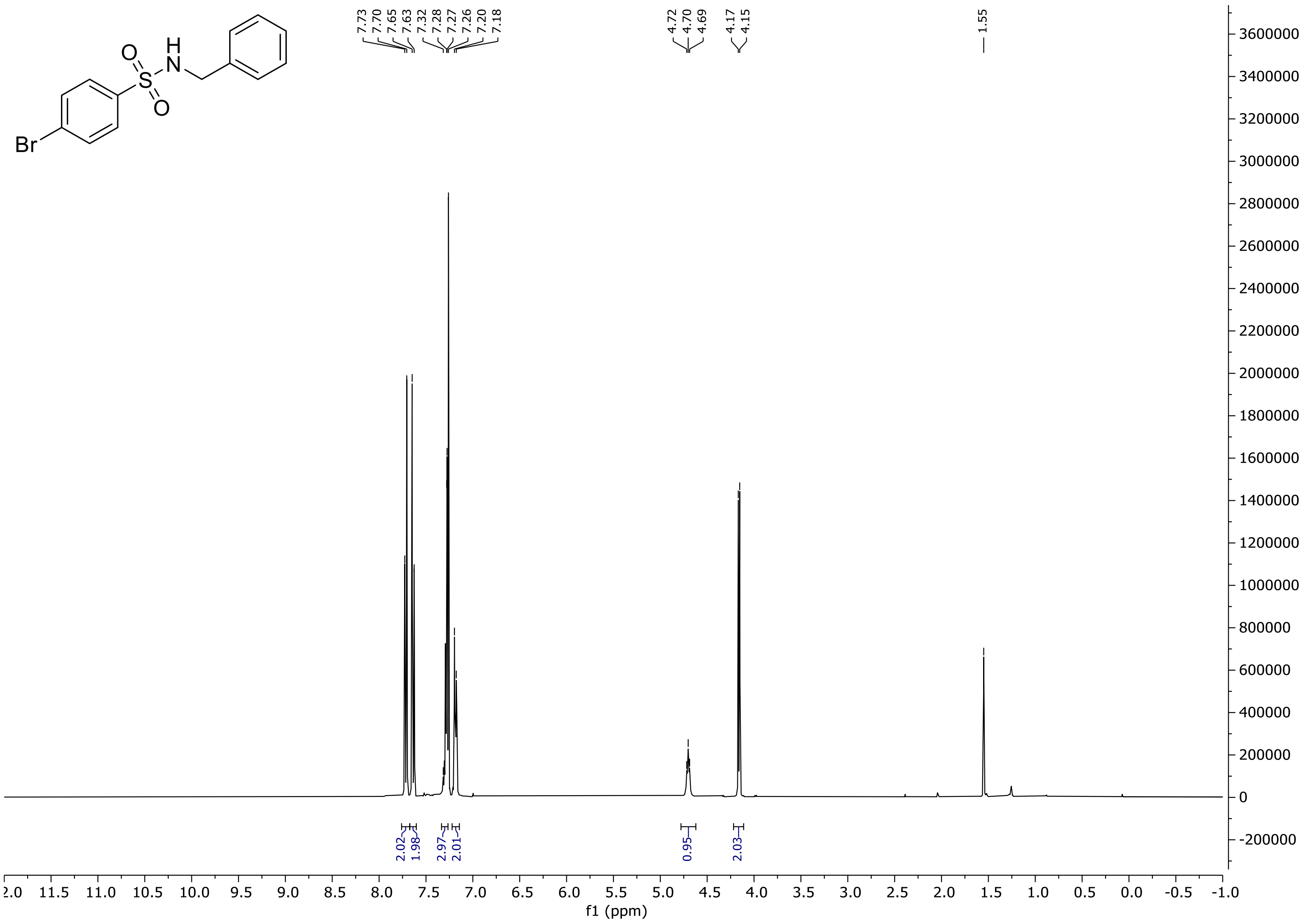

Figure S76.¹H NMR (400 MHz) of **5f** in $CDCl_3$.

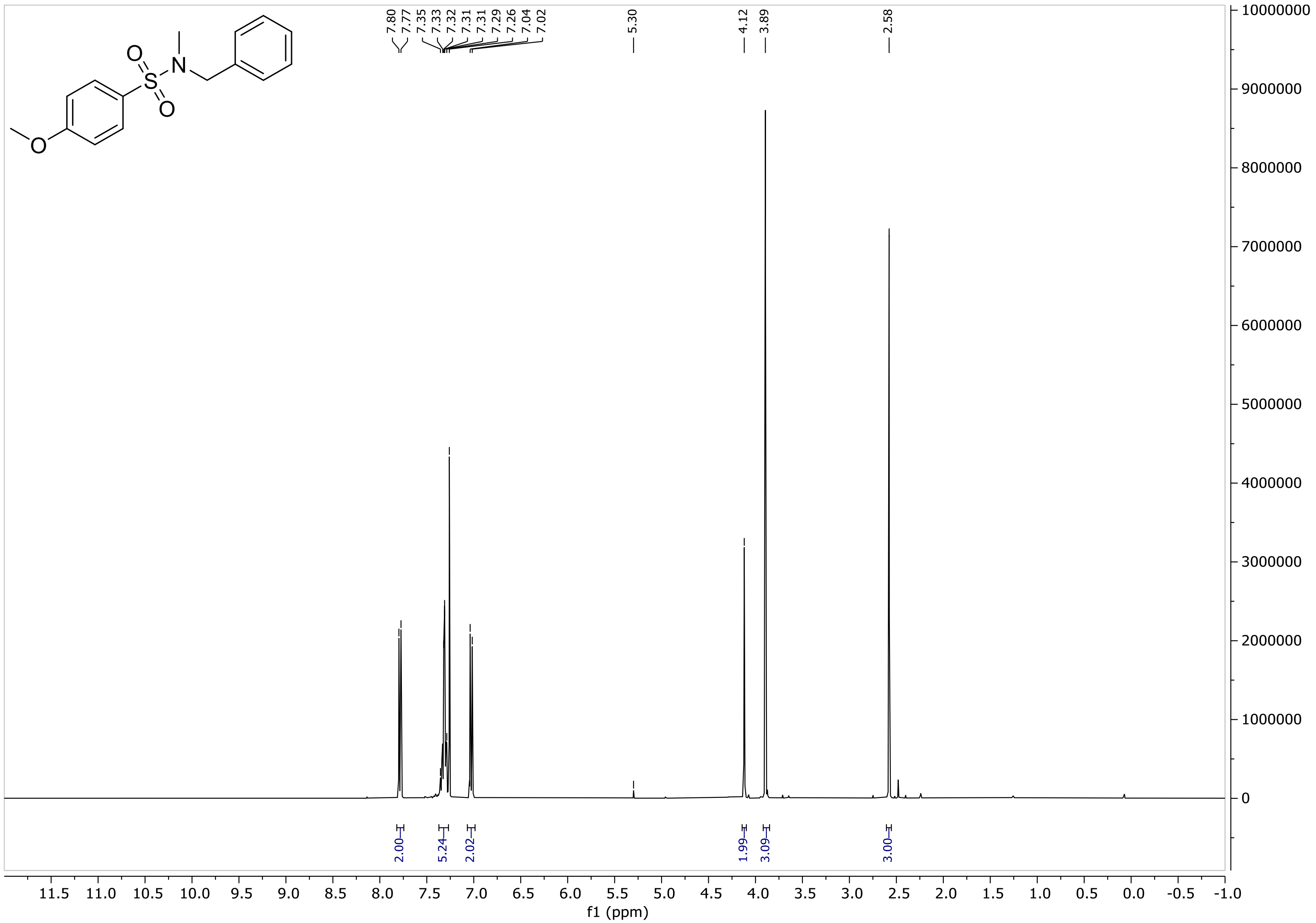

Figure S77. ^{1}H NMR (400 MHz) of **5g** in $CDCl_3$.

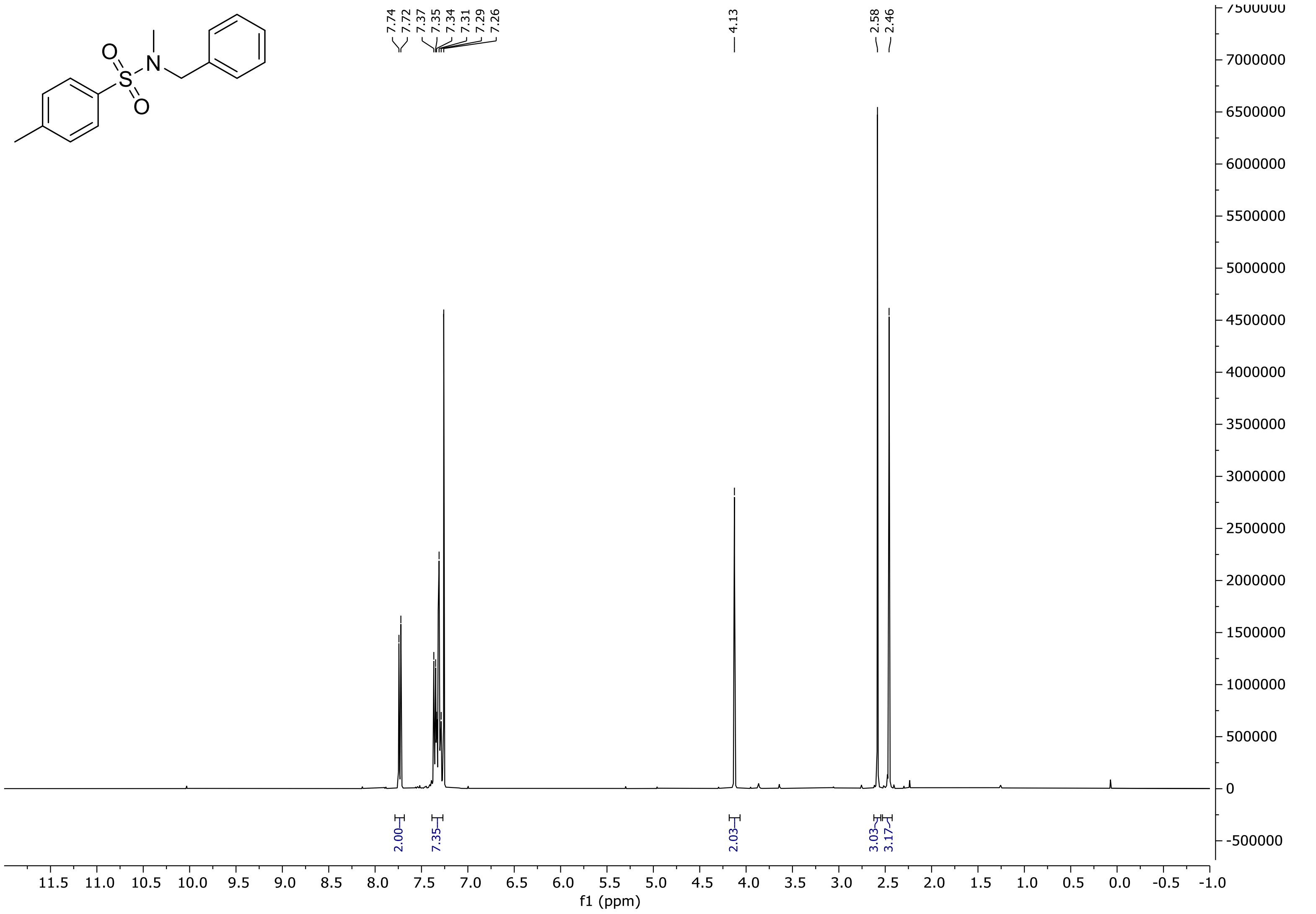

Figure S78. ^{1}H NMR (400 MHz) of **5h** in $CDCl_3$.

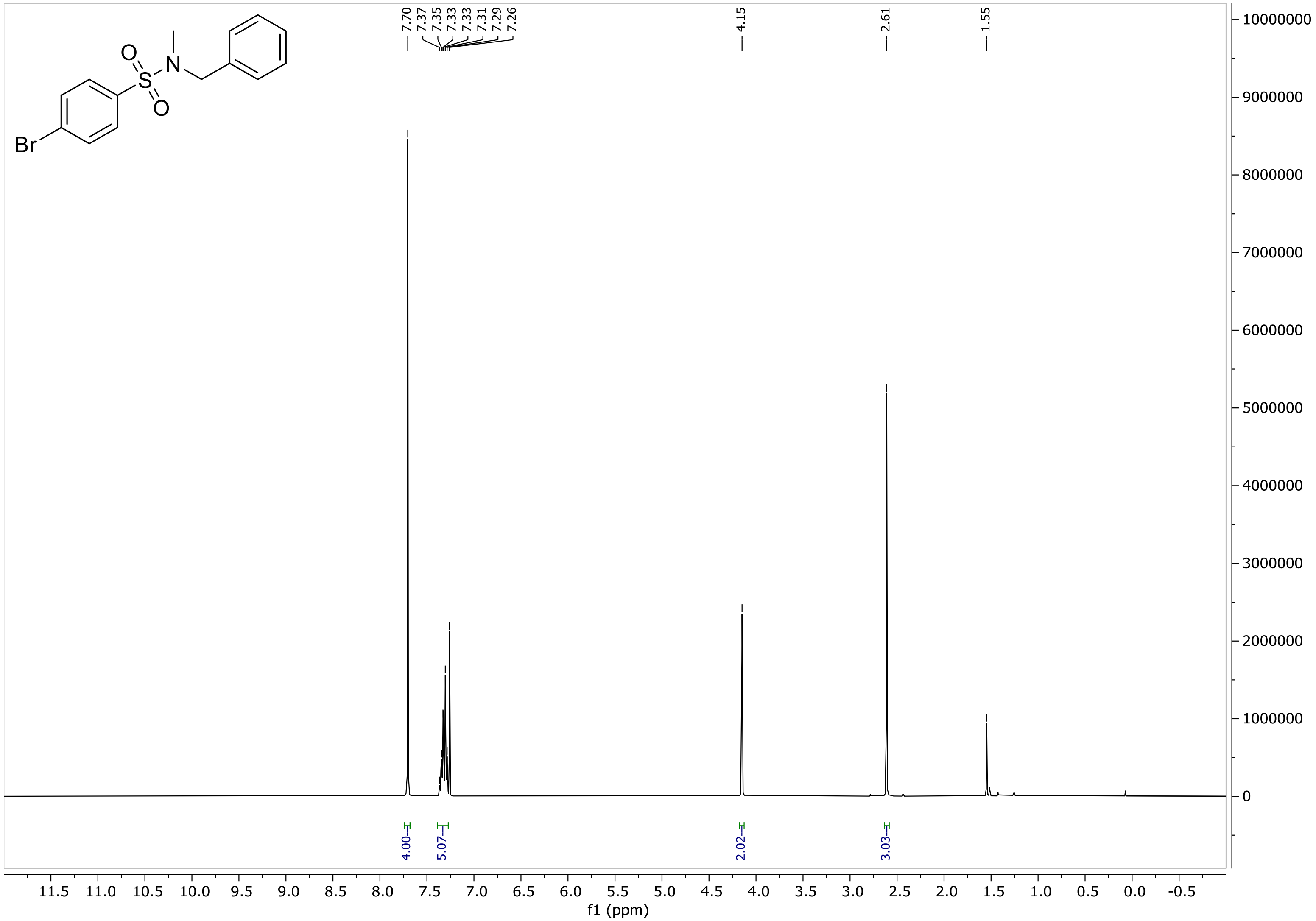

Figure S79. ^{1}H NMR (400 MHz) of **5i** in $CDCl_3$.
